# NanoScript-Enabled Nonviral Transient Repression of Phosphatase and Tensin Homolog for Axonal Regeneration and Central Nervous System Injury Repair

**DOI:** 10.1021/acsnano.5c13020

**Published:** 2026-02-19

**Authors:** Brandon Conklin, Yanting Liu, Sarah Nevins, Byeong-Gwan Song, Sy-Tsong Dean Chueng, Qiu Xiaowen, Sungyun Kim, Heyin Cheung, Seong Bae An, JongMin Lee, Bong Geun Chung, Wise Young, Dongming Sun, Hiroshi Sugiyama, Inbo Han, Ki-Bum Lee

**Affiliations:** † Department of Chemistry and Chemical Biology, 242612 Rutgers University, 123 Bevier Road, Piscataway, New Jersey 08854, United States; ‡ Department of Neurosurgery, 37129 Cha University School of Medicine, Seongnam-Si, Gyeonggi-do 13496, South Korea; § Department of Life Science, 451070 CHA University School of Medicine, 335 Pangyo-ro, Bundang-gu, Seongnam-si, Gyeonggi-do 13488, Republic of Korea; ∥ W.M. Keck Center for Collaborative Neuroscience and the Department of Cell Biology and Neuroscience, 242612 Rutgers University, Piscataway, New Jersey 08854, United States; ⊥ Department of Orthopedics, The Fourth Affiliated Hospital of School of Medicine, and International School of Medicine, International Institutes of Medicine, Zhejiang University, 866 Yuhangtang Road, Hangzhou 310058, China; # Department of Mechanical Engineering, Sogang University, 35 Baekbeom-ro, Mapo-gu, Seoul 04107,Republic of Korea; 7 Institute for Integrated Cell-Material Sciences (WPI-iCeMS), Kyoto UniversityYoshida, Ushinomiya-cho, Sakyo-ku, Kyoto 606-8501, Japan

**Keywords:** axon regeneration, spinal cord injury, nonviral gene therapy, artificial transcription factor, transient gene silencing, nanoparticle therapeutics, nanoparticle-based gene delivery, regenerative medicine

## Abstract

Spinal cord injury (SCI) remains a debilitating neurological disorder with limited therapeutic options, as existing treatments primarily address symptoms rather than address the complex interplay of cellular and molecular barriers to regeneration. These barriers collectively hinder functional recovery, including inhibitory glial scarring, chronic neuroinflammation, intrinsic neuronal regenerative deficits, and disruption of the blood-spinal cord barrier (BSCB). To address these limitations, we developed NanoScript-PTEN (NS-PTEN), a nonviral nanoparticle platform that delivers synthetic transcription factors to transiently suppress phosphatase and tensin homolog (PTEN) expression. PTEN negatively regulates the PI3K/AKT/mTOR signaling axis, which is a critical determinant of neuronal survival and axonal growth. By reducing PTEN levels, NS-PTEN derepresses this pro-survival pathway, promoting neuronal regeneration in the injured spinal cord. By integrating a DNA-binding domain targeting the PTEN promoter, a transcriptional repression module, and a nuclear localization signal onto a gold nanoparticle (AuNP) scaffold, NS-PTEN achieves transient control over PTEN repression, reactivating pro-regenerative signaling while minimizing the risks of tumorigenesis associated with permanent gene silencing. In a clinically relevant contusion SCI rat model, NS-PTEN induced a coordinated series of structural and microenvironmental improvements that collectively support spinal cord repair. Histologically, NS-PTEN enhanced axonal continuity and remyelination, as evidenced by denser NF-positive fibers and substantially greater MBP preservation than in both the injury and AuNP groups. Concurrently, NS-PTEN markedly attenuated astroglial and microglial reactivity, reducing GFAP^+^ border formation and diminishing Iba1^+^ inflammatory cell accumulation. At the vascular interface, NS-PTEN upregulated CD31 and occludin expression, indicating restored endothelial integrity and the reconstruction of tight junctions, which are critical for BSCB repair. In parallel, the inflammatory milieu shifted toward a regenerative phenotype, characterized by suppressed pro-inflammatory cytokine expression (IL-6, TNF-α, and iNOS) and elevated anti-inflammatory/neurotrophic factors (IL-10 and BDNF). These improvements are consistent with secondary, microenvironment-level benefits arising from acute neuronal PTEN repression rather than direct modification of non-neuronal cell types. Importantly, PTEN expression partially rebounded by DPI-28, aligning with the intended transient activity window of the nanoscript system and supporting its translational safety. Through this combination of precise, nonintegrative gene modulation and broad downstream remodeling, NS-PTEN addresses both intrinsic neuronal limitations and extrinsic inhibitory features of the SCI microenvironment.

## Graphical abstract

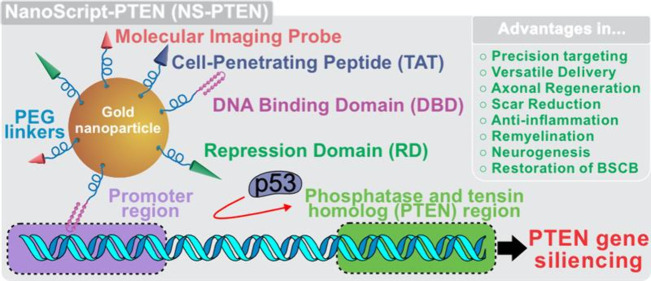

## 1 Introduction

Injuries to the central nervous system (CNS), particularly spinal cord injury (SCI), disrupt neural homeostasis through significant cellular dysfunction. These pathological changes drive persistent neurological deficits, which often continue chronically due to the CNS’s limited self-repair capacity.^1^ Post-SCI, inflammatory cascades, glial activation, and secondary injury foster a hostile microenvironment that impedes recovery and therapeutic intervention.^2^ A key challenge to recovery is gliosis, whereby reactive astrocytes proliferate and form a scar. Gliosis attempts to protect the CNS by maintaining BSCB integrity but simultaneously hinders recovery by releasing inhibitors that block axonal regeneration and neurite outgrowth. Furthermore, adult CNS neurons lack regenerative potential, having lost their developmental plasticity and axon-regrowth capacity. These challenges necessitate therapies targeting both inhibitory barriers and intrinsic neuronal limitations to drive axonal regeneration.

However, developing effective therapies to re-establish damaged neural circuitry presents significant challenges due to the complexity of the CNS and the multifaceted nature of its injuries. First, SCIs combine primary mechanical damage and secondary inflammatory cascades, triggering hemorrhagic necrosis and disrupting ascending/descending tracts critical for sensory/motor signaling.^3–6^ Second, reactive gliosis stabilizes the BSCB but paradoxically inhibits regeneration.^7–11^ Third, adult CNS neurons lack regenerative capacity, as developmental axon-growth programs are silenced postmaturation. Despite these barriers, studies identify molecular pathways—PTEN, Dual Leucine Zipper Kinase (DLK), insulin/Insulin-like Growth Factor-1 (IGF-1), and Suppressor of Cytokine Signaling 3 (SOCS3)—that reactivate neuronal plasticity. For instance, adeno-associated virus (AAV)-mediated PTEN deletion enhances axonal regeneration in optic nerve and SCI models by reactivating PI3K/AKT/mTOR signaling, a key driver of protein synthesis and growth, including activation of the mTOR downstream effectors S6K1 and p-S6, which directly regulate translational machinery and axonal elongation.^12,13^ Such advances highlight strategies to overcome inhibitory microenvironments and intrinsic regenerative deficits, targeting both extracellular and neuronal barriers to functional recovery.^14,15^ The PI3K/AKT/mTOR signaling pathway plays a pivotal role in the pathophysiology of SCI, regulating essential cellular processes such as survival, proliferation, migration, and protein synthesis.^16^ Notably, its activation has been shown to attenuate postinjury inflammation, reduce glial scar formation, and enhance axonal regeneration, ultimately improving functional recovery in preclinical models.^17^ PTEN acts as a negative regulator of this pathway by dephosphorylating phosphatidylinositol (3,4,5)-trisphosphate (PIP3), suppressing downstream AKT/mTOR signaling. For these reasons, targeted inhibition of PTEN has emerged as a promising strategy to reactivate PI3K/AKT/mTOR signaling and stimulate axonal regeneration after SCI. Experimental approaches include AAV-mediated gene silencing, short hairpin RNA (shRNA) delivery, and biomaterial-based systems. However, translating these strategies into clinical applications faces significant hurdles. For gene therapies, challenges include the heterogeneity of AAV serotypes in targeting specific cell populations, immunogenicity risks, and the necessity for transient PTEN repression rather than permanent knockout to avoid tumorigenesis. While effective in preclinical studies, shRNA-based methods are limited by technical complexities in vector production and inconsistent knockdown efficiency across neuronal subtypes.^18^ These limitations have spurred interest in biocompatible nanotechnology as an alternative platform for precision delivery. To this end, engineered nanomaterials can be designed to provide transient control over PTEN inhibition while simultaneously addressing secondary injury mechanisms for clinically viable therapies that restore neural connectivity after SCI.

To overcome the limitations of current SCI therapies and exploit the potential of nanobiotechnology-based, nonviral gene manipulation, we developed NanoScript-PTEN (NS-PTEN) (Figure 1A), a nanoparticle-based synthetic transcription factor designed to safely and efficiently promote axonal regeneration. NanoScript-PTEN overcomes limitations of RNA-based gene silencing approaches (both AAV and nonviral systems) by functioning as a synthetic transcription factor that directly suppresses PTEN mRNA transcription. This upstream mechanism of action contrasts with post-transcriptional RNA interference strategies, which require mRNA synthesis before translational inhibition is achieved, resulting in a lag between treatment and functional suppression of the target protein. NanoScript is a modular platform composed of gold nanoparticle (AuNP) functionalized with three interchangeable, PEGylated domains: (i) a DNA-binding domain (DBD) that specifically targets the Pten promoter region,^19^ (ii) a repression domain (RD) that blocks transcriptional machinery assembly at the binding site,^20^ and (iii) a nuclear localization sequence (NLS) enabling cellular and nuclear uptake. By transiently repressing *Pten* expression, NS-PTEN reactivates the PI3K/AKT/mTOR pathway (Figure 1B). NS-PTEN’s transient action minimizes oncogenic risks linked to permanent PTEN inhibition, making it particularly suited for regenerative applications requiring transient control, such as in vivo neural repair. Furthermore, the results from in vitro and in vivo testing show a reduction in pro-inflammatory cytokine expression in astrocytes and decreased glial scar formation. In this study, we further evaluated NS-PTEN’s therapeutic potential in a rodent thoracic SCI model (Figure 1C). Building on prior advances in nanoparticle delivery, we tested two administration strategies: (i) direct intracortical injection to stimulate corticospinal tract (CST) axon regrowth and (ii) retrograde transport-mediated delivery, a clinically relevant approach for targeting spinal neurons. These dual routes were selected to assess both localized and systemic therapeutic efficacy, bridging preclinical innovation with translational feasibility.

**Figure 1:**
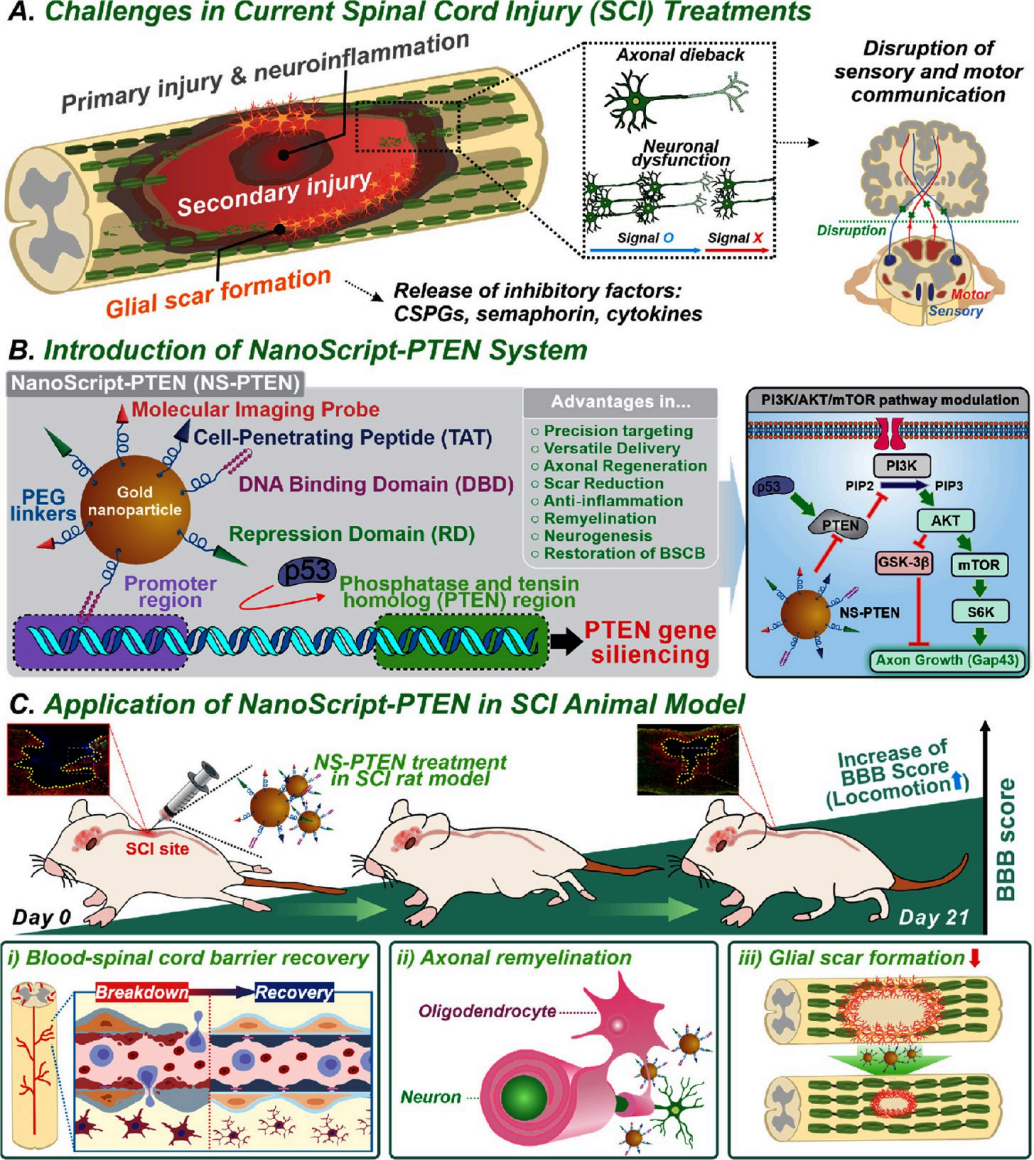
Repression of PTEN in SCI rats promotes axonal regeneration by modulating the PI3K/AKT/mTOR pathway. (A) Schematic illustration depicting key challenges that need to be overcome to allow for functional recovery following SCI. (B) NS-PTEN platform components and mechanism of action. Suppressing PTEN modulates the PI3K/AKT/mTOR pathway and promotes axonal regeneration. (C) Injection of NS-PTEN into the injury site of rats increases their locomotive abilities while promoting BSCB recovery and remyelination of axons and decreasing the glial scar area.

In short, our study explores nonviral gene silencing as a strategy to overcome the regenerative limitations of SCI. By selectively and transiently repressing key inhibitory regulators, such as PTEN, we could modulate critical signaling pathways—such as PI3K/AKT/mTOR—that govern axonal growth and neuroplasticity. This approach directly addresses SCI’s dual challenges: the inhibitory extracellular microenvironment and the intrinsic regenerative deficits of mature neurons. Unlike traditional therapies, our nanoparticle-based platform enables transient control over gene regulation, minimizing the risks associated with viral vectors or permanent genetic alterations. By silencing PTEN through reducing mRNA formation, we create a permissive molecular environment that enhances axonal regeneration and functional recovery that represents a paradigm shift in regenerative medicine, moving beyond symptomatic management to target the root causes of SCI pathology.^5,21,22^ Furthermore, the modularity of the NanoScript platform—its modular architecture for synthetic transcription factor design and nanoparticle-based delivery—enables rapid adaptation to other disease-relevant targets. This approach offers a generalizable framework for treating complex neurological disorders through programmable gene regulation, demonstrating the translational potential of synthetic biology-based nanotherapeutics.

## 2 Results and Discussion

### 2.1 Development and Characterization of NS-PTEN: A Nonviral Gene Silencing Platform for SCI Repair

Our NanoScript platform was designed to mimic the structure and function of natural TFs to enable precise gene regulation (Figure 2). Specifically, to achieve PTEN repression, the system incorporates three modular domains critical to TF activity: (i) DBD targeting the PTEN promoter, (ii) RD blocking transcriptional machinery assembly, and (iii) NLS ensuring cellular and nuclear delivery. By emulating these domains, NS-PTEN selectively binds to the p53 response element within the PTEN promoter, effectively suppressing its transcriptional activity (Figure 2A). To target our system toward the PTEN promoter region, a hairpin polyamide was designed to recapitulate the DBD on NS-PTEN. This domain is comprised of pyrrole (Py) and imidazole (Im) groups (ImPyPy-β-ImPy-γ-ImPy-β-PyImPy-β-Dp-NH_2_; β is β-alanine, γ is γ-aminobutyric acid, and Dp is dimethylamino propylamine) that target PTEN promoter sequences (GCAAGC and GCATGC) and repress PTEN activity (Figure 2B). This design recapitulates the specificity of natural TFs while circumventing off-target effects, enabling precise transcriptional repression.^23,24^ The specificity for PTEN is due to the specific binding of the DBD to the PTEN promoter sequence in the minor groove of DNA.^25^ By tailoring the molecular sequence to selectively bind GC or AT base pairs, DBDs can be engineered to target specific genomic loci and DNA conformations. For PTEN NS formation, amine-terminated biomolecules were conjugated to a linker molecule, SH-PEG-COOH. To activate the carboxyl group, 1-ethyl-3-(3-(dimethylamino)­propyl) carbodiimide (EDC) and *N*-hydroxysuccinimide (NHS) were added sequentially to the PEG linker. The various domains (i.e., DBD, RD [WRPW–OH], and NLS [TAT]) were each added to the activated PEG linker separately. Finally, a mixture containing 30% SH-PEG-RD, 30% SH-PEG-DBD, 30% SH-PEG-NLS, and 10% SH-PEG-COOH was added to a 4 mg/mL 10 nm AuNPs solution to create NS-PTEN.

**Figure 2:**
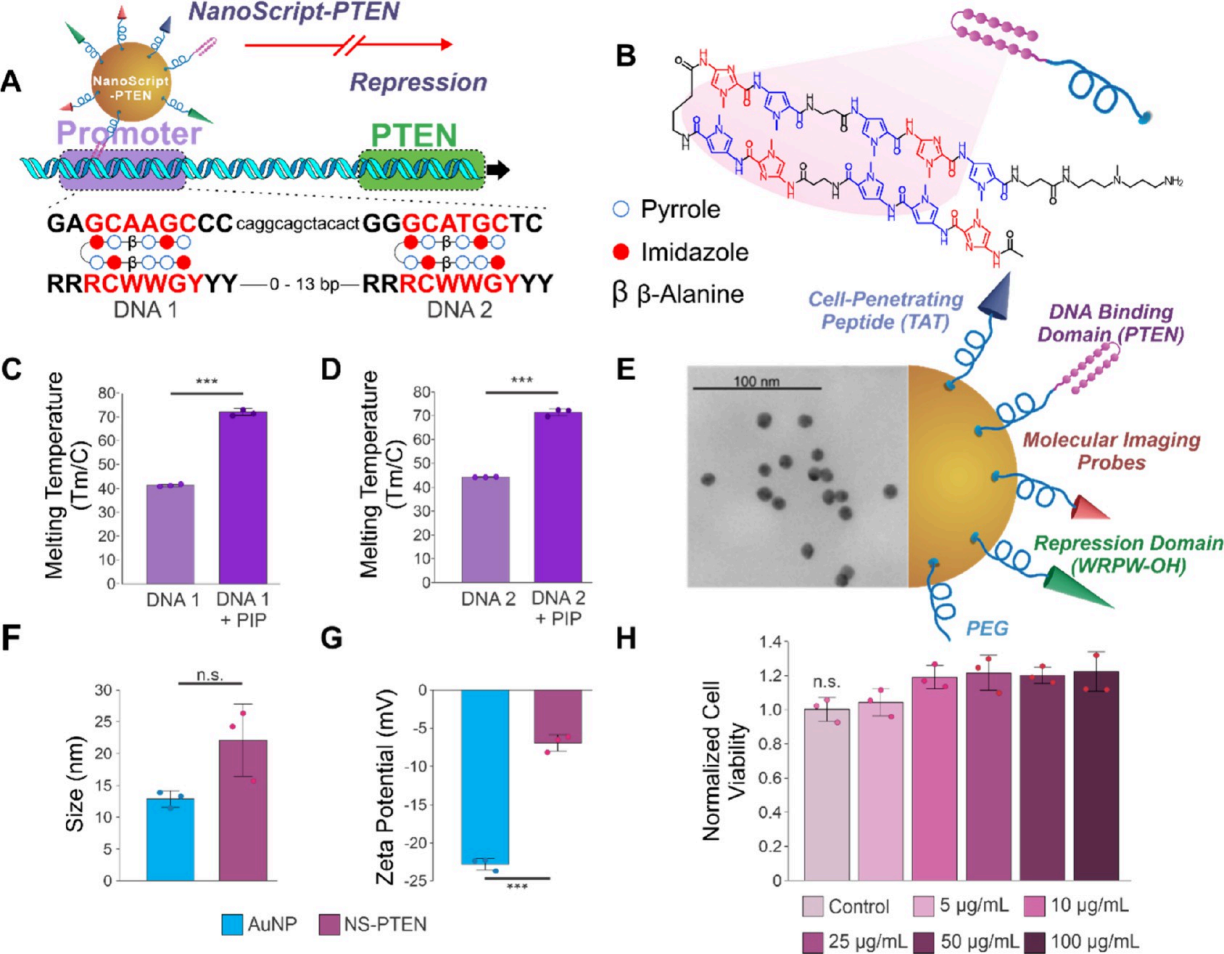
Design and characterization of NS-PTEN. (A) Schematic illustration of NS-PTEN interacting with the promoter regions of the *Pten* gene. (B) Structure of the hairpin polyamide DBD with pyrrole (blue) and imidazole (red) motifs arranged to promote sequence recognition. (C, D) Melting temperature shift assay (*n* = 3) demonstrating an increase in the thermal stability of the dsDNA following complementary binding by the DBD. (E) Representative TEM image of NS-PTEN, scale bar = 100 nm. (F) DLS measurements (*n* = 3) of the AuNP control and NS-PTEN. (G) Zeta potential measurements (*n* = 3) of AuNP and NS-PTEN. (H) Normalized cell viability (*n* = 3) demonstrating NS-PTEN is nontoxic to iPSC-NPC-derived neurons. Mean ± SEM, *t* test, one-way ANOVA, and Tukey’s test. ***P < 0.001; n.s. not significant.

A melting temperature shift assay was conducted to ensure that the designed DBD could recognize the target promoter sequence. Our findings indicate there is a significant (*P* < 0.001) increase in the melting temperature recorded when the dsDNA sequences (41.34 ± 0.4 C and 44.23 ± 0.1 C) are incubated with the DBD (72.2 ± 1.14 C and 71.37 ± 1.2 C) (Figure 2C,D, and Figure S1). To prevent the formation of basal transcriptional machinery at the binding sites, we employed a previously established WRPW peptide^26^ as the NanoScript RD. In summary, functionalized NS-PTEN was formed by assembling each transcription factor mimicking component (i.e., DBD, RD, and NLS) onto AuNPs using an established protocol.^27^ Upon conjugation of the various domains, TEM images (Figure 2E) and dynamic light scattering (DLS) measurements were collected to ensure the attachment and stability of NS-PTEN. The hydrodynamic diameter of the AuNP (12.87 ± 1.27 nm) was increased following ligand attachment (22.12 ± 5.65 nm) (Figure 2F) but remained small enough to allow entry through the nuclear pore complex.^28^ Also, the zeta potential increased significantly (*P* < 0.001) when the surface of the AuNPs (−22.8 ± 0.78 mV) was functionalized with the PEGylated domains (−6.94 ± 1.08 mV) (Figure 2G). Finally, we ensured the NS-PTEN platform was nontoxic to human-induced pluripotent stem cell neural progenitor cell (hiPSC-NPC)-derived neurons with a PrestoBlue assay, where there was no significant change (*P* > 0.05) in viability up to a delivered concentration of 100 μg mL^–1^ (Figure 2H). These results validate the successful synthesis of NS-PTEN and establish its ability to selectively and safely engage with PTEN promoter sequences, which is a critical feature for precision gene regulation. Building on this foundational finding, we next sought to characterize the downstream genetic alterations and phenotypic consequences of NS-PTEN delivery in neuronal populations. Specifically, we evaluated transcriptional changes in PTEN-associated pathways and correlated these with functional outcomes, such as neurite outgrowth and axonal elongation, comprehensively assessing the platform’s therapeutic potential.

### 2.2 In Vitro Validation of NS-PTEN’s Axon-Regenerative Capacity: PTEN Silencing and PI3K/AKT/mTOR Pathway Activation

To validate NS-PTEN’s therapeutic potential in a human-relevant system, we employed a hiPSC-NPC model, differentiated into mature neurons over a 7 day period (Figure S3A). This model recapitulates critical features of human neurons, enabling robust assessment of axonal regeneration mechanisms. Neurons were treated with either 10 μg mL^–1^ AuNP controls or NS-PTEN, followed by the analysis of key genes associated with axonal regeneration and neurite outgrowth. Specifically, we quantified expression levels of *Pten* and core components of the PI3K/AKT/mTOR pathway that govern neuronal growth and plasticity (Figure S3B–G). This approach allowed us to systematically evaluate NS-PTEN’s ability to modulate regenerative pathways while minimizing confounding variables inherent to in vivo systems.

Gene expression levels quantified by RT-qPCR revealed that NS-PTEN significantly (*P* < 0.01) reduced the expression of the direct target, *Pten*, to 0.503 ± 0.25 relative to the control group treated with AuNPs, which showed a relative expression level of 1.0 ± 0.16 (Figure S3B). Additionally, there was no significant change (*P* > 0.05) in the expression of *pi3k* in the AuNP (1.00 ± 0.14) or NS-PTEN (1.056 ± 0.17) conditions, as PTEN antagonizes the action of PI3K but does not directly act upon this kinase (Figure S3C).^29^ Interestingly, there were significant increases in genes further downstream, including *Akt* [AuNP (1.00 ± 0.059) and NS-PTEN (1.69 ± 0.23); (*P* < 0.001)], *mtor* [AuNP (1.00 ± 0.057) and NS-PTEN (3.94 ± 0.66); (*P* < 0.001)] and *gap43* [AuNP (1.00 ± 0.083) and NS-PTEN (1.705 ± 0.183) (*P* < 0.01)], which are responsible for mediating neuronal survival, controlling growth and metabolism, and axonal regeneration, respectively (Figure S3D,F,G). Similarly, there was a significant decrease (*P* < 0.05) in the gene *gsk-3*β [AuNP (1.00 ± 0.13) and NS-PTEN (0.65 ± 0.09)], which has been reported to promote axonal regeneration upon deletion^30^ (Figure S3E). Changes in cellular morphology (i.e., neurite outgrowth) are evident following NS-PTEN delivery as observed in Figure S3H. The activation of mTOR was determined by quantifying an established marker, phospho-S6 (p-S6). To this end, iPSC-NSC-derived neurons exhibited significantly (*P* < 0.0001) more p-S6 when treated with NS-PTEN (2.76 ± 1.29 au) than the AuNP control (1.00 ± 0.55 au) (Figure S3I,J).

To develop an inflammatory model and better mimic diseased conditions, we delivered LPS to the iPSC-NPCs for 24 h prior to NS-PTEN delivery for 24 h (Figure 3A). Western blot was conducted to validate the pathway of NS-PTEN function with pTEN, the pAKT/AKT ratio, and PI3K (Figure 3B). There was significant downregulation of pTen with NS-PTEN delivery at 10 μg/mL (0.4550 ± 0.1996) relative to LPS-induced cells (*P* < 0.05) (Figure 3C). PI3K was significantly upregulated at 10 μg/mL (1.830 ± 0.3210) relative to LPS-induced cells (*P* < 0.05) (Figure 3D). pAKT/AKT had significant upregulation at 10 μg/mL (2.926 ± 1.092)­relative to LPS-induced cells (*P* < 0.05) (Figure 3E). The proportions of apoptotic cells were quantified after treating iPSC-derived NPCs (Figure S4A–G). In the flow cytometry plots, the lower-right quadrant indicates early apoptosis, while the upper-right quadrant corresponds to late apoptosis. Thus, combining the LR and UR quadrants provides an effective measure of total apoptotic activity. The apoptosis assay showed significant decreases in the apoptosis levels of the LPS-induced neurons (70.36 ± 1.717) following NS-PTEN treatment at 10 μg/mL (5.247 ± 0.6694) (*P* < 0.0001), and furthermore, also showed a significant decrease in apoptosis from the AuNP control at 10 μg/mL (23.53 ± 0.7637) (*P* < 0.0001) (Figure S4A–G). Cell Viability significantly increased upon NS-PTEN delivery (1.283 ± 0.03594) to LPS-treated cells (0.5750 ± 0.04041) (Figure S4H). To confirm that the PI3K/AKT/mTOR signaling axis is the mechanism of neuron degeneration, rapamycin was codelivered with the AuNP control and NS-PTEN. Treatment with NS-PTEN (10 μg/mL) induced a significant upregulation in the phospho-mTOR/mTOR ratio (2.257 ± 0.7168) compared to control groups (*P* < 0.0001), confirming successful activation of the PI3K-AKT-mTOR pathway. Crucially, this effect was completely abrogated by cotreatment with rapamycin, an allosteric mTOR inhibitor, demonstrating the specificity of NS-PTEN action through the canonical mTORC1 signaling axis (Figure S4I,J). Moreover, AuNP controls showed no significant change in the ratio of pmTOR/mTOR without rapamycin delivery, further displaying the therapeutic effects' dependence on NS-PTEN formation. Immunostained images also show that axonal regeneration by NS-PTEN is inhibited in the presence of rapamycin (Figure S4k). Collectively, these findings demonstrate that NS-PTEN potently modulates the PI3K/AKT/mTOR signaling axis to promote neuronal survival and axonal growth in healthy neurons and following induction of inflammation in mature neurons. The concordance between genetic pathway activation and functional morphological changes underscores the platform’s capacity to reprogram neuron regenerative signaling. Given these robust in vitro results, we translated these findings into in vivo studies using a rodent contusion SCI model to assess the therapeutic translatability of NS-PTEN.

**Figure 3:**
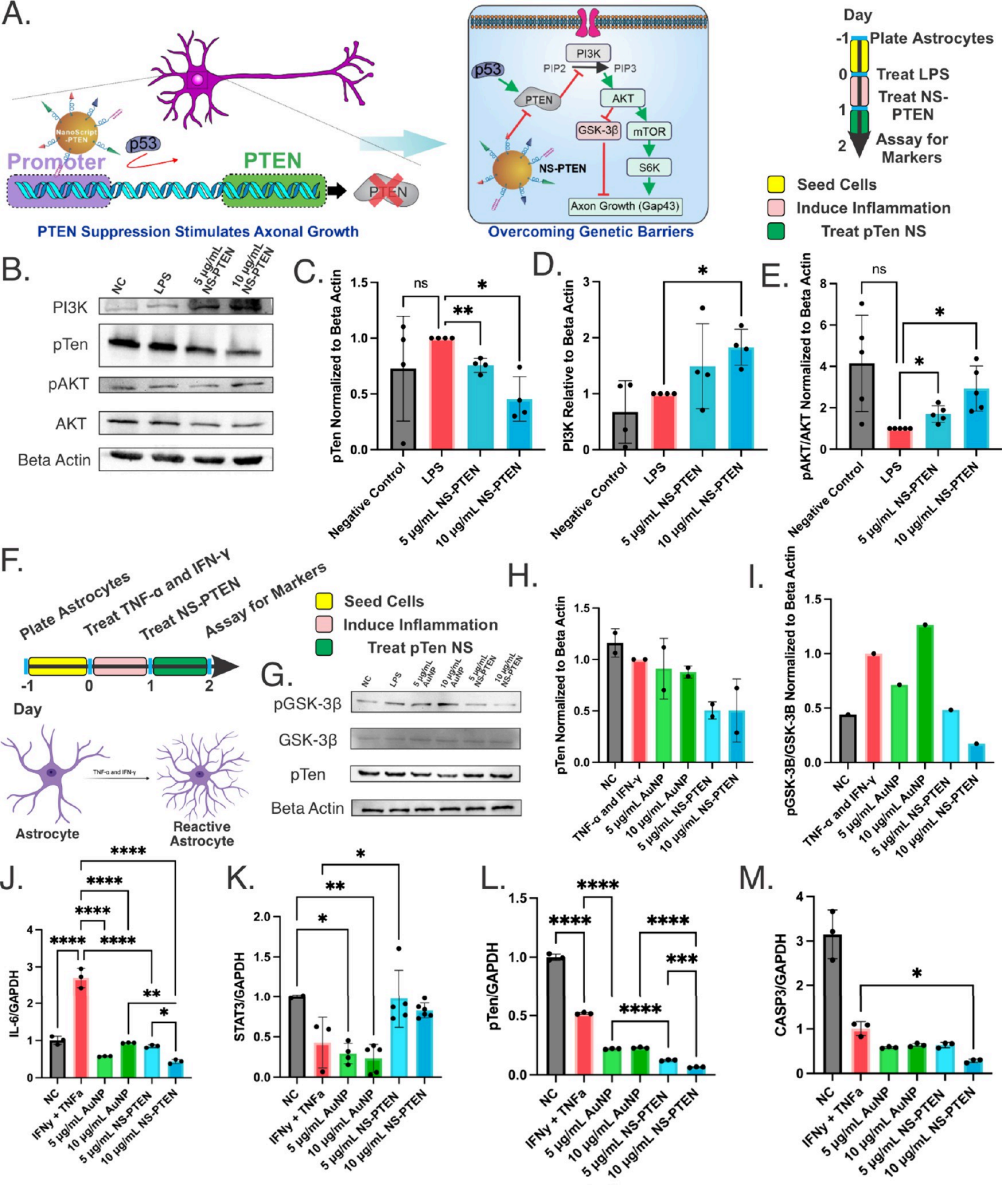
*In vitro* validation of NS-PTEN promoting axonal regeneration,activating the mTOR pathway, and astrocyte reduction in pro-inflammatory cytokine expression. (A) Schematic illustration depicting biological entities involved in the PI3K/mTOR/AKT pathway, their role in axonal regeneration following SCI, and experimental timeline. (B)­Western blot following induction of cells with LPS as well as delivery of pTen NS. (C) Quantification of decrease in pTen expression following NS-PTEN delivery relative to beta actin. (D) Quantification of decrease in PI3K expression following NS-PTEN delivery relative to beta actin. (E) Quantification of decrease in pAKT/AKT expression following NS-PTEN delivery relative to beta actin. (F) Schematic for astrocyte activation by TNF-α and IFN-γ and experimental timeline. (G) Western blot following induction of cells with TNF-α and IFN-γ as well as delivery of NS-PTEN. (H) Quantification of decrease in pTen follow NS-PTEN delivery, (I) Quantification of decrease in pGSK-3β/GSK-3β. RT-q-PCR results demonstrated (J) IL-6, (K) STAT3, (L) NS-PTEN, and (M) CASP3 effects on astrocytes.

To study NS-PTEN effects on astrocytes, human astrocytes were induced with TNF-α and IFN-γ to study the NS-PTEN ability to reduce pro-inflammatory cytokine levels following pTen downregulation (Figure 3F). There is conflicting research on the effects of AKT and mTOR activation in astrocytes following SCI.^31,32^ In general, mTOR dysregulation in astrocytes leads to astrocyte activation. Therefore, to fully understand the effects of NS-PTEN on astrocyte reactivity, a pro-inflammatory model consisting of TNF-α and IFN-γ was applied to human astrocytes, and NS-PTEN or AuNPs were delivered for 24 h (Figure 3F).

An apoptosis assay utilizing Annexin V and APC showed significant decreases in the apoptosis levels of the TNF-α and IFN-γ treated astrocytes following NS-PTEN treatment (*P* < 0.0001), and furthermore, also showed a significant decrease in apoptosis from the AuNP control also treated with TNF-α and IFN-γ (*P* < 0.0001) (Figure S5A–G). *Casp3,* which is a key regulator of apoptosis, decreased significantly upon NS-PTEN delivery at 10 μg/mL (*P* < 0.05) (Figure S5H). Delivering 10 μg/mL NS-PTEN (*P* < 0.05) led to a significant decrease in PTEN protein levels (0.5050 ± 0.3066) relative to the TNF-α and IFN-γ treated control (Figure 3G,H). The ratio of pGSK-3β/GSK-3β also significantly decreased (*P* < 0.05) upon 10 μg/mL NS-PTEN delivery relative to the TNF-α and IFN-γ treated control (Figure 3G,I). Upon AuNP and NS-PTEN delivery at 10 μg/mL to TNF-α and IFN-γ treated cells, RT-q-PCR results showed significant downregulation (*P* < 0.0001) of *il-6* [TNF-α and IFN-γ (2.703 ± 0.2613) vs NS-PTEN(0.8454 ± 0.03822)], *pTen* [TNF-α and IFN-γ (0.5185 ± 0.007504) vs NS-PTEN(0.06519 ± 0.004143)], and *il-1*β [TNF-α and IFN-γ (1.031 ± 0.3271) vs NS-PTEN (0.2611 ± 0.04745)] (Figure 3J,L,M). There was also a significant decrease in *il-6* from the AuNP control to NS-PTEN (*P* < 0.01) when both were delivered at the optimal treatment concentration of 10 μg/mL (Figure 3J). By reducing pro-inflammatory cytokine expression, as seen with *il-6* and *il-1*β as well as a decrease in pGSK-3β/GSK-3β, astrocyte pro-inflammatory cytokine expression is decreasing. Furthermore, a nonsignificant change in *stat3* confirms that there is not a significant increase in astrocyte pro-inflammatory status following a decrease in pTen expression in the cells, as STAT3 is a key indicator of an increase in astrocyte reactivity following AKT/mTOR activation (Figure 3K). Combined, our data shows that in vitro, there is a decrease in pro-inflammatory cytokine release from astrocytes, which is ideal for a reduction in glial scar formation.

### 2.3 Antegrade Delivery of NS-PTEN Promotes Corticospinal Tract Regeneration via Transient PTEN Repression in a Contusion SCI Model

Having validated NS-PTEN’s in vitro efficacy in suppressing PTEN, reactivating PI3K/AKT/mTOR signaling, and promoting neurite growth, we next assessed its in vivo therapeutic potential. A key translational hurdle is delivering gene-editing agents to sensorimotor neurons critical for motor control and proprioception, many of which have long spinal projections that are vulnerable to injury. Their limited regenerative capacity makes them a key SCI therapeutic target. Thus, we tested NS-PTEN’s ability to achieve targeted delivery and enhance regeneration in a clinically relevant injury model, employing anterograde and retrograde delivery strategies. To this end, we delivered NS-PTEN conjugated with PEGylated Alexa Fluor 568 (10 nM) into the sensorimotor cortex through intracranial injection and sacrificed the rats after 1 week. Immunohistochemistry was performed on cortical sections using antibodies for NeuN, glial fibrillary acidic protein (GFAP), and p-S6 to determine which cell types are internalizing NS-PTEN. Immunostaining for NeuN and GFAP validated that among the 245 cortical neurons and 276 astrocytes measured, there is a significant (*P* < 0.05) preference for NS-PTEN to be internalized by neurons (26.94%) as opposed to astrocytes (19.57%) (Figure S5). It is also important to note that glial cells outnumber neurons in the brain by a ratio of approximately 2:1 to 3:1 and are less metabolically active. This difference in cell populations and metabolic activity may contribute to the preferential internalization of the platform by neurons.^33^ Additionally, confocal imaging confirmed that NS-PTEN was present within the nuclei of cortical cells that are both NeuN and p-S6 positive (Figures S6–S8).

Upon confirming the preferential neuronal internalization of NS-PTEN *in vivo*, we sought to investigate whether NS-PTEN could effectively stimulate CST regeneration in the context of a contusion SCI. This SCI model destroyed all passing axons and did not leave any local neuronal connections intact. Rats were divided into two groups and were injected with 10 μL of either NS-PTEN or AuNP control into their sensorimotor cortex in both hemispheres directly following SCI (Figure S9A). We observed the same trend noted *in vitro,* where there was a significant (*P* < 0.001) decrease in *Pten* expression [AuNP (1.00 ± 0.20) and NS-PTEN (0.37 ± 0.06)] and no significant (*P* > 0.05) change in *pi3k* expression [AuNP (1.00 ± 0.23) and NS-PTEN (1.08 ± 0.12) (Figure S9B,C)]. To further evaluate the effect of NS-PTEN on the integrity of the CST, a transverse cross-section of the spinal cord was imaged 3 mm rostral to the injury site (Figure S9E). The image revealed a higher density of intact CST axons in the NS-PTEN-treated hemisphere. This suggests that NS-PTEN treatment mitigated retrograde axonal dieback proximal to the lesion.

To quantify the degree of axon growth through the lesion area, sagittal images of the CST were collected and analyzed. Complete contusion SCIs produce a cavity filled with necrotic tissue surrounding the initial injury site. Therefore, we defined the rostral margin of the cavity as the baseline for measuring the distance of the CST regeneration. In 86% (6 out of 7) of AuNP control rats, no labeled axons were observed reaching the rostral margin of the lesion area. In contrast, four of seven rats treated with NS-PTEN had their main CST bundle extend beyond the rostral margin of the lesion (Figure S9F). In fact, there was a significant (*P* < 0.01) increase in the distance axons grew through the lesion area in the NS-PTEN-treated condition (268.65 ± 402.86 μm) relative to the AuNP-treated condition (−492.64 ± 379.29 μm) (Figure S9G). The remaining three rats had CST bundles that approached closer to the rostral margin than the control group. Overall, axons in the CST from rats treated with NS-PTEN grew farther than those treated with the AuNP control. These findings indicate that NS-PTEN can stimulate CST regeneration after a contusion SCI. However, despite this observed regeneration, there was no clear evidence of enhanced locomotive abilities in these rats when assessed using the BBB score. This is likely because the CST in both the treatment and control groups did not traverse the injury site to re-establish connections with subordinate neurons within the short observation period.

### 2.4 Microfluidic Platform for Modeling Axonal Injury and Assessing Retrograde Internalization of NS-PTEN

To overcome key limitations in the direct delivery of NS-PTEN to the motor cortex and enhance functional recovery in SCI rats, we investigated whether NS-PTEN could undergo retrograde transport—a mechanism enabling neuronal internalization from distal axon terminals. Demonstrating this capability would validate a clinically feasible strategy for delivering NS-PTEN directly to the injury site, bypassing invasive cortical administration. To test this hypothesis, we first evaluated NS-PTEN’s retrograde internalization and axonal regenerative effects in vitro. For this purpose, we engineered a compartmentalized microfluidic device using soft lithography, featuring two parallel microchannels connected by a narrow 30 μm-wide bridge (Figures S10A and S11). This design spatially segregates mouse neural stem cells (mNSCs) in the left chamber from their extending axons, which traverse the bridge and terminate in the distal chamber. The size-restricted bridge ensures exclusive axonal growth into the distal compartment, enabling a precise assessment of retrograde nanoparticle transport and its impact on axon regeneration.

To promote retrograde delivery, we added media containing NS-PTEN to the distal chamber, where the axons terminate. We observed that axonal outgrowth of the NCSs treated with NS-PTEN was significantly (*P* < 0.001) increased in the microfluidic model compared to the AuNP condition after 48 h [NS-PTEN (681.18 ± 133.47 μm) and AuNP (112.07 ± 54.75 μm)] (Figure S10C–E). In fact, a time course study conducted over 18 h demonstrates NS-PTEN’s ability to promote axonal growth, as there was a significant (*P* < 0.01) increase in the distance axons traveled into the distal chamber between 12 and 18 h post-treatment [12 h (300.46 ± 59.37 μm) and 18 h (579.58 ± 82.24 μm)] (Figures S10F and S12). While it is evident that NS-PTEN promotes axonal growth, we also wanted to use this device to test the regenerative capabilities of our platform. To this end, we used the neurotoxin thapsigargin (TG), which has been demonstrated to promote oxidative stress, lipid peroxidation, and inhibit the antioxidant gene Pink1 to model axonal degeneration^34,35^ (Figure S10B). Specifically, four experimental conditions were evaluated by measuring axonal growth in the distal chamber: untreated control, AuNP vehicle control, NS-PTEN treatment, and NS-PTEN with thapsigargin (TG) exposure. TG exposed then NS-PTEN treated [AuNP (141.33 ± 38.19 μm), NS-PTEN (343.33 ± 108.32 μm), NS-PTEN + TG (0 ± 0 μm), and TG + NS-PTEN (101.33 ± 37.00 μm)] (Figure S10G). Pretreating mNSCs with NS-PTEN prior to TG exposure resulted in pronounced axonal damage, with no axonal protrusion observed in the distal chamber across experimental groups. However, reversing the treatment sequence led to significant neuronal rescue and axonal regrowth. In this condition, axonal regeneration metrics (e.g., neurite length, branching complexity) showed no significant difference (*P* > 0.05) compared to AuNP-treated controls (Figure S13), indicating that NS-PTEN restores the regenerative capacity to levels observed in uninjured neurons. These findings demonstrate two key therapeutic properties of NS-PTEN: (i) it enhances axonal regeneration in healthy NSCs, and (ii) it rescues neurite outgrowth in neurons compromised by neurotoxic injury. The latter observation suggests that NS-PTEN can intervene in secondary injury cascades and motivated our transition to in vivo studies, where we delivered NS-PTEN directly to the SCI lesion site.

### 2.5 Acute-Phase Neuronal PTEN Downregulation via NS-PTEN’s Nuclear-Targeted Delivery Post-SCI

To establish a mechanistic and experimental framework for evaluating NS-PTEN–mediated functional recovery after spinal cord injury in vivo, we developed a targeted intraspinal delivery strategy guided by pathway-specific therapeutic insights derived from our in vitro experiment (Figure 4A). To better evaluate this strategy in vivo, we implemented a standardized SCI experimental timeline (Figure 4B). Adult rats were subjected to thoracic spinal cord contusion followed by immediate intraspinal injection of NS-PTEN, AuNP carrier control, or vehicle. Functional, histological, and molecular assessments were systematically performed across acute, subacute, and chronic phases, with behavioral analyses extending to DPI-28. This design enabled longitudinal correlation between early molecular intervention and late-stage motor outcomes.

**Figure 4:**
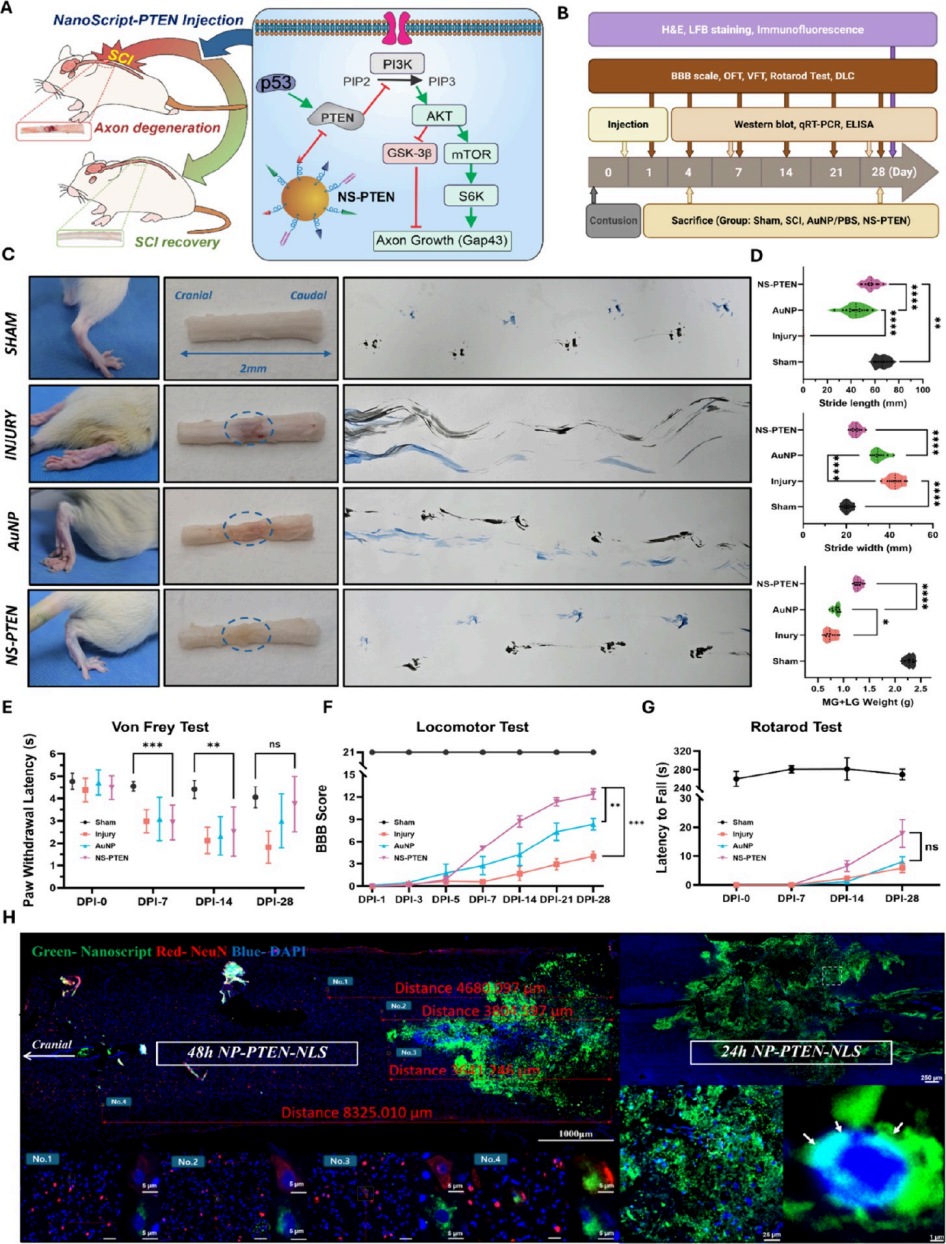
Experimental design, therapeutic mechanism, and functional evaluation of NS-PTEN in spinal cord injury. (A) Diagram depicting the molecular mechanism of NS-PTEN. (B) Timeline of the in vivo experimental procedures. (C) Footprint analysis and representative limb morphology following SCI and treatment. Gross hindlimb appearance and ink-based footprint gait tracking at DPI-28 are shown for Sham, Injury, AuNP, and NS–PTEN groups. Blue dashed circles indicate the lesion site. Blue and black dotted traces represent ipsilateral and contralateral hindlimb movements, respectively. (D) Quantification of gait parameters at DPI-28. Functional indicators, including stride width (mm), stride length (mm), and MG+LG (medial gastrocnemius + lateral gastrocnemius) muscle weight (g), were measured. (E) Mechanical pain sensitivity was assessed using the Electronic Von Frey test with withdrawal latency measured in seconds. Sham animals showed stable responses over time. (F) Locomotor recovery was assessed using BBB scoring. (G) Motor coordination and endurance were evaluated by the Rotarod test. (H). Long-range retrograde transport and neuronal nuclear uptake of NS-PTEN following intraspinal delivery. Data are presented as mean ± SD. Statistical significance was determined using ANOVA followed by Tukey’s post hoc test. *P < 0.05, **P < 0.01, ***P < 0.001, ****P < 0.0001; ns, not significant.

To investigate whether targeted, early-phase PTEN suppression can activate intrinsic regenerative pathways following SCI, we administered NS-PTEN directly into the contused spinal cord using a stereotactic microinjector system (Figure S14). Following SCI induction with a calibrated impactor, NS-PTEN was delivered into three defined parenchymal sites: the lesion epicenter and two peri-lesion positions located ∼1 mm rostral and caudal to the injury. This localized intraspinal approach leverages the retrograde transport capacity of injured axons to facilitate NS-PTEN internalization at the lesion interface without requiring invasive cranial access. By inducing a transient, spatially confined reduction of PTEN expression within the inhibitory microenvironment, this strategy allows us to determine whether reactivating endogenous regenerative signaling enhances axon extension and functional recovery following SCI.

Over a 7 day postinjection period, the fluorescence signal revealed a dynamic pattern of NS-PTEN accumulation and clearance at the injury site. (Figure S15A). Specifically, the in vivo biodistribution of NS-PTEN was monitored using the DaVinci imaging system to assess its localization and retention in the spinal cord regeneration experiment. At 24 h, an initial increase in fluorescence indicated early NS-PTEN localization, peaking at 48 h with a total radiant efficiency of approximately 2 × 10^14^ p/s/cm^2^/sr, signifying maximal retention at the injury site (Figure S15C). Over the subsequent days, the signal progressively declined, with a marked reduction by 72 h and further attenuation at 5 and 7 days, consistent with the expected NS-PTEN clearance dynamics. Across the 7 day postinjection period, the fluorescence signal demonstrated a dynamic pattern of NS-PTEN accumulation and clearance at the injury site.

To further evaluate the systemic pharmacokinetics and metabolic fate of NS-PTEN, we performed dynamic ex vivo fluorescence imaging of major organs following systemic administration (Figure S15B). Postinjection, NS-PTEN exhibited a time-dependent biodistribution pattern with preferential accumulation in the liver and spleen, consistent with the typical clearance route of NS-PTEN via the reticuloendothelial system. Quantitative analysis revealed that fluorescence intensity in the liver increased rapidly, reaching a maximum total radiant efficiency of ≈4.54 × 10^12^ p/s/cm^2^/sr at 24 h postinjection (Figure S15D). From 48 h onward, the signal intensity exhibited a progressive decline. By Day 7, the radiant efficiency had diminished substantially compared to the peak value, indicating that the NS-PTEN is effectively metabolized and cleared from the body rather than permanently retained.

At the cellular level, nuclear entry of NS-PTEN was detectable as early as DPI-1, predominantly within injured neurons at the lesion epicenter, whereas neurons in nonlesioned regions exhibited no discernible nuclear localization (Figure S16).^36,37^ By DPI-3, PTEN protein levels were markedly reduced in the NS-PTEN group (0.220 ± 0.025) compared with bare AuNP–treated animals (1.048 ± 0.039; *P* < 0.001), confirming efficient and rapid knockdown (Figure S17A,B). A longitudinal reconstruction of the spinal cord revealed that NS-PTEN transport was not restricted to the immediate lesion cavity. Instead, a gradient of fluorescence was observed extending cranially from the injury epicenter by 48 h postinjection. Validating the intracellular bioavailability, high-magnification analysis of randomized ROIs (No. 1–4) spanning the proximal and distal parenchyma confirmed the colocalization of NS-PTEN with neuronal somata (Figure 4H). It is well-established that suppressing PTEN can facilitate axon regeneration within the CNS; however, sustained suppression introduces a potential risk of tumor development.^38,39^ Accordingly, a return toward physiological PTEN levels is desirable once regenerative programs have been initiated. PTEN suppression progressively waned over time, and by DPI-28, only a partial reduction remained (*P* < 0.01 versus controls), aligning with the expected temporal window of NS-PTEN-mediated gene silencing.

### 2.6 Targeted Intraspinal Delivery of NS-PTEN Improves Motor Outcomes After SCI

To determine whether early PTEN knockdown translates into functional benefits after SCI, we performed a multimodal behavioral assessment encompassing open-field locomotion, mechanical nociception, neuromuscular coordination, quantitative gait kinematics, and gastrocnemius muscle preservation. Functional consequences of NS-PTEN treatment were first evident in gross hindlimb morphology and footprint gait patterns (Figure 4C). At DPI-28, Sham animals displayed coordinated plantar stepping and symmetric hindlimb posture, whereas untreated Injury animals exhibited pronounced paw dragging, toe curling, and irregular limb placement. AuNP-treated rats showed modest improvement but retained a substantial gait instability. In contrast, NS-PTEN–treated animals demonstrated markedly improved hindlimb extension and paw contact with footprint trajectories revealing more continuous, alternating step patterns. Ink-based footprint tracking further highlighted restoration of bilateral coordination in the NS-PTEN group, with clearer stride definition and reduced compensatory widening compared with those of the Injury and AuNP groups.

Across all domains, NS–PTEN consistently produced superior recovery compared to both untreated injury and AuNP carriers. Quantitative gait analysis revealed marked impairments in both stride width and stride length after SCI. Stride width was significantly increased in the Injury group (42.5 ± 3.69 mm), whereas NS-PTEN rats showed a substantially narrower and more normalized width (24.5 ± 2.42 mm), falling between AuNP (35.3 ± 3.34 mm) and Sham controls (20.36 ± 2.11 mm) (Figure 4D). Stride length showed an even stronger separation, and injured animals exhibited a complete loss of effective stride, while NS-PTEN treatment restored substantial step length (56.77 ± 5.51 mm), markedly exceeding the AuNP group (43.94 ± 8.34 mm) and approaching Sham values (65.9 ± 5.17 mm). Consistent with improved limb use, gastrocnemius muscle weight was profoundly reduced in Injured rats (0.744 ± 0.109 g) but partially preserved in NS-PTEN–treated animals (1.283 ± 0.073 g), outperforming the AuNP group (0.855 ± 0.071 g).

Behavioral assessments across locomotor, coordination, and sensory domains revealed a coherent treatment-dependent profile following SCI. Mechanical sensitivity assessed by the VFT shows NS-PTEN significantly increased withdrawal thresholds compared with Injury (+1.93 g, *P* = 0.0045), reducing chronic hypersensitivity, whereas AuNP again provided no improvement at DPI-28 (Figure 4E). Locomotor recovery quantified by BBB scoring showed that NS-PTEN produced a robust and progressive improvement beginning at DPI 7, with significantly higher scores than untreated injury animals at every subsequent time point (DPI 7: + 4.47, *P* < 0.0001; DPI 14: + 7.06, *P* = 0.0005; DPI 21: + 8.42, *P* < 0.0001; DPI-28: + 8.39, *P* < 0.0001) (Figure 4F). In contrast, AuNP alone did not improve BBB scores relative to Injury across early and midphase recovery and demonstrated only modest late-stage separation at DPI 21 and DPI 28 (*P* = 0.048 and *P* = 0.0083, respectively). Direct comparisons confirmed that NS-PTEN consistently outperformed AuNP from DPI 14 onward, establishing a superior locomotor recovery trajectory. Furthermore, the Rotarod test did not reveal a statistically significant difference between the treatment and control groups by DPI-28 (Figure 4G). Unlike the Injury and AuNP groups, the NS-PTEN group displayed a progressive upward trajectory during the chronic phase. This may become statistically distinguishable over a longer therapeutic timeline.

For further testing of the quality of hindlimb posture and stepping, we implemented markerless pose estimation using DeepLabCut (version 2.7). We analyzed the vertical excursion of the hindlimb to assess the quality of gait recovery (Figure S20A,B). Rats in the Sham group exhibited normal, consistent stepping patterns with a high degree of ankle clearance. The mean hindlimb swing amplitude was 2.37 ± 0.34 cm (Figure S20C). Following SCI, the injury group showed severe locomotor deficits, characterized by dorsal stepping and dragging of the hindlimb. The vertical swing amplitude was significantly reduced to 0.46 ± 0.05 cm, representing only 19% of the Sham baseline. Treatment with AuNP resulted in partial and variable recovery. While some steps showed improved clearance, others remained close to the dragging baseline. The mean amplitude was 1.23 ± 0.53 cm, indicating a transitional phase of recovery with observed instability. The NS-PTEN-treated group demonstrated the most robust and stable functional recovery among the injury groups. These animals consistently displayed coordinated plantar stepping with significant weight support. The mean hindlimb swing amplitude reached 1.47 ± 0.20 cm, which was significantly higher than that of the Injury group and showed reduced variability compared to the AuNP group, suggesting a superior therapeutic effect on locomotor kinematics.

We next sought to validate the functional efficacy of NS-PTEN-mediated neuroregeneration by examining spontaneous locomotion and exploratory patterns via the OFT at 28 dpi. The NS-PTEN–treated group exhibited a clear improvement in overall mobility compared with controls (Figure S20D,E). Qualitative analysis of the motion heatmaps and tracking trajectories revealed distinct behavioral patterns across groups. Sham animals exhibited extensive exploration covering both the peripheral and central zones, whereas the Injury group displayed limited mobility, characterized by thigmotaxis by wall-hugging and confined movement restricted to the arena corners. In contrast, rats treated with NS-PTEN demonstrated significantly expanded exploration areas and continuous movement trajectories, closely resembling the behavioral phenotype of healthy controls. Quantification of locomotor metrics confirmed these observations. Across multiple open-field behavioral metrics, NS-PTEN treatment produced a robust recovery of locomotor performance after SCI (Figure S20D,E). Total distance traveled increased to 55.04 ± 3.78 m in the NS-PTEN group, significantly higher than the 25.61 ± 0.83 m observed in the Injury group (*P* < 0.0001) and approaching the performance of Sham animals (64.45 ± 3.65 m) (Figure S20F). Consistently, mean locomotor speed improved dramatically, rising from 42.68 ± 1.38 mm/s in the Injury group to 91.73 ± 6.30 mm/s following NS-PTEN treatment (*P* < 0.0001), nearly doubling the impaired animals’ gait velocity. We next assessed vertical exploration, which requires hindlimb stability and trunk control. Free rearing time, a sensitive measure of weight-bearing capacity, was profoundly impaired after SCI (5.24 ± 2.93 s). NS-PTEN restored this complex motor behavior to 20.63 ± 3.84 s, a significant improvement (*P* = 0.001). Also, the NS-PTEN group maintained a low resting duration and trending lower than Injury, consistent with sustained engagement in active locomotion.

Finally, to assess the electrophysiological integrity of the corticospinal tract following SCI, we recorded motor-evoked potentials (MEPs) from the tibialis anterior muscle by stimulating the primary motor cortex (Figure S21A). Consistent with a complete conduction block, rats subjected to SCI exhibited an absence of detectable MEP responses, presenting only baseline noise with no stimulus-locked activity (Figure S20B). In contrast, Sham-operated animals displayed robust and reproducible MEP waveforms with clearly identifiable peaks, confirming intact corticospinal connectivity. Notably, the administration of NS-PTEN markedly restored the presence of stimulus-evoked responses. While animals treated with AuNPs alone showed no appreciable restoration and remained electrophysiologically comparable to the SCI group, NS-PTEN–treated rats exhibited discernible MEP peaks with amplitudes significantly exceeding background noise levels. High-resolution insets highlight several stimulus-associated waveforms in the NS-PTEN group, suggesting a partial reactivation of functional corticospinal conduction. Together, these multimodal assessments demonstrate that targeted intraspinal delivery of NS-PTEN restores motor function across multiple behavioral and anatomical measures following spinal cord injury.

### 2.7 NS-PTEN Reprograms Intracellular Signaling to Orchestrate a Pro-regenerative Microenvironment

To elucidate the molecular mechanism underlying the observed functional recovery, we investigated whether NS-PTEN could effectively reverse the intrinsic growth inhibition characteristic of CNS injury (Figure 5A). We hypothesized that silencing PTEN would activate the PI3K/AKT pathway, thereby triggering a cascade of downstream effects essential for neuronal survival and plasticity. Immunoblotting of the spinal cord lesion epicenter at 4 days postinjury confirmed precise engagement of the molecular target (Figure 5B,C): NS-PTEN treatment significantly reduced PTEN protein expression to approximately 25% of levels observed in the Injury group (Injury: 1.12 ± 0.17 vs NS-PTEN: 0.28 ± 0.12; *P* < 0.0001). This knockdown successfully relieved the inhibition of the PI3K/AKT axis, evidenced by a robust resurgence in AKT phosphorylation (Injury: 1.02 ± 0.14 vs NS-PTEN: 1.82 ± 0.28; *P* < 0.01). Consequently, downstream anabolic signaling was restored; phosphorylation of mTOR, a critical regulator of protein synthesis and axon growth, was significantly upregulated compared to the injury control (Injury: 0.57 ± 0.10 vs NS-PTEN: 1.68 ± 0.23; *P* < 0.0001). Furthermore, NS-PTEN treatment distinctly modulated GSK-3β phosphorylation levels (Injury: 1.44 ± 0.27 vs NS-PTEN: 0.67 ± 0.18; *P* < 0.01), suggesting a coordinated regulation of cytoskeletal stabilization and pro-regenerative transcription.

**Figure 5:**
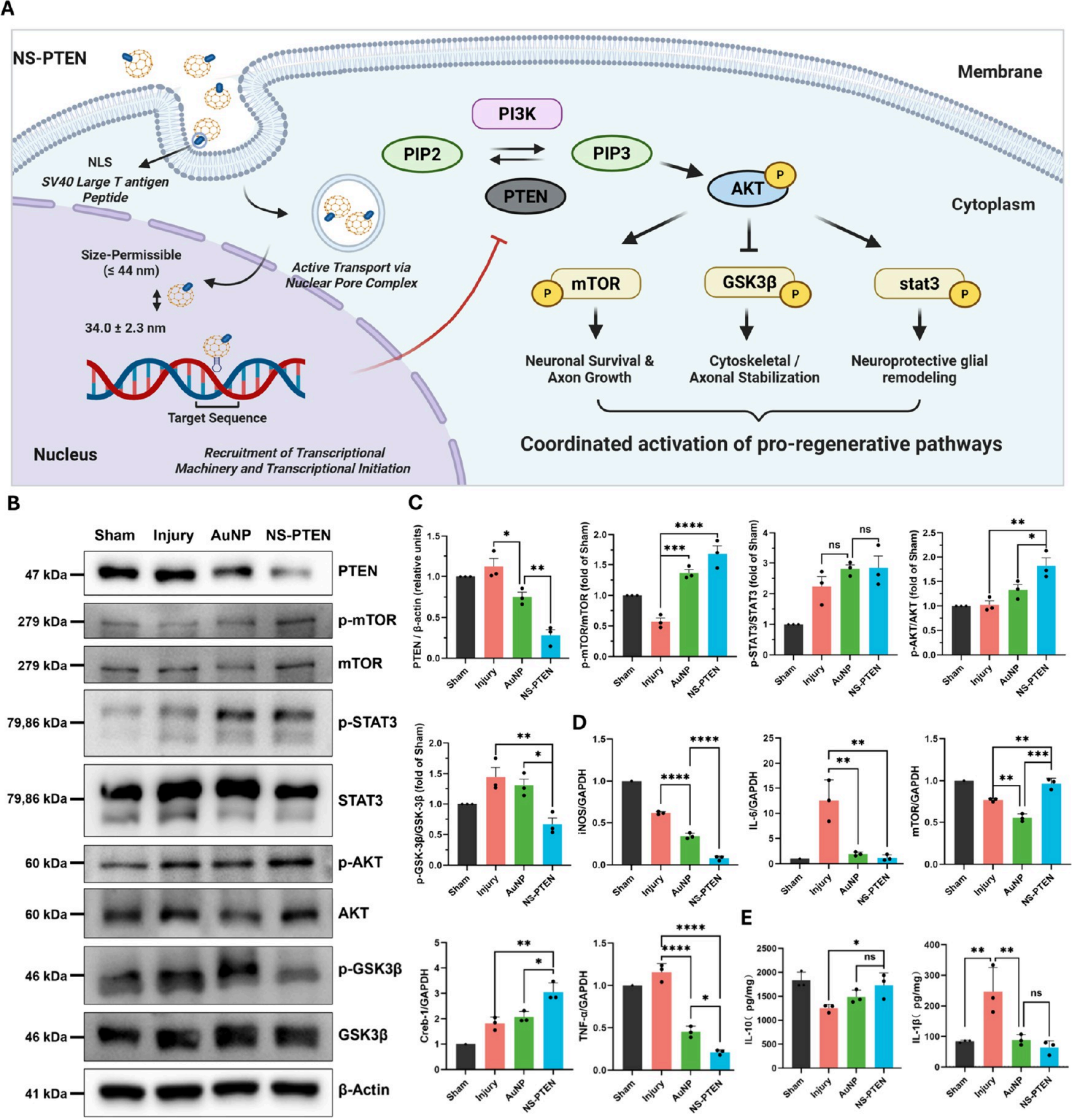
NS-PTEN reprograms intracellular signaling and remodels the immune microenvironment at early start post-SCI injury. (A) Schematic illustration of the proposed molecular mechanism. NS-PTEN enters the nucleus via the nuclear pore complex to silence PTEN expression, thereby relieving the inhibition on the PI3K/AKT pathway and activating downstream effectors to coordinate neuronal survival and regeneration. (B) Representative Western blot images showing the expression of PTEN and phosphorylation levels of key signaling proteins in the spinal cord lesion epicenter. (C) Densitometric quantification of Western blot data. (D) RT-qPCR analysis of transcriptional changes. (E) Quantification of inflammatory cytokine concentrations via ELISA. Statistical significance was determined using ANOVA with Tukey’s test. Data are presented as mean ± SD. Statistical significance was determined using ANOVA followed by Tukey’s post hoc test. *P < 0.05, **P < 0.01, ***P < 0.001, ****P < 0.0001; ns, not significant.

For better determination of whether the reactivation of intracellular signaling translates into a favorable microenvironment for regeneration, we also profiled the transcriptional landscape of the injury area (Figure 5D). RT-qPCR analysis revealed that NS-PTEN treatment successfully orchestrated a dual-pronged response: reversing the injury-induced suppression of neuroprotective genes while concurrently extinguishing the inflammatory surge. Consistent with the restoration of the PI3K/AKT axis, NS-PTEN significantly rescued the mRNA expression of mTOR to near-baseline levels (Injury: 0.77 ± 0.02 vs NS-PTEN: 0.96 ± 0.07; *P* < 0.01). Moreover, we observed the potential of Creb-1, a master transcription factor for neuronal plasticity. While injury alone elicited a modest stress-induced increase in Creb-1, NS-PTEN treatment further amplified this expression by approximately 1.7-fold relative to the vehicle control (Injury: 1.82 ± 0.25 vs NS-PTEN: 3.05 ± 0.36; *P* < 0.01). In parallel, NS-PTEN exerted a profound immunomodulatory effect. The injury microenvironment, characterized by a cytokine storm, showed marked downregulation of key pro-inflammatory mediators following treatment. Strikingly, NS-PTEN administration abrogated IL-6 expression, reducing levels by over 90% (Injury: 12.55 ± 4.14 vs NS-PTEN: 1.18 ± 0.60; *P* < 0.01). This anti-inflammatory profile was further corroborated by the suppression of TNF-α (Injury: 1.16 ± 0.10 vs NS-PTEN: 0.21 ± 0.03; *P* < 0.0001) and the drastic reduction of the oxidative stress marker iNOS (Injury: 0.62 ± 0.02 vs NS-PTEN: 0.08 ± 0.03; *P* < 0.0001), indicating a comprehensive mitigation of the neurotoxic inflammatory cascade.

We next examined whether these transcriptional changes translated to functional differences in protein secretion by quantifying cytokine concentrations via ELISA (Figure 5E). Consistent with the mRNA profiles, NS-PTEN treatment potently attenuated the secretion of the key pro-inflammatory cytokine IL-1β, reducing concentrations to 63.56 ± 21.96 pg/mg, a nearly 4-fold decrease compared to the injury control (246.70 ± 78.84 pg/mg; *P* < 0.01). Concurrently, the treatment successfully rescued the injury-induced depletion of the anti-inflammatory cytokine IL-10. NS-PTEN restored IL-10 protein levels to 1732 ± 256 pg/mg, significantly elevating them above the untreated injury group (1251 ± 82 pg/mg; *P* < 0.05) and effectively returning them to near-baseline levels observed in sham animals. Collectively, these findings demonstrate that NS-PTEN orchestrates a comprehensive remodeling of the immune microenvironment, shifting the balance from a neurotoxic, pro-inflammatory state toward a pro-regenerative state.

### 2.8 NS-PTEN Preserves Spinal Cord Architecture and Promotes Neurogenesis after Injury

Gross morphological assessment at 28 dpi provided the first indication of therapeutic efficacy. Compared to the pronounced cavitation and tissue disruption observed in the untreated SCI cohort, animals treated with NS-PTEN demonstrated superior maintenance of spinal cord structural integrity and improved anatomical continuity. To quantify these observations and dissect the cellular dynamics of repair, we performed a systematic histological evaluation using H&E staining to measure lesion volume and LFB staining to assess myelination status within the white matter (Figure 6A–D). First, to ensure the validity of our comparative analysis, we confirmed the uniformity of the injury induction at 1 dpi. Quantitative analysis of both H&E and LFB staining revealed no significant differences in initial damage severity between the cohorts (*P* > 0.05). The NS-PTEN group exhibited a lesion area (20.815% ± 2.328) and demyelination extent (30.7% ± 1.773) comparable to those of the SCI group (lesion: 21.335% ± 1.094; demyelination: 31.12% ± 2.553), establishing a consistent baseline for therapeutic evaluation (Figure 6E,G). By 28 dpi, however, distinct regenerative outcomes emerged. NS-PTEN treatment significantly mitigated secondary tissue degeneration, resulting in a drastically reduced lesion area of 4.80% ± 0.70%, compared to the extensive damage (11.053% ± 0.856) persisting in the SCI group (*P* < 0.001) (Figure 6F,H). This enhanced tissue preservation was paralleled by robust white matter maintenance. LFB analysis demonstrated a significantly higher degree of myelination in NS-PTEN-treated rats (46.976% ± 1.323) relative to the SCI controls (37.681% ± 1.926, *P* < 0.001), suggesting that NS-PTEN effectively counters demyelination and promotes structural repair at the lesion epicenter.

**Figure 6:**
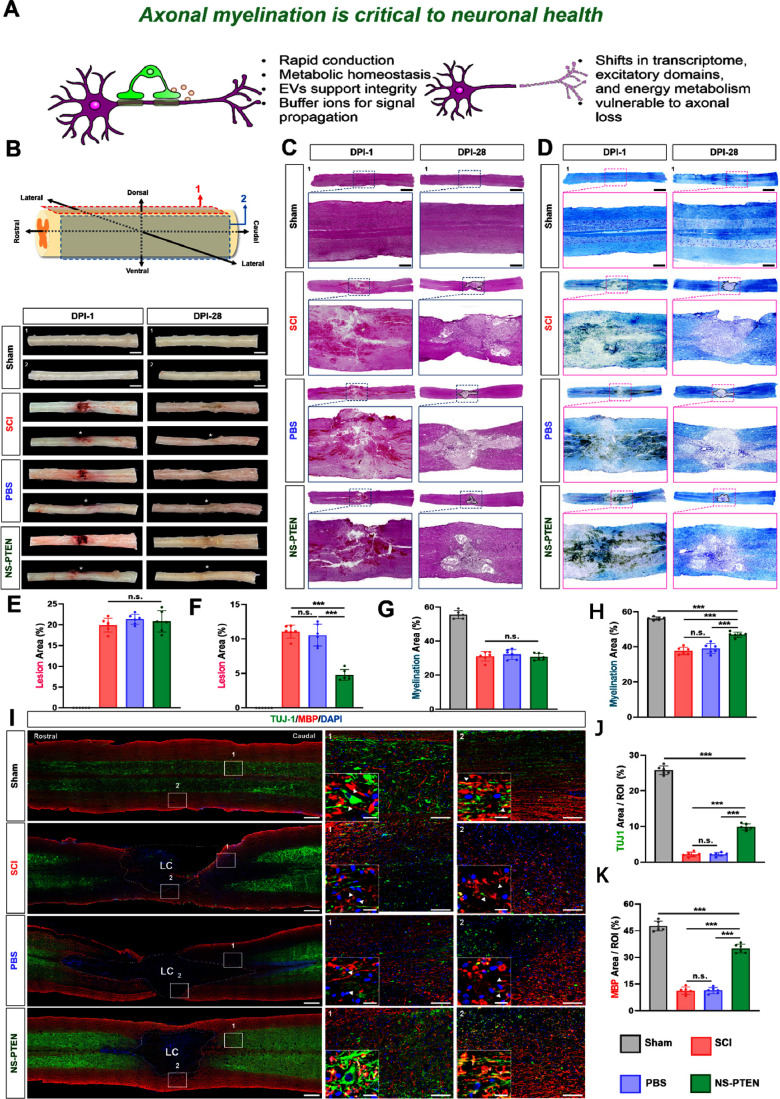
In vivo administration of NS-PTEN promotes neurogenesis and remyelination after SCI. (A) Experimental schematic of the spinal cord following SCI (B) Gross morphology of the spinal cord at 1 day postinjury (dpi) and 28 dpi. The asterisk marks the lesion epicenter. Scale bar, 3 mm. (C) Representative hematoxylin and eosin (H&E) staining of longitudinal spinal cord sections at 1 dpi and 28 dpi, illustrating lesion architecture and overall tissue integrity across experimental groups. Scale bar, 3 mm (500 μm in magnified regions). (D) Representative Luxol Fast Blue (LFB) staining at 1 and 28 dpi, showing white matter integrity and myelination status within and surrounding the lesion site. Scale bar, 3 mm (500 μm in magnified regions). (E, F) Quantification of lesion area based on H&E staining, expressed as lesion area relative to total tissue area at (E) 1 dpi and (F) 28 dpi. (G and H) Quantification of white matter area based on LFB staining, expressed as myelinated area relative to total tissue area at (G) 1 dpi and (H) 28 dpi. (I) Representative longitudinal immunofluorescence images of spinal cord sections at 28 dpi. Neurons are labeled with TUJ1 (green), myelin sheaths with myelin basic protein (MBP, red), and nuclei with DAPI (blue). The white dashed outline indicates the lesion area surrounding the LC. Scale bar: 500 μm. (J) Percentage of the TUJ1-positive area within the lesion region. (K) Percentage of MBP-positive area within the lesion region. Data are presented as mean ± SD. Statistical significance was determined using ANOVA followed by Tukey’s post hoc test. *P < 0.05, **P < 0.01, ***P < 0.001, ****P < 0.0001; ns, not significant.

To further delineate the extent of neurogenesis and remyelination, we performed immunofluorescence staining at 28 days postinfection using TUJ1 (neuronal class III β-tubulin) and myelin basic protein (MBP), a key marker of myelination. Given the limited intrinsic capacity for neuronal regeneration post-SCI, assessing these markers was pivotal for evaluating the therapeutic efficacy of NS-PTEN (Figure 6I).^40–43^ High-magnification imaging revealed striking morphological preservation in the treated animals. The neuronal cell body phenotype in the NS-PTEN group exhibited an organized structure closely resembling that of the Sham group. Furthermore, we observed a distinct overlapping and colocalization of axons and myelin sheaths in the NS-PTEN group, which stood in sharp contrast to the fragmented axons and disjointed myelin structure characteristic of the SCI group. Quantitative analysis corroborated these histological observations, confirming that NS-PTEN significantly enhanced early neuronal differentiation and preservation. The expression of TUJ1 in the NS-PTEN group reached 9.867% ± 0.765%, a substantial increase compared to the minimal expression observed in the SCI group (2.171% ± 0.472%; *P* < 0.001) (Figure 6J). While neuronal density did not fully recover to baseline Sham levels, the treatment effect represented a nearly 5-fold improvement over the injury control. Parallel improvements were seen in myelination, which is essential for facilitating efficient signal transmission. The NS-PTEN group demonstrated a robust restoration of the myelin sheath, with MBP expression rising to 35.012% ± 2.27%, significantly exceeding the 11.242% ± 1.884% recorded in the SCI group (*P* < 0.001) (Figure 6K). Collectively, these results demonstrate that NS-PTEN stimulates axonal regrowth and functional network re-establishment via dynamic remyelination, effectively overcoming the dual barriers of neuronal loss and demyelination inherent to SCI pathology.

### 2.9 NS-PTEN Enhances Axonal Integrity, Remyelination, and Regenerative Sprouting after Spinal Cord Injury

To investigate the neuroprotective effects of NS-PTEN on spinal cord microarchitecture at 28 days postinjury, dual immunofluorescence staining for MBP and NF was performed (Figure 7A). The untreated injury group displayed severe tissue disruption characterized by cavitation, extensive loss of MBP-positive myelin, and disorganized, fragmented NF-labeled axons, particularly within the central lesion core. Surrounding the lesion site, sparse remyelination and irregular axonal sprouting were observed, consistent with chronic demyelination and the formation of a glial scar. SD Rats treated with AuNPs demonstrated a moderate preservation of tissue structure, with a partial restoration of the MBP signal and a regional increase in NF labeling within the peri-lesional areas. However, the spatial distributions of both markers remained discontinuous, suggesting that the regenerative benefit of AuNP treatment alone was limited. Strikingly, the NS-PTEN-treated animals displayed markedly improved preservation of the spinal cord architecture. High-magnification views revealed a significantly denser and more continuous MBP signal spanning across and beyond the lesion interface. This was accompanied by well-aligned, elongated NF-positive axonal fibers extending toward the injury core. These morphological features are strongly suggestive of concurrent remyelination and axonal elongation. Furthermore, the peri-lesional zones in the NS-PTEN group exhibited reduced vacuolation and a more compact cellular arrangement, indicating an attenuation of secondary degeneration. Spatial fluorescence profiling for MBP and NF, as shown in the spatial intensity profile analysis, corroborated these histological observations (Figure 7B). The NS-PTEN treatment resulted in a significantly elevated MBP signal intensity compared with both the AuNP and untreated Injury groups. Similarly, the NF intensity was highest in the NS-PTEN group, exhibiting a continuous distribution pattern that is suggestive of improved anatomical connectivity.

**Figure 7:**
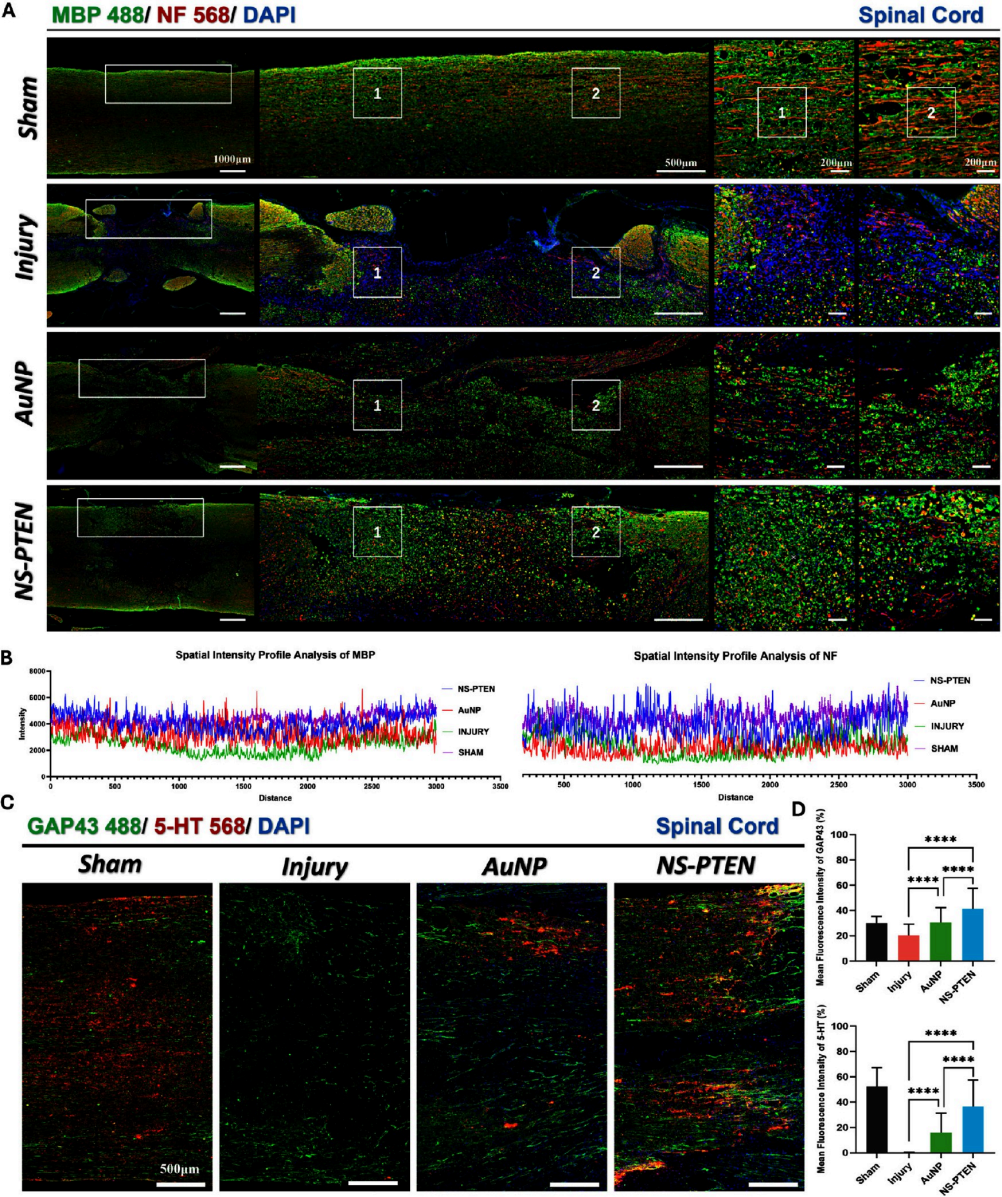
NS-PTEN treatment preserves myelin structure and promotes axonal regeneration after spinal cord injury. (A) Representative longitudinal immunofluorescence images of the spinal cord lesion site stained for Myelin Basic Protein (MBP, green) and Neurofilament (NF, red). Nuclei were counterstained with DAPI (blue). The white boxed areas (1 and 2) indicate the regions shown at higher magnification on the right. The NS-PTEN group exhibits preserved myelin architecture and axonal continuity compared with the fragmented tissue observed in the Injury and AuNP groups. Scale bars: 1000 μm, 500 μm, and 200 μm. (B) Spatial intensity profile analysis quantifying the fluorescence signal distribution of MBP and NF across the lesion epicenter, demonstrating higher signal integrity in the NS-PTEN group. (C) Representative immunofluorescence staining for the growth-associated protein GAP43 (green) and serotonergic fibers (5-HT, red) in the spinal cord lesion epicenter. Scale bar: 500 μm. (D) Quantification of the mean fluorescence intensity (%) for GAP43 and 5-HT. NS-PTEN treatment significantly enhanced the expression of regeneration markers compared to the Injury control. Data are presented as mean ± SD. Statistical significance was determined using ANOVA followed by Tukey’s post hoc test. *P < 0.05, **P < 0.01, ***P < 0.001, ****P < 0.0001; ns, not significant.

To determine whether NS-PTEN enhances axonal regeneration and serotonergic reinnervation at 28 days postinjury, we performed dual immunofluorescence staining for GAP43 and 5-HT (Figure 7C). In the untreated Injury group, both regenerative GAP43^+^ and serotonergic 5-HT^+^ fibers were profoundly diminished, appearing sparse, fragmented, and largely confined to the lesion margins, hallmarks of severely impaired axon growth and disrupted descending monoaminergic input. Quantitatively, the Injury group showed a dramatic reduction in 5-HT intensity (0.21 ± 0.27) and a similarly low GAP43 signal (20.26 ± 8.99), consistent with widespread degeneration of descending projections and regenerative failure (Figure 7D). NS-PTEN treatment produced a markedly different pattern. The injured spinal cords displayed dense, longitudinally aligned GAP43^+^ fibers traversing the lesion core, accompanied by robust 5-HT^+^ axons extending into previously denervated regions. Mean fluorescence intensity analysis confirmed that NS-PTEN significantly increased both GAP43 (41.44 ± 16.19) and 5-HT (36.76 ± 20.59) signals, outperforming both the Injury and AuNP groups (*P* < 0.0001). In contrast, AuNP carriers produced only modest, incomplete improvements (GAP43:30.65 ± 11.62; 5-HT: 15.97 ± 15.46), indicating that AuNPs alone do not meaningfully support axonal regeneration. These findings establish that NS-PTEN simultaneously preserves postinjury axonal viability and promotes regenerative axonal growth—dual mechanisms that converge to produce superior anatomical and functional restoration, with potential implications for clinical spinal cord injury treatment.

### 2.10 NS-PTEN Remodels the Inflammatory Microenvironment to Promote Neuroprotection and Restore Blood-Spinal Cord Barrier Integrity

To evaluate the therapeutic potential of NS-PTEN in SCI recovery, we investigated its ability to modulate the critical balance between neuroinflammation and the secondary neuroprotective response. Immunofluorescence imaging revealed striking differences in the inflammatory architecture of the injured spinal cord across treatment groups. Reactive astrocytes play a dual role in SCI. During the acute phase, they facilitate tissue repair by stabilizing the injury site and mitigating secondary damage. However, in the chronic phase, these astrocytes transition into a scar-forming phenotype, depositing dense glial scars that create a physical and biochemical barrier to axonal regeneration.^10,44^ To dissect the structural characteristics of the glial scar, we focused our quantitative analysis on the spatial distribution of GFAP, specifically at the lesion margins. Visualizing this morphology through immunofluorescence allowed us to distinguish between the necessary astrocytic response and the formation of an inhibitory physical barrier. While a generalized upregulation of GFAP was evident within the injury zone across all groups, reflecting the natural recruitment of astrocytes to the trauma site, the architecture at the lesion border presented a critical divergence (Figure 8A). GFAP staining revealed prominent, hypertrophic astrocytes forming a dense glial border around the lesion in both Injury and AuNP groups, whereas NS-PTEN treatment substantially attenuated this reactive gliosis, consistent with its reduced GFAP intensity (1.883 ± 0.12 vs 2.347 ± 0.32 in Injury) (Figure 8C). Likewise, Iba1 staining demonstrated extensive microglial activation in the Injury and especially the AuNP group, which exhibited the highest Iba1 signal intensity. NS-PTEN markedly reduced this activation (1.378 ± 0.13), which was lower than both the Injury and AuNP groups (*P* < 0.0001). Furthermore, immunofluorescence of NF in magnified images near the lesion center confirmed enhanced axon regeneration in the NS-PTEN group at varying distances from the lesion site (Figure 8B,C). Upon examining the data obtained from our investigation, we found that the density of axons was lower in the regions that were closer to the lesion’s core. Immunofluorescence staining for NF, visualized through both 1D and 2.5D spatial reconstructions, confirmed that NS-PTEN treatment significantly preserved axonal integrity, axon regeneration was significantly more in the NS-PTEN group compared to the SCI, both at −0.5 mm [NS-PTEN (15.319% ± 2.992) vs SCI (4.407% ± 1.138)] and 0.5 mm [NS-PTEN (17.765% ± 6.202) vs SCI (4.612% ± 0.759)] from the lesion site (*P* < 0.001).

**Figure 8:**
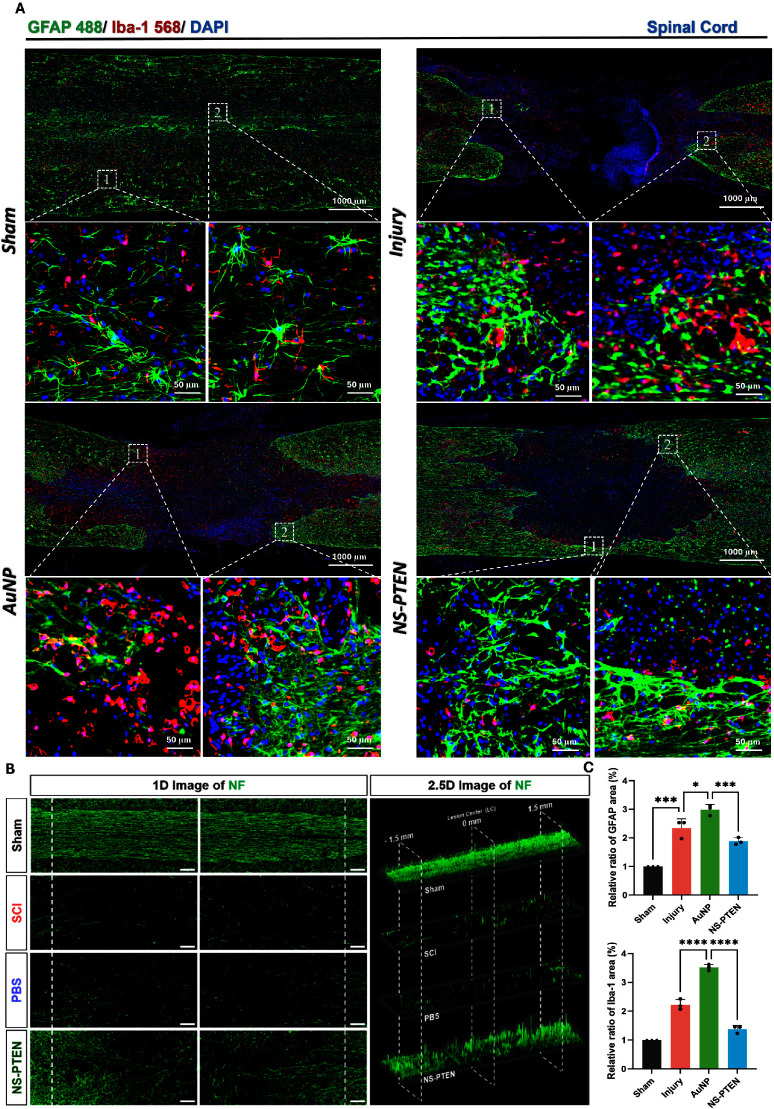
NS-PTEN mitigates neuroinflammation, preserves neural architecture, promotes M2 macrophage polarization, and reduces apoptosis after spinal cord injury. (A) Representative immunofluorescence images of spinal cord sections stained for GFAP (green), Iba1 (red), and DAPI (blue), illustrating astrocytic reactivity and microglial activation across experimental groups. (B) Longitudinal NF staining depicting axonal continuity and structural preservation. One-dimensional NF projections reveal pronounced axonal fragmentation in the SCI group, whereas NS-PTEN maintains longitudinal fiber alignment. (C) Quantitative analysis of GFAP-positive and Iba1-positive areas. (D) Representative immunofluorescence staining for CD68 (green, pan-macrophage/microglia marker) and CD163 (red, M2 anti-inflammatory macrophage marker), with DAPI counterstaining (blue). (E) TUNEL staining (green) showing apoptotic cells within the lesion area with nuclei labeled by DAPI (blue). Scale bars: 100 and 20 μm. (F) Quantification of CD163^+^ cell percentage per field (top) and TUNEL^+^ apoptotic cell percentage (bottom). Statistical significance was determined using one-way ANOVA with Tukey’s post hoc test. Data are presented as mean ± SD. Statistical significance was determined using ANOVA followed by Tukey’s post hoc test. *P < 0.05, **P < 0.01, ***P < 0.001, ****P < 0.0001; ns, not significant.

In the Injury and AuNP groups, dense clusters of CD68^+^ activated macrophages dominated the lesion core, accompanied by scattered CD163^+^ cells, indicative of a strongly pro-inflammatory M1-biased response (Figure 8D,F). In contrast, NS-PTEN treatment produced a markedly altered pattern with reduced CD68^+^ infiltration and a visibly higher abundance of CD163^+^ cells, suggesting a phenotypic shift toward reparative macrophage programming. Quantitative analysis confirmed this observation that the CD163 positive ratio increased dramatically from 15.06 ± 1.64% in Injury to 45.90 ± 3.65% in the NS-PTEN group (*P* < 0.0001), representing a 3-fold enhancement of M2-like polarization, far exceeding the partial increase seen in AuNP controls (25.76 ± 4.88%). The cell death patterns also mirrored these inflammatory trends that TUNEL staining revealed widespread apoptosis throughout the lesion in the Injured spinal cord (18.05 ± 1.27%), with AuNP providing modest attenuation (12.04 ± 0.50%) (Figure 8E,F). NS-PTEN treatment produced a much more pronounced reduction in apoptotic cells (7.41 ± 1.38%, *P* < 0.0001), consistent with the visual impression of sparse TUNEL^+^ nuclei in the IF images. This reduction suggests that early PTEN knockdown confers substantial neuroprotection, likely reflecting decreased inflammatory toxicity and improved microenvironmental stability.

To investigate whether this profound immune modulation translates into a secondary neuroprotective response, we examined the downstream effects of the altered microenvironment on the neurotrophic support and vascular integrity. We hypothesized that by suppressing the neurotoxic activity of microglia and reducing the physical barrier of the glial scar, NS-PTEN creates a permissive niche that indirectly favors endogenous repair mechanisms. We first focused on the interplay between Iba1, a marker of activated microglia, and brain-derived neurotrophic factor (BDNF), which is essential for neuronal survival and regeneration. We hypothesized that NS-PTEN could rebalance the injury microenvironment toward a pro-regenerative state (Figure S18A,D).^45^ Our results demonstrated that NS-PTEN treatment robustly suppressed injury-associated neuroinflammation, as evidenced by a marked reduction in microglial activation. Specifically, the microglial-positive area in the NS-PTEN group was reduced to 6.448% ± 0.847, approaching levels observed in the Sham group (3.203% ± 0.106) with no statistically significant difference between the two groups (*P* > 0.05) (Figure S18B,E). Concomitant with this attenuation of inflammation, NS-PTEN significantly enhanced the neurotrophic support. BDNF expression in the NS-PTEN group increased to 24.348% ± 1.787, representing a more than 2-fold elevation compared with the SCI group (Figure S18B,F). These findings indicate that NS-PTEN orchestrates a dual modulatory effect at the injury site, simultaneously dampening microglia-mediated inflammatory responses and amplifying BDNF-dependent neurotrophic signaling. The combination of these two capabilities might promote neuronal survival and axon regeneration, which could provide a path to recovering from spinal cord injuries.

While PTEN inhibition is also known to promote angiogenesis via the PI3K/Akt/VEGF axis directly, our data emphasize the restoration of a permissive microenvironment as a prerequisite for this structural recovery. Then, we examined the restoration of the blood-spinal cord barrier (BSCB), a structure crucial for maintaining spinal cord homeostasis. Since BSCB disruption exacerbates pathology by facilitating inflammatory infiltration, its repair is a prerequisite for neuroregeneration. We specifically evaluated the expression of CD31, a marker of endothelial cells indicating angiogenesis, and Occludin, a tight junction protein essential for barrier integrity.^46–48^ Our observations align with this understanding, suggesting that in the SCI and PBS groups, where BSCB restoration was less evident, there was a corresponding increase in inflammation and reduced regenerative potential. Conversely, in the NS-PTEN group, where BSCB restoration was more robustly achieved, we observed reduced inflammation and an enhanced environment for neurogenesis. Given the importance of BSCB integrity post-SCI, we focused on the expression of CD31 and occludin, markers indicative of endothelial cells comprising the BSCB and tight junctions that regulate solute movement, respectively (Figure S18C).^49,50^ Our results showed a significant increase (*P* < 0.001) in CD31 expression in the NS-PTEN group (2.337% ± 0.439), compared to the SCI (0.711% ± 0.083) and PBS (0.782% ± 0.139) groups. Similarly, occludin expression was notably higher in the NS-PTEN group (1.895% ± 0.392) compared to the SCI (0.518% ± 0.07) and PBS (0.55% ± 0.089) groups (Figure S18G,H).

These results indicate that NS-PTEN reshapes the postinjury spinal cord microenvironment by suppressing excessive neuroinflammation and modulating glial scar architecture at the lesion margin. This immune reprogramming is accompanied by reduced apoptosis, enhanced axonal preservation and regeneration, increased neurotrophic support, and restoration of blood–spinal cord barrier integrity. Together, these coordinated effects establish a permissive niche that supports endogenous repair processes and contributes to functional recovery after SCI.

### 2.11 NS-PTEN Facilitates Glial Bridging via an Immunologically “Cold” Microenvironment

An intriguing finding in our study was the upregulation of STAT3 in the NS-PTEN group, a pathway canonically associated with astrogliosis. While excessive glial scarring is often viewed as a physical barrier to regeneration, complete ablation of the astrocytic response can lead to failure of wound closure and extensive tissue degeneration.^51,52^ Morphologically, we observed that GFAP+ astrocytes in the treated group adopted a linear, laminar organization paralleling the longitudinal axis of the spinal cord, contrasting sharply with the chaotic, mesh-like barrier seen in untreated controls. This “glial bridging” effect is critical for providing topographic guidance for regenerating axons. This structural permissiveness is strongly supported by our histological analysis of axonal integrity.^53^ The spatial intensity profile analysis revealed a promising preservation of the NF density and MBP architecture within the lesion core. A process that would be severely compromised in the presence of a dense, inhibitory scar.^54^

Crucially, this astrocytic activation occurred within an immunologically “cold” microenvironment, distinct from the chronic inflammatory milieu that typically drives maladaptive scarring. As evidenced by our qPCR analysis, the NS-PTEN treatment significantly attenuated the expression of iNOS, which is a hallmark of pro-inflammatory M1 microglia and key inflammatory cytokines, including IL-6 and TNF-α, which are known inducers of the neurotoxic astrocyte phenotype.^55,56^ This anti-inflammatory shift was further corroborated by ELISA analysis of the CSF, where we observed a significant suppression of IL-1β alongside the preservation of the anti-inflammatory cytokine IL-10. By abrogation of these upstream inflammatory drivers, our treatment effectively decouples STAT3 signaling from neurotoxicity. Consequently, the upregulated STAT3 likely drives a reparative “A2-like” program, facilitating wound closure and structural support, rather than propagating the inflammatory cascade. Collectively, these findings establish that NS-PTEN promotes neural recovery after spinal cord injury through dual synergistic mechanisms: transient localized PTEN inhibition promotes axonal regeneration and suppresses glial scarring. Critically, this spatiotemporal specificity—achieved through nanoparticle targeting and transient drug release—minimizes off-target effects and distinguishes NS-PTEN from uncontrolled PTEN suppression strategies, offering a more selective therapeutic approach.

Despite these promising findings, a limitation of the current study is the noncell-type-specific nature of the NS-PTEN delivery system. While we demonstrate a net positive effect on the lesion microenvironment, we could not strictly isolate the intrinsic neuronal effects from the extrinsic astrocytic modulation. The intricate balance between beneficial wound closure and detrimental scarring is regulated by complex signaling networks. In our future investigations, we aim to refine this therapeutic strategy by employing dual-targeting approaches. Specifically, we plan to combine neuron-specific PTEN knockdown to drive intrinsic regrowth with the targeted modulation of astrocyte secretion profiles to more precisely control extracellular matrix deposition and further optimize the glial scaffold for axonal crossing.

### 2.12 Systemic Toxicity Evaluation Demonstrates the Translational Safety Profile of NS-PTEN

To ensure the translational potential of NS-PTEN, we performed a comprehensive assessment of the systemic toxicity. Analysis of serum biochemistry and hematology in acute and chronic phases revealed that systemic administration of NS-PTEN elicited no detectable off-target toxicity. Hepatic and renal functional markers, as well as serum albumin levels, remained within physiological reference limits across all time points, indistinguishable from those of the Sham control group. Hematological profiling at 4 dpi highlighted a characteristic acute-phase response to SCI, evidenced by lymphopenia and granulocytosis (Figure S19A). Moreover, NS-PTEN treatment attenuated this inflammatory shift, preserving lymphocyte fractions relative to the untreated injury group. By 28 dpi, hematological parameters in all cohorts converged to baseline levels, ruling out chronic myelotoxicity (Figure S19B).

Corroborating these biochemical findings, histopathological examination of major organs via H&E staining at 28 dpi showed well-preserved tissue architecture (Figure S19C). We observed no signs of necrosis, congestion, or inflammatory infiltration. Specifically, cardiac tissues displayed orderly myofibers, hepatic sections exhibited intact lobular structures without steatosis, and renal morphology featured patent glomerular capsules. Collectively, these data confirm that NS-PTEN possesses a favorable biocompatibility profile, inducing neither acute systemic inflammation nor chronic organ pathology.

## 3 Conclusions

In this study, we successfully demonstrated the therapeutic potential of NS-PTEN to address the dual barriers of SCI: the inhibitory extrinsic microenvironment and the limited intrinsic regenerative capacity of adult neurons. Following CNS injury, axonal degeneration and neuronal apoptosis contribute to the hostile microenvironments marked by chronic inflammation, demyelination, and glial scar formation. While prior work has explored PTEN inhibition using viral vectors or shRNA to enhance axonal growth, these approaches face translational challenges, including immunogenicity, tumorigenic risks from permanent gene silencing, and inconsistent delivery efficiency.^57–61^ To overcome these hurdles, we developed NS-PTEN, a nonviral nanoparticle-based platform for transient and targeted PTEN suppression. In a contusion SCI rat model, NS-PTEN robustly activated the PI3K/AKT/mTOR pathway, reduced glial scar density, and promoted axonal regeneration, which correlated with significant functional recovery. Critically, NS-PTEN achieved these outcomes without the risks associated with viral integration or constitutive gene knockout, underscoring its clinical translatability.^62,63^ These findings establish NS-PTEN as a versatile and safe strategy to modulate gene expression for neural repair, offering a novel approach to addressing both extrinsic and intrinsic barriers to SCI recovery.

PTEN modulation via NS-PTEN promotes neuronal survival postinjury, enhances remyelination, and mitigates secondary injury while also addressing oncogenic concerns by ensuring transient suppression.^64–66^ Delivery through retrograde and antegrade routes enabled effective activation of regenerative pathways and axonal regrowth in microfluidic and in vivo models. NS-PTEN was validated in iPSC-NPC-derived neurons, where it suppressed PTEN and activated downstream Akt/PI3K/mTOR signaling, driving axonal growth. Functional assays in neurotoxin-injured neurons confirmed NS-PTEN’s efficacy, which was absent in AuNP controls. In vivo, retrograde delivery facilitated acute neuronal nuclear localization, and PTEN suppression (DPI-4) facilitated multifaceted recovery (DPI-28), including neurogenesis, remyelination, and inflammation reduction, culminating in improved behavioral outcomes. The transient nature of PTEN suppression was confirmed by its restoration at DPI-28, demonstrating the platform’s safety. NS-PTEN’s ability to modulate neuron-glia interactions further underscores its therapeutic potential, as PTEN suppression reduces pro-inflammatory TNF-α while increasing neuroprotective IL-10 in astrocytes.^67–69^ This modulation balances inflammatory resolution, axonal integrity, and remyelination, emphasizing PTEN’s pivotal role in SCI recovery.

Effective neuroprotection by retaining BSCB integrity and mitigating secondary injury is essential, as cellular infiltration and inflammation exacerbate SCI pathology. NS-PTEN stimulated BDNF secretion, reducing microglial activity and mitigating inflammation. Moreover, this platform enhanced endothelial cell and tight junction expression, promoting angiogenesis and counteracting the detrimental effects of a compromised BSCB. Another critical barrier to functional recovery and axon regeneration is the formation of the glial scar in the chronic phase post-SCI.^10^ At DPI-28, NS-PTEN significantly reduced the glial scar size, allowing axons to penetrate the lesion and regenerate effectively. Behavioral assays further validated these results, showing substantial motor function recovery in the NS-PTEN group, beginning at DPI-3 and becoming more pronounced by DPI-28. This positions NS-PTEN as a promising therapeutic approach compared with existing PTEN-inhibiting methods.

While NS-PTEN shows significant promise, several limitations warrant further investigation. Although PTEN suppression was transient, prolonged inhibition may pose risks of tumorigenesis. However, previous studies suggest that while PTEN loss is a critical step in tumorigenesis, additional genetic perturbations are typically required to induce aggressive cancers.^70–72^ Additionally, future work could focus on encapsulating NS-PTEN in lipid formulations designed to cross the BSCB, minimizing patient harm and enhancing translatability. Similarly, lipid compositions with neuron-targeting antibodies could further improve delivery precision and efficacy.^73,74^ Despite these limitations, NS-PTEN’s ability to transiently regulate gene expression opens avenues for combinatorial therapies targeting SCI’s multifactorial pathology, thereby advancing regenerative strategies for neurodegeneration, tissue repair, and personalized medicine.

In summary, this study pioneers the use of NS-PTEN for transient PTEN suppression, promoting neurogenesis, remyelination, and anti-inflammatory effects while mitigating risks associated with continuous PTEN inhibition. NS-PTEN’s compatibility with clinical models and its ability to address SCI’s multifactorial challenges underscore its translational promise. By enabling precise and transient gene regulation, NS-PTEN advances therapeutic tools for previously intractable conditions, offering a significant step forward in SCI recovery.

## 4 Experimental Section

### Study Design

In this study, we developed a biomimetic transcription factor (NS-PTEN) for the *in vivo* repression of PTEN in neurons following SCI to promote axonal regeneration and functional recovery. To evaluate the efficacy of this platform, we compared SCI rats treated with NS-PTEN to uninjured rats to determine their functional recovery via BBB locomotion score. We ensured that the procedures possessed statistical power for *in vitro* assays by having a minimum of three replicates within each condition to represent intragroup variations and determine statistical significance. Similarly, for all *in vivo* experiments, 11-week-old female Sprague–Dawley rats were utilized, and a minimum of 1 week was allowed to acclimate to their environment and alleviate stress. A minimum of three rats were included within each group, which enabled us to make statistical claims pertaining to the lesion area and biological phenomena occurring within the injury site. Western blots, reverse transcription-quantitative polymerase chain reaction, and immunohistochemistry were used to evaluate the expression of various genes and proteins that are pertinent to the Akt/PI3K/mTOR pathway. In contrast, Basso–Beattie–Bresnahan (BBB) scoring was used to evaluate the locomotion abilities of the rats within various groups.

### Animal Surgical Procedures and Care

All animal surgical and experimental procedures were performed in accordance with the guidelines approved by the Institutional Animal Care and Use Committee (IACUC230058) of the CHA University School of Medicine. Female Sprague–Dawley rats, aged 11 weeks and weighing 220–240 g, were procured from Koatech (Pyeongtaek, Korea). The rats were acclimatized for at least 1 week in animal breeding facilities maintained at 24 ± 3 °C and 55–65% humidity with a 12 h light/dark cycle to minimize stress. Prior to the surgery for animal modeling, rats were anesthetized via intraperitoneal injection using a mixture of zolazepam, tiletamine (Zoletil, 50 mg kg^–1^, Virbac Laboratories), and xylazine (Rompun, 10 mg/kg, Bayer) in a 1:1:1 ratio. The dorsal fur of anesthetized rats was shaved, and the skin was disinfected using povidone-iodine and 70% ethanol. A skin incision was made in the T9–11 region, and a laminectomy was performed at T10 to expose the spinal cord. A spinal contusion injury was then induced using a precise impactor device (RWD Life Science, code no. 68099 II); it specified parameters: a tip diameter of 3 mm, a dwell time of 5 s, a speed of 2 m s^–1^, and a depth of 2 mm (Figure S9 and Video S1). Postinjury, a 10 μL solution of either 1X PBS or NS-PTEN (4 mg mL^–1^) was injected using a 30G needle attached to a Hamilton syringe. The exceptions were the sham and SCI-only groups. The rats were divided into the following groups: sham (*n* = 15), SCI (*n* = 25), 1XPBS/AuNP (*n* = 25), and SCI + NS-PTEN (*n* = 35). After injection, the incision sites were sutured, and the rats were placed on a heating pad at 39 °C for recovery. Manual bladder expression was performed three times daily until spontaneous urination resumed. Postoperative care included daily subcutaneous administration of 0.9% saline and antibiotics (cefazolin, 15 mg kg^–1^, CKD Pharmaceuticals), as well as intramuscular analgesics (ketoprofen, 1 mg kg^–1^, SCD Pharmaceuticals) for 1 week.

### Administration of Thoracic Spinal Cord Injury and Intracranial Injection of NS-PTEN to Sprague–Dawley Rats

All experimental procedures were performed in compliance with animal protocols approved by the Institution Animal Care and Use Committee at Rutgers, State University of New Jersey.

To introduce contusion SCI to the thoracic spinal cord of animals, Sprague–Dawley rats at the age of 77 ± 1 day were chosen and anesthetized via isoflurane inhalation. A midline incision was made to expose the thoracic vertebrae. We performed a laminectomy to remove the whole lamina of T10 and half of T9 to make a 2.5 mm width rectangular exposure. We then used a MASCIS impactor (Model III, made by W.M. Keck Center of Collaborative Neuroscience, Rutgers University) to introduce impact to the spinal cord at a height of 25 mm, which resulted in a complete contusion SCI. The muscle layers were sutured, and the skin was secured with clips.

For the injection of NS-PTEN into the brains of the animals, the head of the Sprague–Dawley rats was fixed on a stereotaxic frame (Fine Science Tools, Inc.) to make the skull horizontal. A midline incision was made in the skull. Blunt dissection was performed to expose the skull. Around the bregma, a craniotomy provides access to the sensorimotor cortex in both hemispheres. Totally, 10 μL of either NS-PTEN or control nanoparticles were injected into the sensorimotor cortex in both hemispheres using a Hamilton micro syringe (Hamilton Company) attached by a fine glass pipet. Each hemisphere had 5 injection sites (coordinates from bregma in mm: anterior-posterior/medial-lateral/dorsal-ventral, 1.5/0.5/1.0, 1.5/2.5/1.0, 0.0/1.5/1.0, −1.5/.0.5/1.0, −1.5/2.5/1.0). The skin was secured by clips. The rats were placed on soft bedding on a warming pad held at 37 °C until fully awake. Urine was expressed by manually squeezing the bladder once daily until the rats regained bladder function.

### Corticospinal Tract Labeling

We labeled the CST of the animals through anterograde biotinylated dextran amine (BDA) tracing. We injected, in total 1.6 μL, of BDA-Texas Red (Vector Laboratories) into the sensorimotor cortex at four sites in each hemisphere (coordinates from bregma in mm: anterior-posterior/medial-lateral/dorsal-ventral, 1.5/1.5/1.0, 0.0/0.5/1.0, 0/2.5/1.0, −1.5/1.5/1.0, avoid the sites injected by NanoScript). Two weeks after the injection of BDA, the rats were sacrificed for analysis.

### Preparation for Histology and Immunohistochemistry

Rats were sacrificed by giving a lethal dose of anesthesia and transcardially perfused with 4% paraformaldehyde. Brains and spinal cords were collected and postfixed in 4% paraformaldehyde overnight at 4 °C. All the tissues were cryoprotected by increasing the concentrations of sucrose to 30%. The samples were embedded in the OCT compound and frozen on dry ice. We collected all of the sections (20 μm) serially and stored them at −20 °C. Horizontal sections of the whole brain were cut for either p-S6 and NeuN staining or checking the existence of NS-PTEN. For assessing the regeneration of CST axons, serial sections of the spinal cord region, including the lesion site in the middle, 2.5 cm long, were cut in the sagittal plane.

### Immunofluorescence Staining of the Cortex

We followed the standard protocols for immunofluorescence staining. All antibodies were diluted in dilution buffer according to the manufacturer's recommendations. Rabbit antibodies to p-S6 (Ser235/236) (Cell Signaling Technology) were diluted 1:200 in a solution consisting of 10% normal goat serum (NGS) and 1% Triton X-100 in phosphate-buffered saline (PBS). Rabbit antibodies against NeuN (Millipore) and GFAP (Dako) were diluted 1:1,000 in a solution consisting of 5% NGS and 0.3% Triton X-100 in PBS. After being washed three times for 10 min with PBS and blocked for 30 min with a dilution solution, the sections were incubated with the primary antibodies overnight at 4 °C. The sections were then washed three times again for 10 min. Secondary antibodies (Alexa 488 or Alexa 647-conjugated goat antibodies to rabbits) were added to the sections and incubated for 1 h at room temperature. Hoechst (1:1000, Molecular Probes) was applied to the sections, and the samples were incubated for 10 min at room temperature. Sections were sealed with mounting media and a coverslip after being washed three times for 10 min each in PBS.

### Quantification of Neurons Containing NS-PTEN and p-S6 Intensity

For counting the sensorimotor neurons having NS-PTEN, digital images were taken around the brain injection site by using a confocal microscope (Zeiss LSM800) under a 25× objective to count the NeuN positive cells and the NeuN positive cells with NS-PTEN. The number of cells was counted in a square area of 0.36 mm^2^ with the brain injection site at the center of the view. At least 10 images from each group were quantified. The results were presented as the percentage of the number of neurons having NS-PTEN to the number of total neurons per time point.

To measure the intensity of p-S6, digital images were taken around the brain injection site with a confocal microscope (Zeiss LSM800) under a 25× objective. At least 10 tail images (area, 0.8 mm^2^) with the injection site at the center for 3 rats in each group were measured by using the Analyze Particles Tool in ImageJ (US National Institutes of Health).

### Measurement of NS-PTEN-Mediated CST Regeneration

For the analysis of axonal regeneration in rats with SCI receiving NS-PTEN treatment and AuNP treatment, we took mosaic images using a confocal microscope (Zeiss LSM800) under a 25× objective, which covered the lesion sites and the CST end in the spinal cord. After the rats experienced a contusion to their spinal cord, the lesion site appeared as a cavity with necrotic tissue. The rostral margin of the cavity was defined where healthy tissue was identified, and the end of the CST was labeled with BDA-Texas Red. We measured the length between the end of the CST and the rostral margin of the cavity.

### Fabrication of the Microfluidic Device

The microfluidic device was designed through AutoCAD software (Autodesk, CA, USA) to be fabricated with a two-step photolithography process.^75^ The 150 μm height microchannels to culture neural stem cells were made with SU-8 100 (Microchem Corp., MA, USA) photoresist, and a 20 μm height bridge microchannel for axonal outgrowth was fabricated with SU-8 2025 (Microchem Corp., MA, USA) photoresist. Polydimethylsiloxane (PDMS, Sylgard 184, Dow Corning Corp., MI, USA) was poured into the fabricated silicon wafers and was subsequently cured in an oven at 80 °C for 1 h. After curing PDMS, it was carefully peeled off the silicon wafer and bonded to a glass substrate using oxygen plasma (Femto Science, Korea).

### Axonal Injury and Regeneration in a Microfluidic Device

For neuronal induction, the neural stem cells were treated with NS-PTEN on day 1 in a differentiation medium without bFGF and EGF. The cells were subsequently cultured for 6 days. To mimic the environment of axonal injury in a microfluidic device, we treated the medium with NS-PTEN on day 1. On day 2, 1.5 μM thapsigargin (Sigma-Aldrich, MO, USA), a neurotoxin, was added to the medium and incubated in the microfluidic device for 1 h before being replaced with a differentiation medium. The cells were then cultured for 6 days. We treated the medium containing thapsigargin on day 1 to investigate the regeneration after axonal injury. On day 2, the medium containing NS-PTEN was treated, and neural differentiation was induced for 6 days.

### Analysis of Axonal Outgrowth in the Microfluidic Device

To confirm axonal guidance, the cells derived from neural stem cells were treated with 4% paraformaldehyde (YMS Korea, Korea) for 30 min and permeabilized by 1% Triton X-100 (Sigma-Aldrich, USA) in phosphate-buffered saline (PBS, Sigma-Aldrich, MO, USA) for 15 min. To reduce nonspecific protein binding, the cells were blocked with bovine serum albumin (BSA, Sigma-Aldrich, MO, USA) in PBS at room temperature for 2 h. The cells were incubated with Tuj1 (Stem Cell Technology, Canada) and nestin (Abcam, UK) primary antibodies at 4 °C overnight. The cells were subsequently rinsed with PBS and incubated overnight at 4 °C with Alexa Fluor 488-conjugated and Alexa Fluor 594-conjugated secondary antibodies. Finally, the cell nuclei were stained with 0.1 g mL^–1^ 4_,6-diamidino-2-phenylindole (DAPI, Thermo Fisher, MA, USA) in PBS for 1 h at room temperature. After washing with PBS in a microfluidic device, confocal laser-scanning microscope (LSM710, Carl Zeiss, Germany) images were obtained, and ImageJ (National Institute of Health, MD, USA) software was used to analyze the axonal outgrowth.

### Cell Type Delivery Immunostaining Quantification

The proportion of green-positive or red-positive cells was calculated using an automated method run with Python, slightly modified from the CellTagging Script developed by Guo et al.^76^ Briefly, as described in the developer’s paper, the background image was translated into binary images by thresholding the brightfield contrast. Specifically, the Otsu method was utilized to determine the threshold for detecting a contrast. The watershed method was used to segment the binary image into individual cells. The detected cells were then filtered to remove the cell aggregates and debris. The intensity of the fluorescent signal of interest (red/green and blue) was then quantified to determine the number of fluorescent positive cells and the ratio of fluorescent cells to nonfluorescent cells in the processed image. The processes were run with Python 3.11.1 and its libraries: scikit-image 0.19.2, numpy 1.24.0, matplotlib 3.6.2, seaborn 0.12.2, jupyter 5.2, and pandas 2.1.4.

### Neural Stem Cell Culture

Neural stem cells were extracted from the cortex tissues of E12 C57BL/6 mice (Daehan Biolink, Korea) and incubated with Accutase (Innovative Cell Technology, CA, USA) at 37 °C for 5 min. The neural stem cells were cultured on ultralow attachment surfaces to form neurospheres for 4 days in Dulbecco’s modified Eagle medium/nutrient mixture F-12 (DMEM/F-12) medium (Gibco, MA, USA) containing 20 ng mL^–1^ basic fibroblast growth factor (bFGF, R&D Systems, MN, USA), 20 ng mL^–1^ epidermal growth factor (EGF, Invitrogen, MA, USA), 1% N2 Supplement, 2% B27 supplements, and 1% penicillin-streptomycin (Gibco, MA, USA). Afterward, the neural stem cells were obtained from the culture plate and seeded into the inlet of the microfluidic device coated with poly-d-lysine and laminin. After incubation for 1 day, the neural stem cells were treated with NS-PTEN in a differentiation medium without bFGF and EGF.

### iPCS-NPC Cell Culture

hiPSC-NPCs were maintained using 0.5% B27, 0.5% N2, and 20 ng mL^–1^ bFGF in 1:1 Neurobasal: DMEM/F-12 proliferation media on Matrigel-coated tissue culture plastic in a humidified, 37 °C incubator with 5% carbon dioxide. Cell passaging was performed using Accutase. One day after iPSC-NPCs were seeded for experiments, bFGF was removed from the media for differentiation of the cells into neurons. The media was changed every 3 days for 7 days. For LPS-induced neurons, on day 7, cells were induced for 24 h at a concentration of 10 μg/mL to promote pro-inflammatory effects. On day 8, NS-PTEN or control conditions were delivered for 24 h prior to analysis of the cells. Cells were used from passage 10–18.

### Primary Human Astrocyte Cell Culture

Primary human astrocytes were obtained from Sciencell and cultured in astrocyte media with FBS, astrocyte growth supplement (AGS), and P/S. The media was changed every 3 days until the cells were at 60% confluency, and then the media was changed every 2 days until the cells reached 90% confluency. Cells were seeded in a dish, and TNF-α was delivered at 30 ng/mL with IFN-γ at 20 ng/mL to astrocytes for 24 h, as reported by other studies, to induce inflammatory effects in the cells. After inducing the cells, NS-PTEN was delivered at varying concentrations for 24 h prior to analysis. Cells were used from passages 2–5.

### Melting Temperature Shift Assay

Polyamides and dsDNA were analyzed in an aqueous buffer solution consisting of 10 mM sodium chloride and 10 mM sodium cacodylate at pH 7.0, containing 2.5% v/v DMF. Polyamides and dsDNA concentrations were 5 and 2.5 μM, respectively (2:1 stoichiometry). Absorbance at 260 nm was recorded from 95 to 10 °C at a rate of 1 °C min^–1^ using a spectrophotometer V-650 (JASCO) with a thermocontrolled PAC-743R cell changer (JASCO) and a thermal circulator F25-ED (Julabo).

### Synthesis of NS-PTEN

The NS-PTEN construct was synthesized as per our previous publication.^77^ In short, amine-terminated biomolecules were conjugated to a linker molecule, SH-PEG-COOH [thiol-PEG-carboxy2 kDaA (Creative PEGWorks, PBL-8073)]. The PEG linker was dissolved in dimethylformamide (DMF) to create a 50 mM solution. Similarly, 1 M *N*-hydroxysuccinimide (NHS) (Acros Organics) in DMF and 500 mM 1-ethyl-3-(3-(dimethylamino)­propyl) carbodiimide) (EDC) (Sigma) in DMF, and minimal amounts of water to promote solubility, were created. To activate the carboxyl group, EDC and NHS were added sequentially to the 50 mM PEG linker so that their final concentration was 50 mM, and the mixture was shaken at room temperature overnight. Next, 10 mM solutions of the various domains (i.e., the DBD, RD [WRPW–OH], and NLS [TAT]) were added to the activated PEG linker such that the final concentration of the PEGylated domains was 10 mM. Finally, a mixture containing 30% SH-PEG-RD, 30% SH-PEG-DBD, 30% SH-PEG-NLS, and 10% SH-PEG-COOH by volume was added to 1 mL of 10 nm AuNPs solution (TED-Pella: 15703–20) slowly over ice to create NS-PTEN. The construct was purified by washing with deionized water through a 10,000 MCWO filter (Millipore) to remove unreacted molecules and adjust the final volume. The dye-labeled NS-PTEN was designed by conjugating fluorescein to SH-PEH–COOH and replacing 2% of SH-PEG-COOH in the aforementioned solution with SH-PEG-Dye.

### In Vitro Western Blot Analysis

Cells were seeded in a 6 cm dish and treated with LPS or TNF-α and IFN-γ, followed by NS-PTEN for 24 h as described above for all conditions. Cells were then harvested, counted, and lysed with RIPA buffer before being put on ice for 30 min. The cells were centrifuged at 12000 g, and the supernatant was collected for testing protein levels. The supernatant was incubated at 95 °C. 10% running gels with 5% separating PAGE gels made in-house were run at 6 V/cm^2^ for 30 min and 10 V/cm^2^ for 1 h. The gels were transferred to 0.4 μM nitrocellulose membranes at 11 V/cm^2^ for 100 min and blocked for 1 h at room temperature with 5% BSA. The membranes were stained with primary antibodies overnight at 4 °C, and then stained with horseradish peroxidase (HRP)-linked secondary antibodies for 1 h at room temperature. Antibody dilutions were based on manufacturer's recommendations. The membranes were activated with SuperSignal West Femto Maximum Sensitivity Substrate, analyzed with a Thermo Fischer iBright UV illuminator, and quantified with Thermo Fischer iBright analysis software [version 5.2.2].

### RT-q-PCR

Total RNA was extracted from cells using trizol reagent; 1 μg of RNA was reverse transcribed into cDNA using the Superscript III kit. qPCR was performed using KAPA SYBR FAST qPCR (Roche, Cat. No. KK4601) with a CFX96TM real-time PCR detector (Bio-Rad). Relative mRNA levels were normalized to β-actin or Gapdh mRNA levels for each target gene.

### MAP2/Tuj1/Hoechst Staining

The iPSC-NSCs were seeded on 12-well cell culture plate at a density level of 150,000 cells, differentiated for 10 days, cultured with media for the negative control and media + LPS for 24 h for the rest of the conditions. Finally, 5 μg/mL NS-PTEN, 10 μg/mL NS-PTEN, 5 μg/mL NS-PTEN + rapamycin, 10 μg/mL NS-PTEN + rapamycin, 5 μg/mL AuNP, 10 μg/mL AuNP, 5 μg/mL AuNP + rapamycin, or 10 μg/mL AuNP + rapamycin for 24 h. The cells were fixed with 4% paraformaldehyde for 20 min. They were then washed with DPBS three times, and the cells were blocked with a 0.5% BSA and 0.01% Triton-X-100 blocking solution in dPBS (Invitrogen, Waltham, MA, USA). The cells were stained with anti-pTau (neurodegeneration marker, 1:400, 488 nm green) and anti-Tuj1 (neuron marker, 1:400, 594 nm red) solution. The cells were incubated overnight at 4 °C and then incubated in Alexa 488 and 594 secondary antibodies (both 1:200, Invitrogen) for 2h at RT. Then, the cells were stained with Hoechst (1:10,000, 15 min) for the nuclei staining. The ROI area was normalized to 100%. The cells were detected by using a confocal laser-scanning microscope (LSM 780, Carl Zeiss, Jena, Germany).

### Cell Proliferation Assay

The Cell Counting Kit 8 (Abcam, Waltham, MA, USA) was utilized to assess the cell viability test.

### In Vivo Western Blot Analysis

Spinal cord tissue samples were pulverized into a fine powder in liquid nitrogen and homogenized in RIPA lysis buffer (Sigma-Aldrich, St. Louis, MO, USA) supplemented with a Halt Protease and Phosphatase Inhibitor Cocktail (Thermo Fisher Scientific). The homogenates were agitated for 2 h at 4 °C and subsequently centrifuged at 13,000 rpm for 20 min at 4 °C to remove debris. The protein concentration of the supernatant was determined using a Bradford protein assay (Bio-Rad Laboratories, Hercules, CA, USA). Protein samples (20 μg) were mixed with 5× sample loading buffer (Cat. No. PB700; RAON, Yongin, Korea) and denatured at 95 °C for 10 min. The samples were separated on 10% SDS-polyacrylamide gels prepared using acrylamide/bis solution and buffers from Bio-Rad Laboratories. Following electrophoresis, proteins were transferred onto polyvinylidene difluoride (PVDF) membranes at 100 V for 2 h. The membranes were blocked with 5% BSA in TBS-T for 1 h at room temperature and then incubated with specific primary antibodies overnight at 4 °C. After washing with TBS-T, the membranes were incubated with HRP-conjugated Goat anti-Rabbit (Cat. No. 31460) or Goat anti-Mouse (Cat. No. 31430) secondary antibodies (Invitrogen, Carlsbad, CA, USA) for 1 h at room temperature. Protein bands were visualized using an enhanced chemiluminescence (ECL) substrate (Thermo Fisher Scientific) and imaged using a chemiluminescence imaging system. Band intensities were quantified by using ImageJ software. The list of antibodies used is provided in Table S1.

### In Vivo Gene Expression Analysis

The expression levels of target genes were quantified by the quantitative reverse transcription polymerase chain reaction (RT-qPCR) of mRNA extracted from cell culture. The total RNA was extracted using TRIzol Reagent (Life Technologies), and 1 μg of the total RNA was used as a template for a reverse transcription reaction to generate the first-strand complementary cDNA using the Superscript III First-strand Synthesis System (Life Technologies) according to the manufacturer’s protocol. The first-strand cDNA was then used for a qPCR reaction with gene-specific primers in the presence of a Power SYBR Green PCR Master Mix (Applied Biosystems) on a StepOnePlus Real-Time PCR System (Applied Biosystems). The *C*_t_ values were normalized to GAPDH, which was selected as the endogenous control. The standard cycling conditions were applied to all of the reaction conditions with a melting temperature of 60 °C. All gene-specific primers were obtained from the PrimerBank database and are listed in Table S3.

### Tissue Processing and Preparation for Staining

Spinal cord samples were collected following euthanasia, which was performed using perfusion. The perfusion process involved the use of 50 mL of 0.9% saline and 4% (w/v) paraformaldehyde (PFA). The tissues were then fixed in 4% PFA for 24 h. Subsequent dehydration was carried out using a series of ethanol solutions of increasing concentrations (70, 80, 90, 95, and 99.9%), followed by immersion in 100% xylene and embedding in paraffin. For histological examination, longitudinal sections of the spinal cord were prepared. Paraffin blocks were created and sectioned at a thickness of 5 μm using a Leica microtome. Prior to H&E, LFB staining, and immunofluorescence analyses, the slide samples underwent a deparaffinization process in 100% xylene. This was followed by rehydration through a descending ethanol series (99.9, 95, 90, 80, and 70%).

### Ex Vivo Fluorescence Imaging of NS Distribution

To circumvent signal attenuation caused by dorsal hair scattering, ex vivo imaging was performed to monitor the biodistribution of NS-PTEN. Animals were sacrificed at designated time points via inhalation of CO_2_ in strict accordance with IACUC protocols. Spinal cord tissues were immediately harvested and positioned on the imaging stage of a Davinch-Invivo Imaging System (INV-16M, Davinch, Korea). This system is equipped with a high-sensitivity cooled CCD camera (−50 °C) and a motorized 12-position filter wheel. Fluorescence images were acquired under standardized exposure conditions, and signals were quantified as total radiant efficiency (p/s/cm^2^/sr)/(μW/cm^2^) using the manufacturer’s analysis software, with all values normalized to background tissue fluorescence.

### Hematoxylin and Eosin (H&E) Staining for Histological Examination

H&E staining was performed at DPI-1 and 28 to conduct histological examination of spinal cord samples. After the preparatory steps, all slide samples were stained with Harris hematoxylin solution. The staining process was followed by brief bleaching in 1% alcoholic hydrochloric acid. The slides were then neutralized using a 1% ammonia solution (Junsei) and counterstained with an eosin solution. Poststaining, the slide samples underwent dehydration through a graded series of ethanol solutions (70, 80, 90, 95, and 99.9%) and were subsequently cleared in 100% xylene. For the final mounting step, a liposolubility-based mounting medium, Canada Balsam (Junsei), was applied before placing the cover glass. Digital images of the H&E-stained samples were captured using a Zeiss Axio Scan.Z1 digital slide scanner (Carl Zeiss) and analyzed with Zen 3.1 Blue edition software (Carl Zeiss).

### Luxol Fast Blue (LFB) Staining for Myelinated Axons

To visualize, myelinated axons within the spinal cord were obtained at DPI-1 and 28. Following preparation, all slide samples were processed using an LFB staining kit (Abcam) according to the manufacturer’s instructions. The samples were immersed in LFB solution at 60 °C for 2 h, followed by brief treatment with 0.05% lithium carbonate solution and 70% alcohol for 20 s. Cresyl echt violet was utilized to counterstain the cytoplasm. After the staining process, the slides underwent dehydration through a series of ethanol solutions (70, 80, 90, 95, and 99.9%) and were then cleared with 100% xylene. The slides were mounted by using Canada Balsam (Junsei) on cover glasses. Digital images of the LFB-stained samples were captured with a Zeiss Axio Scan.Z1 digital slide scanner (Carl Zeiss) and analyzed using Zen 3.1 Blue edition software (Carl Zeiss).

### Immunofluorescence for Detecting Spinal Cord Regeneration

Immunofluorescence was employed to identify specific antigens indicative of spinal cord regeneration at DPI-1 and 28. Following initial sample preparation, antigen retrievals were conducted using either pepsin solution (GBI Laboratories) or tris-ethylenediamine tetraacetic acid (EDTA) buffer solution (Biosesang) for 10 min. The samples were then blocked with 1% (w/v) bovine serum albumin (BSA) solution for 90 min. Subsequently, primary antibodies were applied to the spinal cord tissues and incubated at 4 °C for 24 h. The following day, secondary antibodies were incubated on spinal cord tissue at room temperature (24 °C) for 1 h. Counterstaining was performed with DAPI (1:3000; Thermo Fisher) for 10 min at room temperature. Dako Fluorescence Mounting Medium (Agilent, Santa Clara) was used for mounting. Detailed antibody information is provided in Table S2. Pearson’s colocalization coefficient for the immunofluorescence data was calculated using AutoQuant X2 software (64). Quantitative immunofluorescence results were graphed as the percentage of the positive area in the designated region of interest at the lesion site and analyzed using the Zen Zeiss software.

### Behavioral Test

Motor function in the hind limbs of all experimental animals was assessed using the Basso-Beattie-Bresnahan (BBB) locomotor rating scale. Evaluations were conducted at various DPIs. The BBB scale, which ranges from 0 to 21, was used for this assessment (65). Animals were observed in an open field, and two investigators, blinded to the experimental groups, analyzed the differences between the groups. To quantitatively assess gait dynamics and motor recovery following SCI, footprint analysis was performed. Rats were allowed to walk across a straight, enclosed runway (110 cm length × 10 cm width) lined with white absorbent paper leading to a darkened goal box. To distinguish between the left and right sides, the animals’ hind paws were dipped in nontoxic blue and black ink, respectively. For analysis, tracks containing at least three consecutive uninterrupted gait cycles were selected. Based on the digitized footprints, three key gait parameters were measured: stride length (the distance between consecutive steps of the same paw) and stride width (the perpendicular distance between the left and right paws). These values were used to quantify the functional recovery. Assessments were conducted on the final day of the experiment.

To evaluate sensory dysfunction and mechanical allodynia following injury, nociceptive sensitivity was assessed using an electronic Von Frey system (Ugo Basile, Model 37000–007). The device applied a gradually increasing force at a constant rate of 10 g/s through a calibrated pressure probe. Rats were individually placed in transparent acrylic observation chambers positioned over a metal mesh platform to allow free access to the plantar surface of the hind paw. Following a 10 min acclimatization period, the probe was applied perpendicularly to the midplantar area until a withdrawal reflex (paw lifting, brisk retraction, or flinching) was detected. The mechanical withdrawal threshold (in grams) was automatically recorded, corresponding to the force required to elicit a response. Measurements were averaged over three consecutive trials with an intertrial interval of at least 5 min. Assessments were conducted 1 day prior to injury (baseline) and at DPI 0, 7, 14, and 28. To evaluate locomotor coordination and neuromuscular endurance following SCI, a Rotarod performance test was conducted using a motorized rotating rod system. Animals were subjected to a linear acceleration protocol ranging from 5 to 15 rpm over a 300 s period. The latency to fall (in seconds) was recorded as the primary outcome measure, with a predefined cutoff time of 300 s. To minimize variability associated with motor learning, rats underwent three training trials per day for three consecutive days prior to surgery. Testing was performed at baseline and on DPI values of 0, 7, 14, and 28. For each session, the mean value of three trials was used for statistical analysis. All behavioral assessments were performed in a controlled, quiet environment by an investigator blind to the experimental groups.

### Kinematic Gait Analysis Using DeepLabCut

To evaluate hindlimb locomotor recovery, we employed markerless pose estimation using DeepLabCut (DLC) software (version 2.7). Videos of overground locomotion were recorded from a lateral perspective. A subset of frames was extracted and manually labeled to train the ResNet-50 neural network. The skeletal model included four key anatomical landmarks on the hindlimb: the Tail base, Heel (calcaneus/tarsal region), Metatarsus (metatarsal region), and Digits (phalanges). The network was trained for 3000 iterations until the loss plateaued. Postprocessing and kinematic calculations were performed using custom Python scripts. We analyzed the vertical displacement of the Heel marker to determine the hindlimb swing amplitude during the swing phase of the gait cycle. To quantify the hindlimb swing amplitude, the vertical excursion (*y*-axis displacement) was calculated as the difference between the peak height during the swing phase and the baseline height during the stance phase. Pixel coordinates were converted to physical units (cm) by using a calibration factor derived from the standard kinematic profile of the Sham group (mean swing amplitude normalized to ∼2.0 cm).

### Motor-Evoked Potential (MEP) Recording

Motor-evoked potentials (MEPs) were recorded to assess corticospinal functional recovery following SCI. Rats were anesthetized throughout the procedure, and the body temperature was maintained at 33 °C using a feedback-controlled heating pad. Animals were secured in a stereotaxic frame to ensure a stable electrode placement. A concentric bipolar stimulation electrode (CBAPB75, FHC Inc.) was stereotaxically inserted into the primary motor cortex using the following coordinates relative to bregma: anterior–posterior (AP) 2.0–2.5 mm, medial–lateral (ML) ± 1.5–2.2 mm, and dorsal–ventral (DV) – 2 mm. Electrical stimulation was delivered using a DS3 isolated current stimulator (Digitimer) controlled by SynapseLite software (Build 90–39174P, Tucker-Davis Technologies). Monophasic square-wave pulses (100 ms duration; 0.5–30 mA intensity; delivered every 500 ms) were applied to evoke muscle responses. Muscle MEPs were recorded from the tibialis anterior using two custom unipolar stainless-steel needle electrodes inserted intramuscularly. Two additional needle electrodes were placed subcutaneously along the upper back to serve as the reference and ground electrodes, with the ground electrode in contact with the surgical table. Signals were routed through the AC16LR analogue headstage (Tucker-Davis Technologies). Electrophysiological signals were amplified, digitized, and recorded using SynapseLite software. Raw traces were inspected offline, and response peaks were identified based on waveform deflections exceeding background noise levels. At the end of the recording session, the rats were euthanized.

### Blood Test Analysis

At the predetermined end points, blood samples were collected for serum biochemistry and hematological analysis. For biochemical analysis, blood was collected into 0.5 mL serum separator tubes containing a clot activator and gel (Microcollect, ref. 111205; GSMEDITECH, Wonju, Korea). The samples were allowed to clot at room temperature for 30 min to ensure complete coagulation. Subsequently, serum was separated by centrifugation at 5500 rpm for 10 min at 4 °C. For hematological assessment (Complete Blood Count, CBC), whole blood was collected into BD Microtainer tubes containing BD Microtainer Tubes with K2E (K2EDTA) (ref. 365974; Becton, Dickinson and Company, Franklin Lakes, NJ, USA). These samples were immediately inverted gently to mix with the anticoagulant and stored on ice to preserve the cell morphology. All samples were transported to the Laboratory Animal Center at CHA University for immediate analysis using an automated hematology and biochemistry analyzer.

### Enzyme-Linked Immunosorbent Assay

The concentrations of inflammatory cytokines, including Interleukin-1 beta (IL-1β) and Interleukin-10 (IL-10), were quantified by using commercial sandwich ELISA kits. The Rat IL-1β (Cat. No. RK00009) kit was purchased from ABclonal, and the Rat IL-10 kit (Cat. No. E0108Ra) was obtained from BT LAB. Sample preparation was performed using an Eppendorf 5430 R centrifuge (Eppendorf, Hamburg, Germany). Cerebrospinal fluid (CSF) samples were centrifuged at 3000 rpm for 20 min at 4 °C. Supernatants were collected and stored at −80 °C until analysis. Samples and standards were added to antibody-coated wells and incubated at 37 °C. Following the addition of biotin-conjugated antibodies and Streptavidin-HRP, the reaction was visualized using a TMB substrate. The reaction was terminated with an acidic stop solution, and the optical density (OD) was measured at 450 nm by using a microplate reader. Concentrations were calculated based on standard curves established by plotting the OD values against the known concentrations. A second-order polynomial regression model was employed to fit the data, ensuring a coefficient of determination (*R* ^2^) > 0.99 for all assays.

### TUNEL Staining

Apoptotic cells in the spinal cord tissue were detected using the DeadEnd Fluorometric TUNEL System (Cat. No. G3250; Promega, Madison, WI, USA) following the manufacturer’s protocol. Paraffin-embedded sections were deparaffinized in xylene and rehydrated through a graded ethanol series. Permeabilization was performed by incubating sections with Proteinase K (20 μg/mL) for 8–10 min at room temperature. After washing with PBS, the sections were equilibrated and incubated with the TdT reaction mix, containing recombinant Terminal Deoxynucleotidyl Transferase (rTdT) and fluorescein-12-dUTP, at 37 °C for 60 min in a humidified, dark chamber. The reaction was terminated by immersing the slides in 2× SSC solution for 15 min. Nuclei were counterstained with DAPI (4’,6-diamidino-2-phenylindole) to visualize cell positioning. Images were captured using a fluorescence microscope.

### Statistical Analysis

All statistical analyses were performed using GraphPad Prism 10 (GraphPad Software). For comparisons involving four independent groups, an ordinary one-way ANOVA was conducted under the assumption of Gaussian-distributed residuals and equal standard deviations, as specified in Prism’s analysis parameters. Tukey’s post hoc multiple-comparison test with a single pooled variance was applied to evaluate pairwise differences among all groups, and multiplicity-adjusted *P*-values were reported. The family-wise significance level was set at α = 0.05 with a 95% confidence interval. All statistical tests were two-tailed. Data are presented as mean ± SD unless otherwise stated. All analyses were performed by investigators blinded to the experimental groups. For behavioral assessments (BBB, Von Frey, and Rotarod tests), differences between groups across multiple time points were analyzed using a two-way repeated measures analysis of variance (ANOVA) with the Geisser-Greenhouse correction. Tukey’s multiple comparisons test was utilized for post hoc analysis to determine significant differences between groups at specific time points. A *P*-value of <0.05 was considered statistically significant.

## Supplementary Material













## Data Availability

The data that support the findings of the study are available from the corresponding authors upon reasonable request.

## Notes

Precise Impactor (MP4)
Locomotion abilities of Sham rats DPI-28 (MP4)
Locomotion abilities of SCI rats DPI-28 (MP4)
Locomotion abilities of PBS rats DPI-28 (MP4)
Locomotion abilities of NS-PTEN rats DPI-28 (MP4)
Melt temperature shift assay, further in vitro experimentation in neurons and astrocytes, cell uptake and nuclear colocalization in vivo, axon regeneration in a microfluidic model, and further in vivo results, including the model for inducing SCI, spatiotemporal biodistribution and clearance kinetics of NS-PTEN, NS-PTEN colocalization with neurons in vivo, time-dependent Western blot, attenuation of inflammation in vivo, and recovery of blood-spinal cord barrier, H&E staining, open-field test, as well as recovery of motor-evoked potential (PDF)
