# Supplementary material for: NanoScript-Enabled Nonviral Transient Repression of Phosphatase and Tensin Homolog for Axonal Regeneration and Central Nervous System Injury Repair
Source: ACS Nano. 2026 Feb 19;20(8):6582–608. doi: 10.1021/acsnano.5c13020 (PMC12961949; doi:10.1021/acsnano.5c13020)
Supplement: Supplementary file 6 [file nn5c13020_si_006.pdf]

**SUPPLEMENTARY MATERIALS FOR**

# NanoScript-Enabled Non-Viral Transient Repression of PTEN for Axonal Regeneration and Central Nervous System Injury Repair

Brandon Conklin[1⊥], Yanting Liu[2⊥], Sarah Nevins[1⊥], Byeong-Gwan Song[2,3], Sy-Tsong Dean Chueng[1], Qiu Xiaowen[4,5], Sungyun Kim[1], Heyin Cheung[1], Seong Bae An[2], JongMin Lee[6], Bong Geun Chung[6], Wise Young[4], Dongming Sun[4], Hiroshi Sugiyama[7], Inbo Han[2,3*], and Ki-Bum Lee[1*]

[1] Department of Chemistry and Chemical Biology, Rutgers University, 123 Bevier Road, Piscataway, NJ 08854, U.S.A.

[2] Department of Neurosurgery, Cha University School of Medicine, Seongnam-Si, Gyeonggi-do, 13496, South Korea

[3] Department of Life Science, CHA University School of Medicine, 335 Pangyo-ro, Bundang-gu, Seongnam-si, Gyeonggi-do, 13488, Republic of Korea.

[4] W.M. Keck Center for Collaborative Neuroscience and the Department of Cell Biology and Neuroscience, Rutgers University, Piscataway, NJ, 08854, USA

[5] Department of Orthopedics, The Fourth Affiliated Hospital of School of Medicine, and International School of Medicine, International Institutes of Medicine, 866 Yuhangtang Road, Hangzhou, Zhejiang University, 310058, China.

[6] Department of Mechanical Engineering, Sogang University, 35 Baekbeom-ro, Mapo-gu, Seoul, 04107, Republic of Korea

[7] Institute for Integrated Cell-Material Sciences (WPI-iCeMS), Kyoto University, Yoshida-Ushinomiyacho, Sakyo-Ku, Kyoto, 606-8501, Japan

**⊥: Equal first author**

Brandon Conklin, Yanting Liu, Sarah Nevins contributed equally to this work and share co–first authorship

CORRESPONDING AUTHORS:

Prof. Ki-Bum Lee

Department of Chemistry and Chemical Biology, Rutgers, The State University of New Jersey

Tel. +1-732-445-2081; Fax: +1-732-445-5312

Email: kblee@rutgers.edu

Website: https://kblee.rutgers.edu/

Prof. Inbo Han

Department of Neurosurgery, Cha University School of Medicine, Seongnam-Si, Gyeonggi-do, South Korea

Email: haninbo@gmail.com

**KEYWORDS:** Axon Regeneration, Spinal Cord Injury, Non-viral Gene Therapy, Artificial Transcription Factor, Transient Gene Silencing, Nanoparticle therapeutics, Nanoparticle-based Gene Delivery, Regenerative Medicine

# TABLE OF CONTENTS


<u>SUPPORTING INFORMATION</u>

**Figure S1: Target DNA and hairpin polyamide melting temperature shift assay**

**Figure S2: Cell Tracker quantification demonstrating preferential neuronal internalization**

**Figure S3: Effects of NS-PTEN in mature neurons**

**Figure S4: Effects of NS-PTEN in LPS induced neurons**

**Figure S5: Effects of NS-PTEN in TNF$\alpha$ and IFN$\gamma$-induced astrocytes**

**Figure S6: NanoScript-PTEN fate and mTOR activation**

**Figure S7: *In vivo* delivery of NanoScripts into the targeted neural cells and their nucleus localization**

**Figure S8: NanoScript-PTEN localized in the nucleus of cells expressing p-S6 *in* vivo**

**Figure S9: Sensorimotor delivery of NanoScript-PTEN promotes *Pten* repression and leads to axonal regeneration into the injury site**

**Figure S10: NanoScript-PTEN promotes axonal regeneration following emulated spinal cord injury in a microfluidic model**

**Figure S11: Design of microfluidic device**

**Figure S12: Temporal growth of axons following NS-PTEN treatment**

**Figure S13: Axonal injury and regeneration in the microfluidic device**

**Figure S14: Induction of contusive spinal cord injury to SD rat with a precise impactor**

**Figure S15: Spatiotemporal biodistribution and clearance kinetics of NS-PTEN.**

**Figure S16: Colocalization of NS-PTEN with the nucleus at DPI-1**

**Figure S17: Change of PTEN expression with AuNP and NS-PTEN overtime**

**Figure S18: NS-PTEN attenuates inflammation and resulted in BSCB recovery in the chronic phase.**

**Figure S19: Systemic biocompatibility evaluation of NS-PTEN.**

**Figure S20. Open Field Test (OFT) performance at DPI-28.**

**Figure S21. NS-PTEN promotes recovery of motor-evoked potential after spinal cord injury.**

**Table S1: Western blot antibody information**

**Table S2: Immunofluorescence antibody information**

**Table S3: Primers for experiments**

**Supplemental Video Legend 1: Precise Impactor**

**Supplemental Video Legend 2: Locomotion abilities of Sham rats DPI 28**

**Supplemental Video Legend 3: Locomotion abilities of SCI rats DPI 28**

**Supplemental Video Legend 4: Locomotion abilities of PBS rats DPI 28**

**Supplemental Video Legend 5: Locomotion abilities of NS-PTEN rats DPI 28**

**References**

**OTHER SUPPLEMENTARY MATERIALS FOR THIS MANUSCRIPT INCLUDE THE FOLLOWING:**

**Supplemental Videos 1-5**

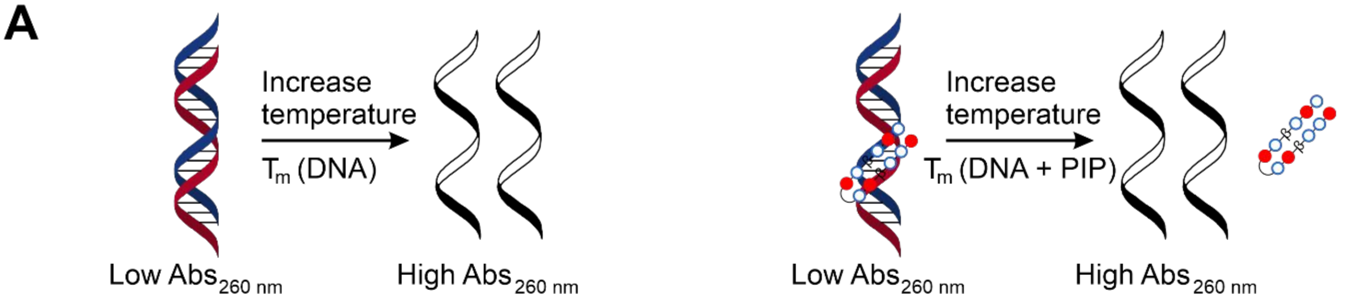


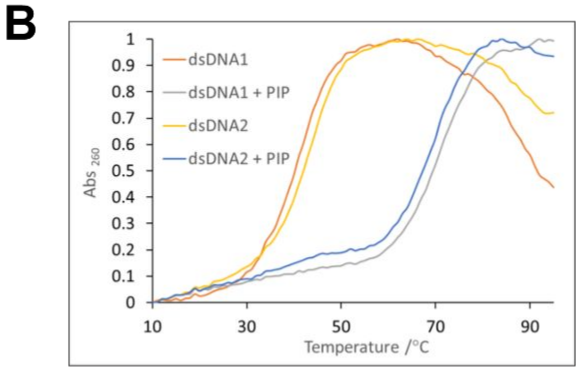


C

| | 1st | 2nd | 3rd | Tm/ºC | ΔTm/ºC |
|---|---|---|---|---|---|
| dsDNA1 | 40.89 | 41.26 | 41.86 | 41.34 (0.40) | - |
| dsDNA1 + PIP | 70.59 | 73.10 | 72.92 | 72.20 (1.14) | 30.87 |
| dsDNA2 | 44.16 | 44.15 | 44.37 | 44.23 (0.10) | - |
| dsDNA2 + PIP | 69.67 | 72.31 | 72.12 | 71.37 (1.20) | 27.14 |

**Fig. S1: Target DNA and hairpin polyamide melting temperature shift assay**. (**A**) Schematic illustration demonstrating experimental conditions. (**B**) Representative melting temperature shift curves of both target DNA sequences in the presence and absence of hairpin polyamide. (**C**) Melting temperature values obtained.

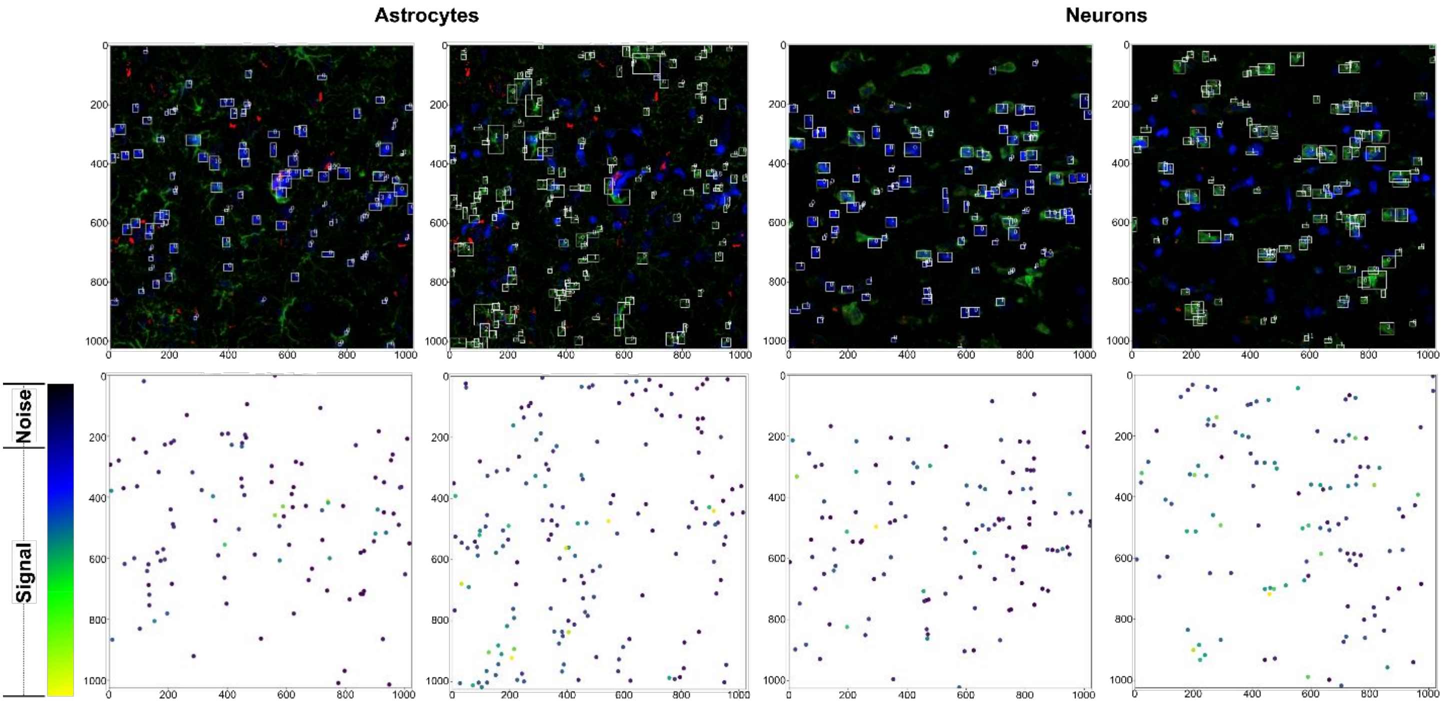


**Fig. S2: Cell Tracker quantification demonstrating preferential neuronal internalization**. Cell Tracker analysis highlighting the preferential internalization of NanoScript-PTEN into neurons (26.9%) over astrocytes (19.6%).

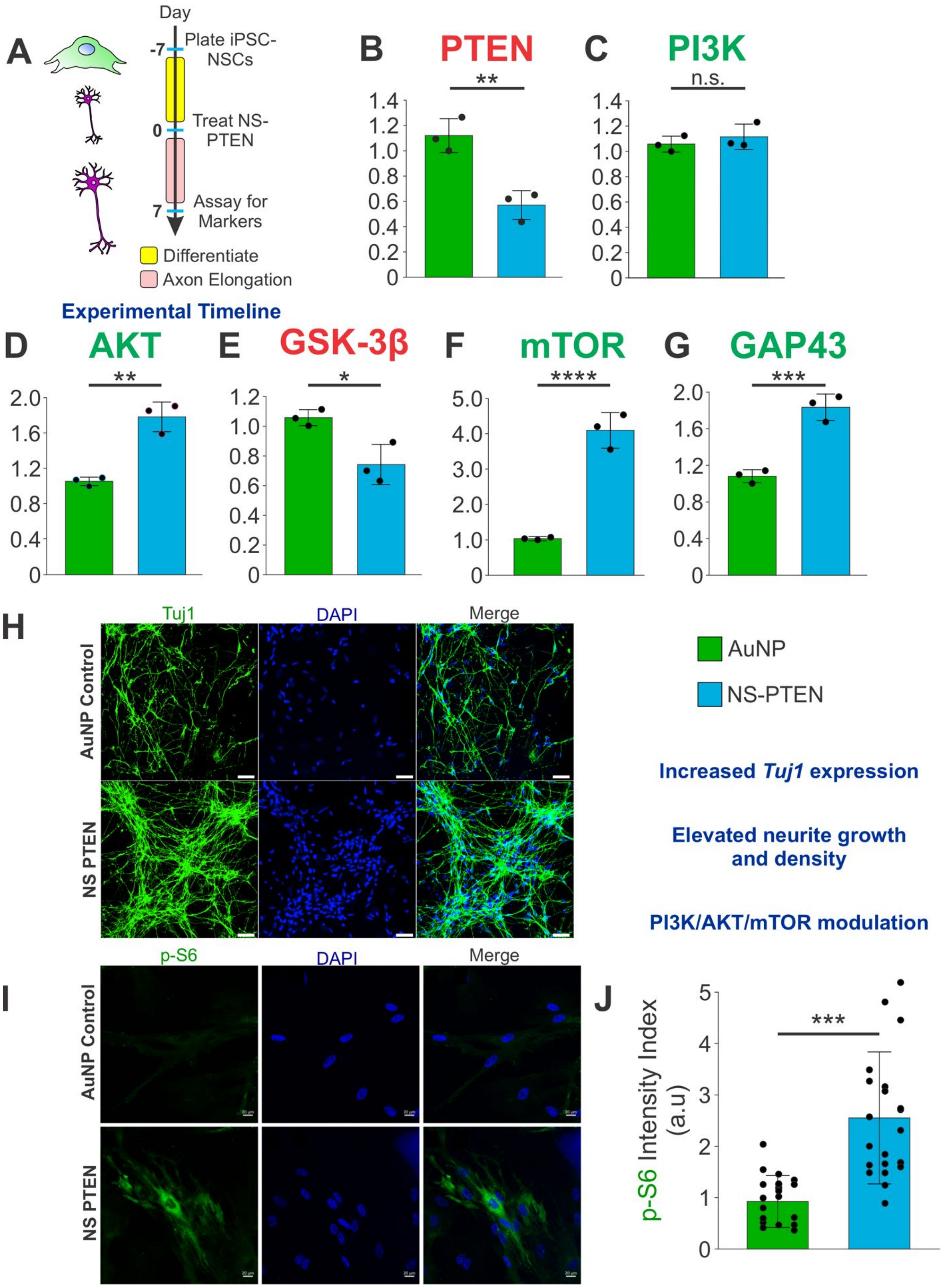


**Figure S3: Preliminary testing of NS-PTEN promotion of axonal regeneration and activation of the mTOR pathway.** (**A-F**) qRT-PCR (*n* = 3) results demonstrating NS-PTEN modulates various genes in the anticipated manner that serve as genetic barriers to axonal regeneration in iPSC-NPC-derived neurons. (**G**) Representative immunofluorescence image of iPSC-NPC-derived neurons following AuNP and NS-PTEN treatment. Scale bar = 10 µm. (**H**) Average neurite length following treatment measures in 13 images. (**I**) Average Tuj1 intensity (n = 13). (**J**) Immunofluorescence (*n* = 22) and quantification of p-S6 in iPSC-NPC-derived neurons following treatment. Scale bar 20 µm**.** Mean ± SEM, t-test. *$P < 0.05$; **$P < 0.01$; ***$P < 0.001$; **** $P < 0.0001$; n.s. not significant.

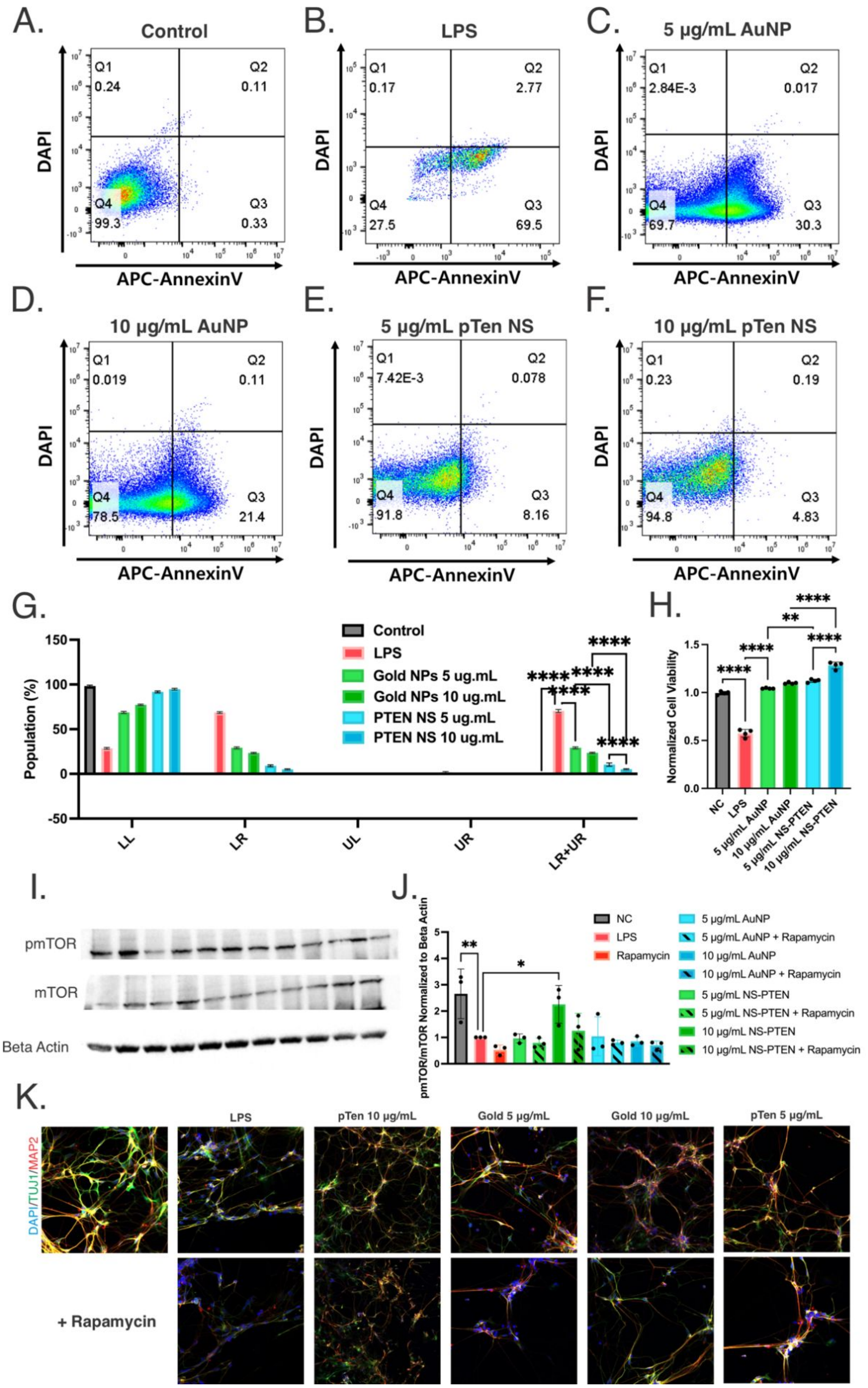


**Fig. S4: Further data showing effects of NS-PTEN to LPS-induced neurons differentiated from iPSC-NPCs. (A-F)** Flow cytometry graphs of LPS-induced neurons after APC Annexin V staining. (**G**) Comparison of flow cytometry apoptosis results across conditions. (**H**) Cell viability in LPS-induced neurons upon AuNP and NS-PTEN treatment. (**I**) Western blot of pmTOR, mTOR, and beta actin in LPS-induced cells upon AuNP and NS-PTEN treatment alone or co-delivered with rapamycin for 24 hours. (**J**) Quantification of pmTOR/mTOR ratio in neurons induced with LPS. (**K**) Immunostaining of MAP2 and TUJ1 in LPS-induced cells upon AuNP and NS-PTEN treatment alone or co-delivered with rapamycin for 24 hours.

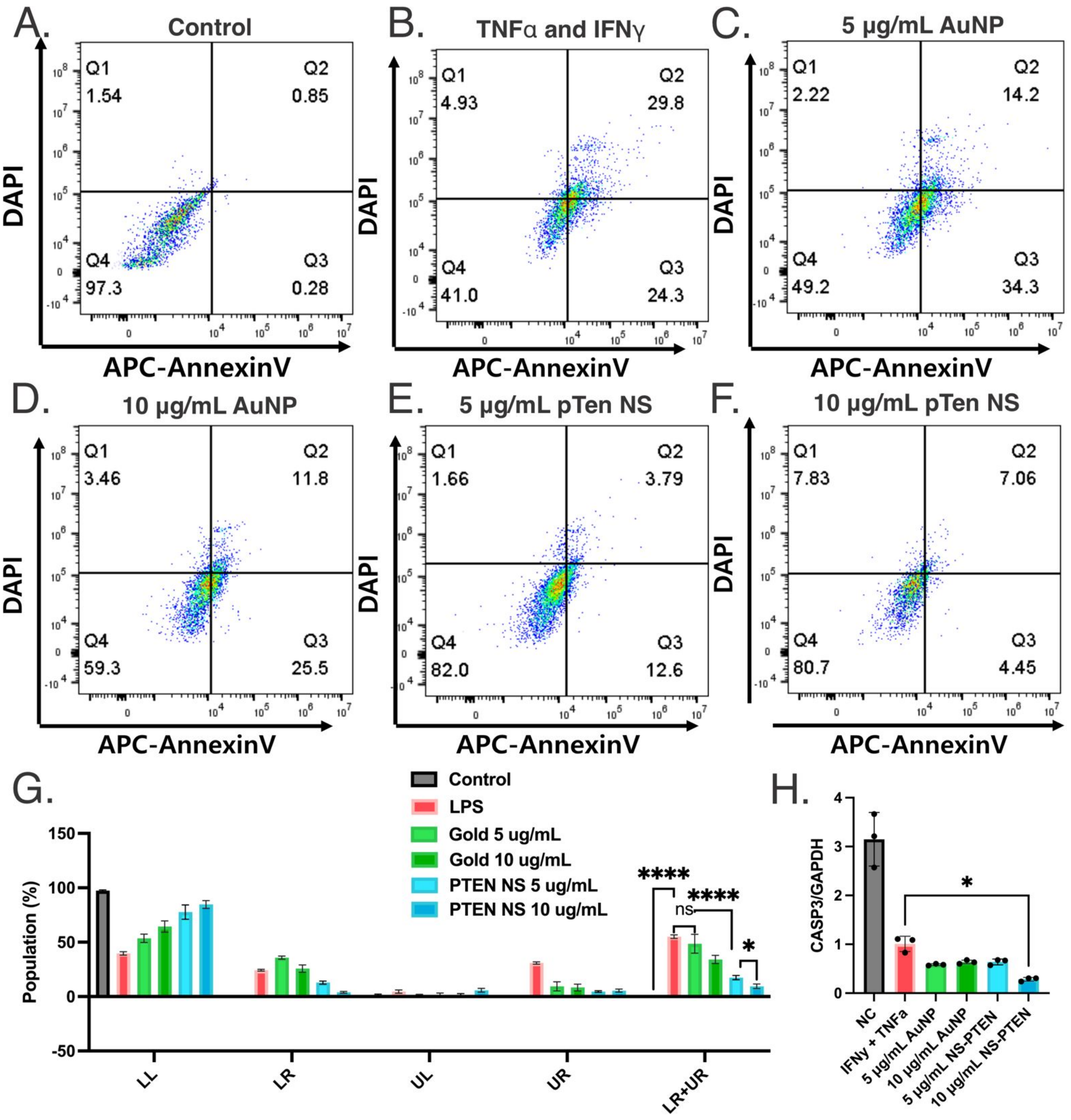


**Fig. S5: Further data showing effects of NS-PTEN to TNFα and IFNγ-induced astrocytes. (A-F)** Flow cytometry graphs of TNFα and IFNγ-induced astrocytes after APC Annexin V staining. (**G**) Comparison of flow cytometry apoptosis results across conditions. (**H**) RT-q-PCR of relative CASP3 expression normalized to GAPDH.

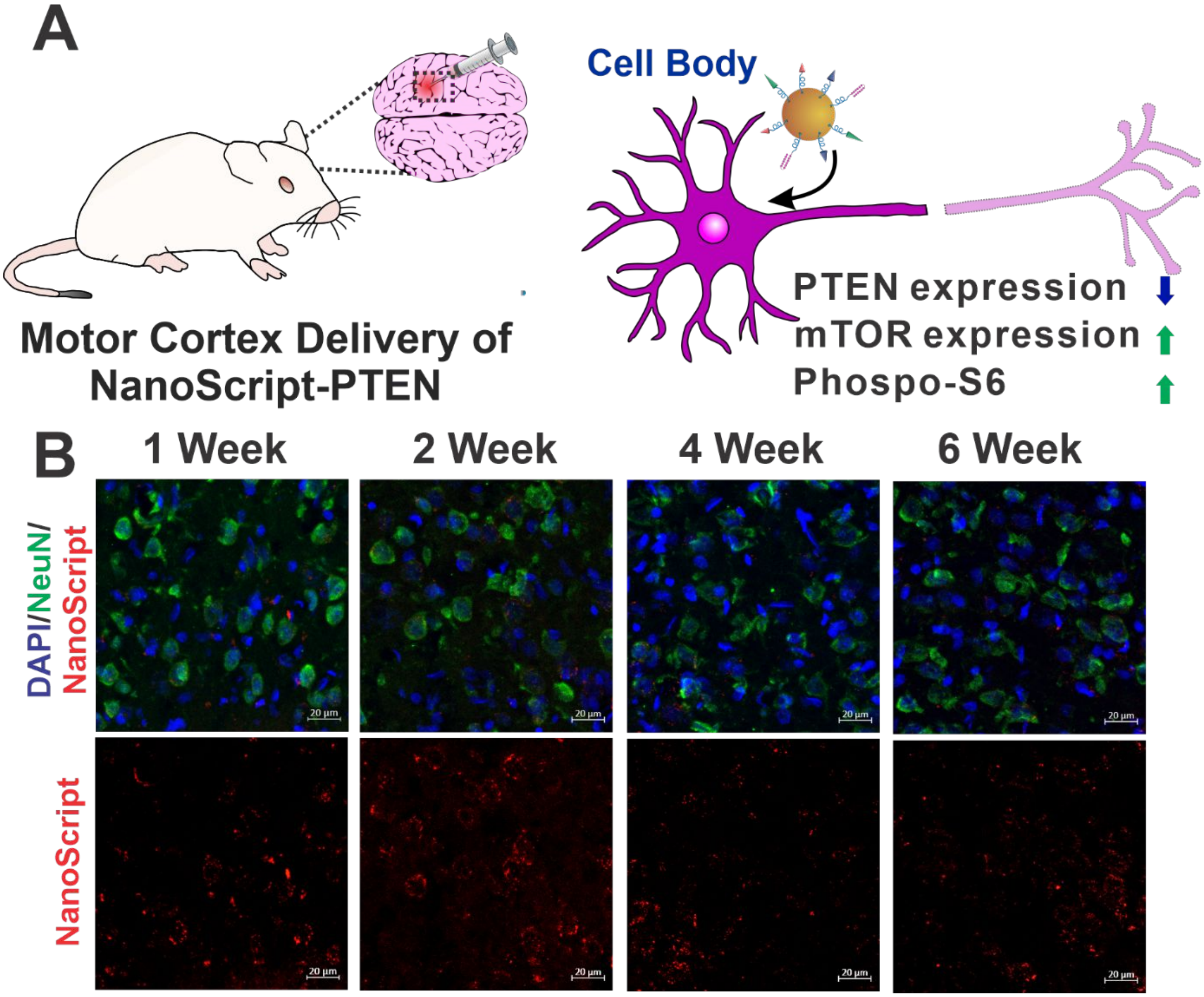


**Fig. S6: NanoScript-PTEN fate and mTOR activation**. (**A**) Schematic illustration of NanoScript-PTEN being delivered to the soma of neurons in the sensorimotor cortex following craniotomy. (B) Immunofluorescent images of neurons containing NanoScript-PTEN over 6 weeks. Scale bar: 20 µm.

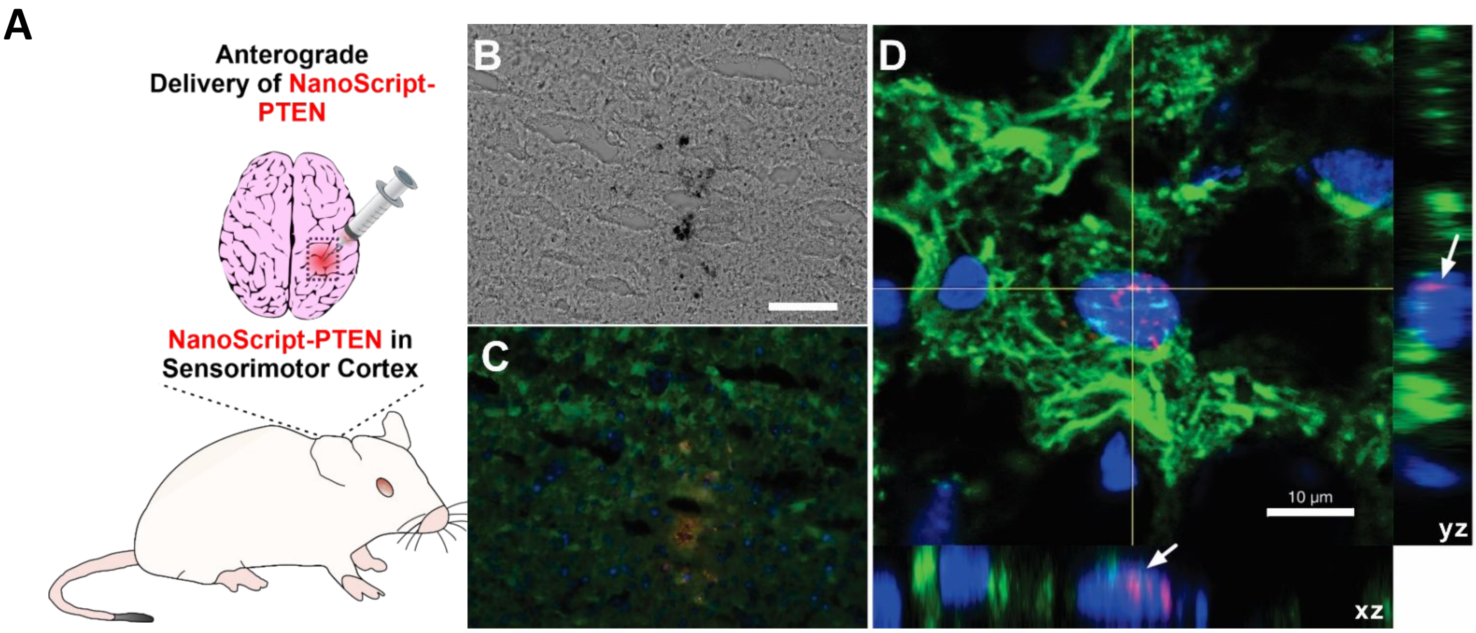


**Fig. S7:** ***In vivo*** **delivery of NanoScripts into the targeted neural cells and their nucleus localization. (A)** Schematic diagram of cranial injection of NanoScript-PTEN into rats. **(B-D)** Cross-sectional brain slice 3 days after a rat received *in vivo* intracranial NanoScript-PTEN delivery. The tissue was immunostained with Hoechst (blue), neurofilament antibody (green), and Alexa-dye labeled NanoScript (red), and imaged with a confocal microscope showing the nuclear localization of NanoScript-PTEN (white arrow) in the targeted neural cells (cortical spinal neurons). Scale bar: 100 µm (B, C), 10 µm (D)

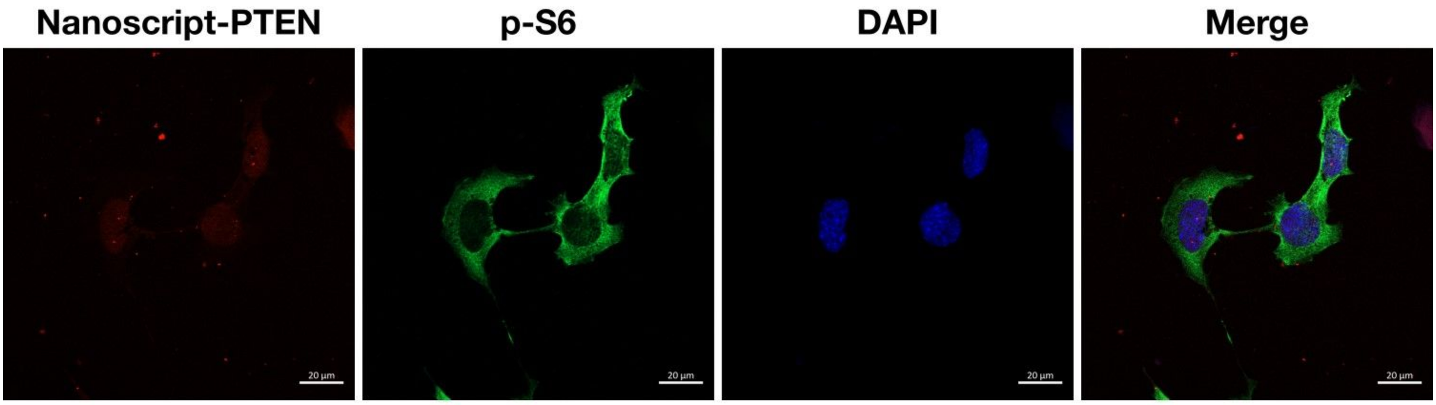


**Fig. S8: NanoScript-PTEN localized in the nucleus of cells expressing p-S6 *in* vivo**. Cells that express an elevated amount of p-S6 have NanoScript-PTEN present in their nucleus. Scale bar: 20 µm

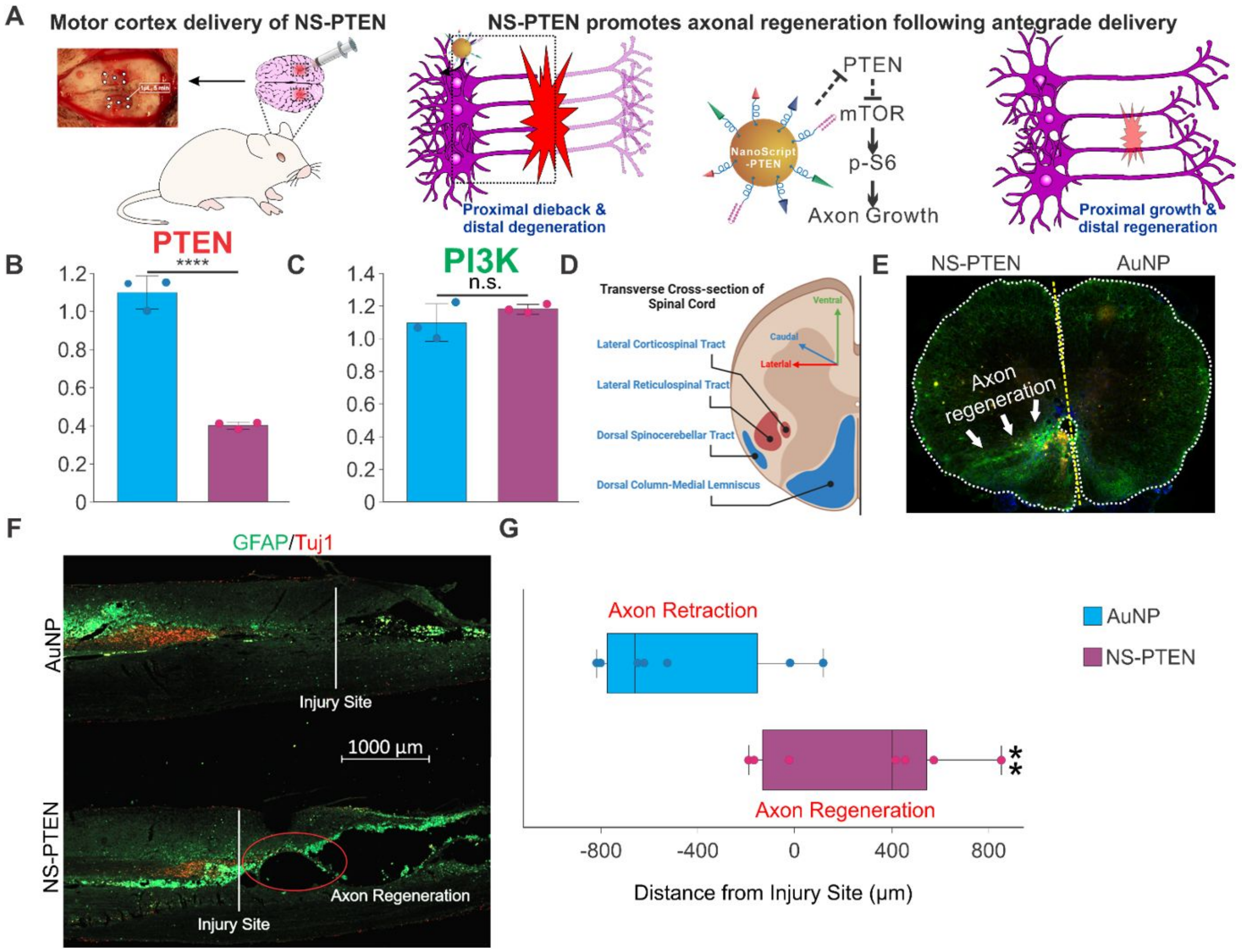


**Fig. S9: Sensorimotor delivery of NanoScript-PTEN induces transient *Pten* repression and promotes corticospinal tract axonal growth following contusion spinal cord injury.** (**A**) Schematic illustration and representative image depicting intracortical delivery of NanoScript-PTEN into the sensorimotor cortex following craniotomy, and the proposed downstream effects on injured corticospinal neurons. (**B** and **C**) Quantitative RT–PCR analysis of *Pten* and *Pi3k* mRNA expression in cortical tissue following NanoScript-PTEN or AuNP control delivery. (**D**). Schematic indicating the anatomical level analyzed in the spinal cord. (**E**) Representative transverse cross-sections of the rat spinal cord collected 3 mm rostral to the injury site, corresponding to the region depicted in (D). Corticospinal tract (CST) axons were visualized by immunostaining for protein kinase C gamma (PKC-γ, green); nuclei were counterstained with Hoechst (blue). The NS-PTEN–treated hemisphere exhibits increased CST axonal density proximal to the lesion compared with the AuNP control. (**F**) Representative sagittal spinal cord sections from rats treated with AuNP control or NanoScript-PTEN following T10 contusion injury, demonstrating CST axonal growth toward and into the lesion region. White lines denote the rostral margin of the injury cavity, and the red oval highlights CST axons extending beyond this boundary. Scale bar, 1000 µm. (**G**) Quantification of the distance of BDA–labeled CST axons relative to the rostral edge of the injury site. Positive values indicate axonal extension beyond the lesion margin, whereas negative values reflect axonal retraction. Data are presented as mean ± SEM. Statistical significance was determined using unpaired two-tailed Student's t-test or one-way ANOVA with Tukey's post hoc test, as appropriate. ****P < 0.0001; **P < 0.01; n.s., not significant.

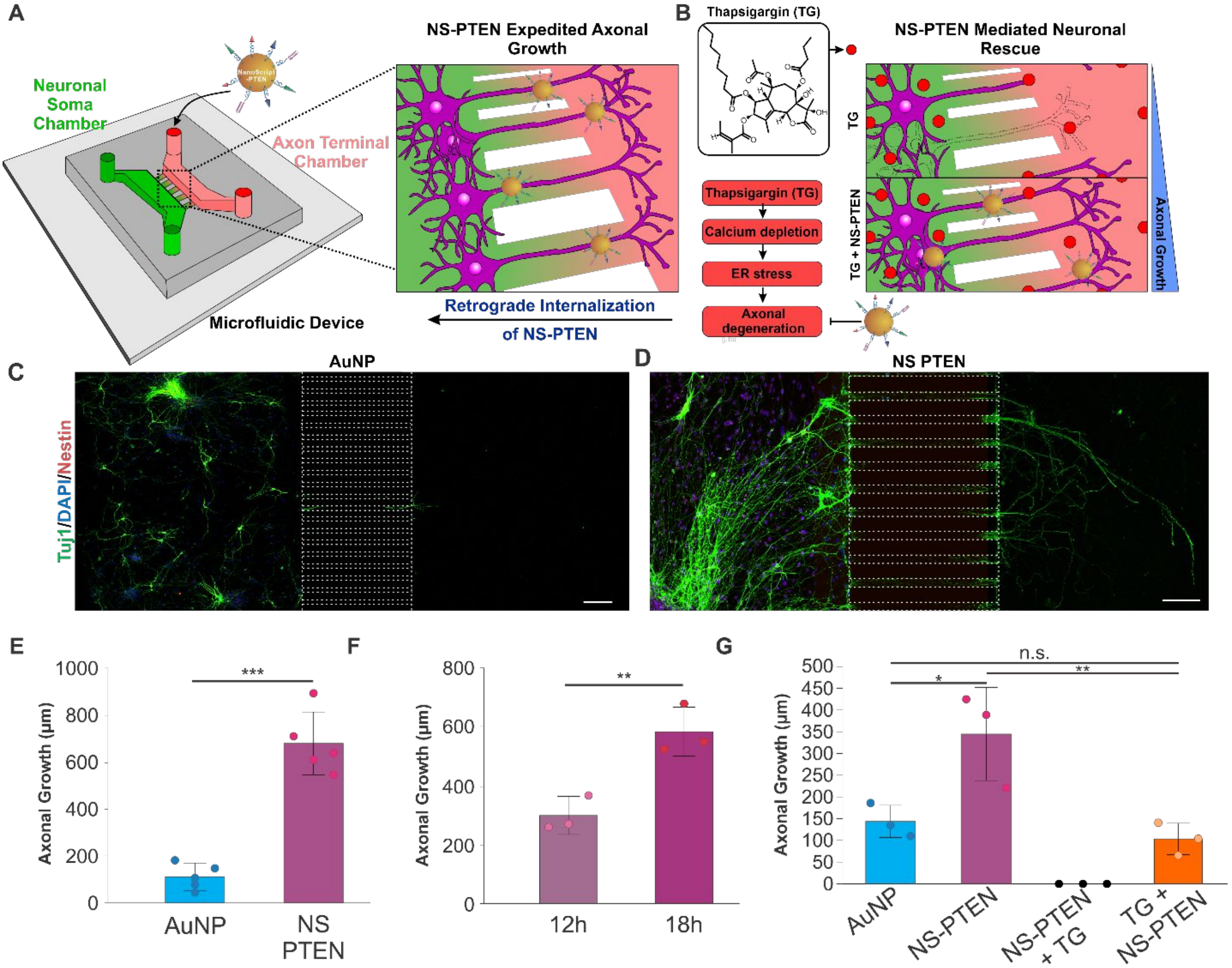


**Fig. S10: NanoScript-PTEN promotes axonal regeneration following emulated spinal cord injury in a microfluidic model.** (**A**) Schematic illustration of fabricated microfluidic device and cellular orientation in corresponding chambers and channels. (**B**) Schematic of neurotoxin-mediated axonal damage used to emulate SCI. (**C** and **D**) Axonal growth through the microfluidic channel into the distal chamber following AuNP control and NanoScript-PTEN delivery. Scale bar, 250 µm. (**E**) Quantification of axonal growth into the distal chamber (*n* = 3). (**F**) Temporal growth of axons over 18 hours following NS-PTEN treatment (*n* = 3). (**G**) NS-PTEN's ability to rescue neurons and promote axonal growth following neurotoxin delivery (*n* = 3). Mean ± SEM, one-way ANOVA, Tukey's test, and t-test. **P* < 0.05; ***P* < 0.01; ****P* < 0.001; n.s. not significant.

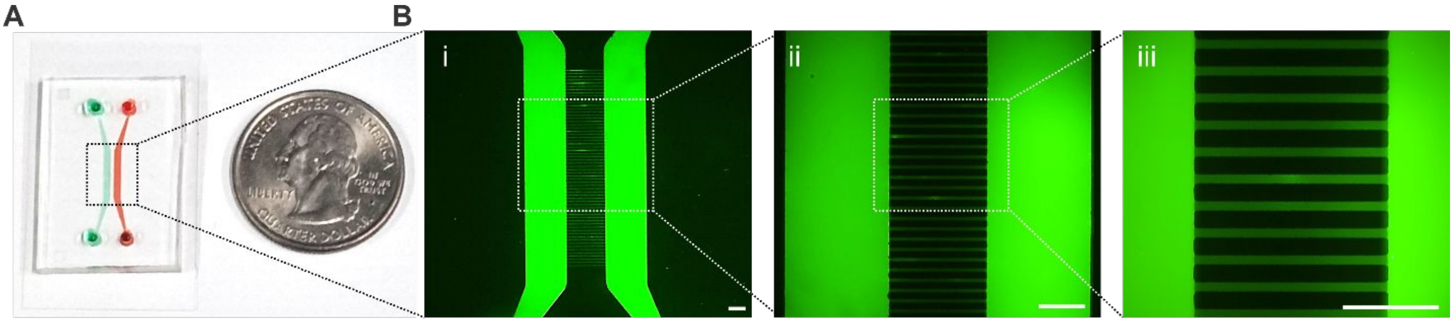


**Fig. S11: Design of microfluidic device**. (**A**) image of a fully fabricated device near a quarter for size comparison. (**B**) Fluorescent images of the microfluidic device. Scale bars are 500 µm

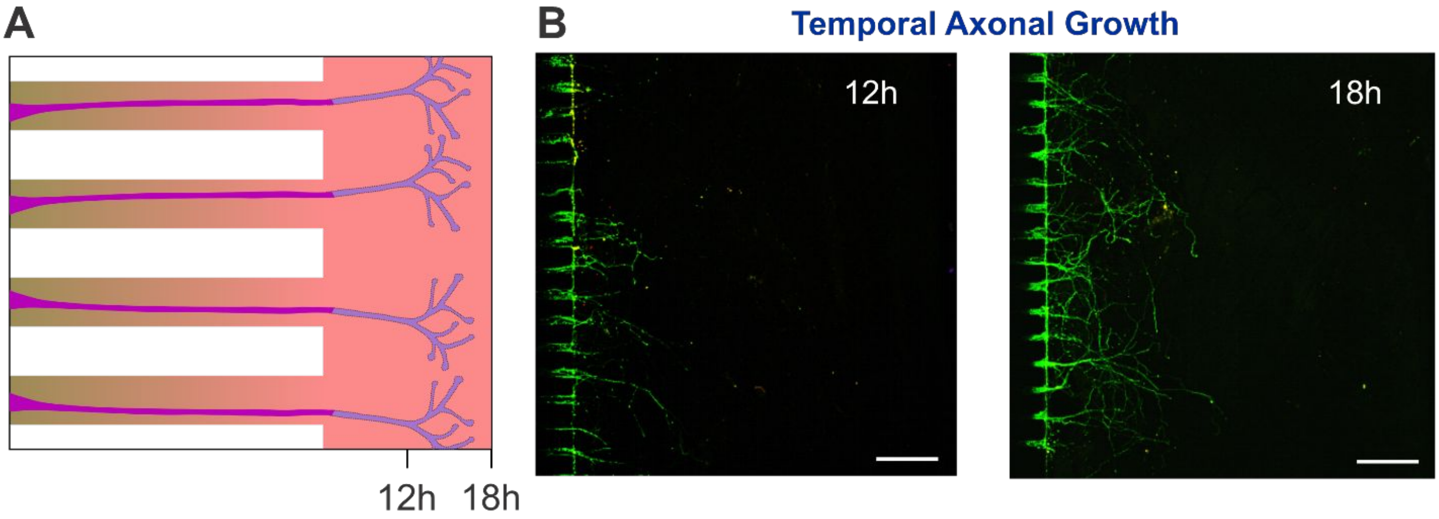


**Fig. S12: Temporal growth of axons following NS-PTEN treatment**. Fluorescent images of neural stem cells treated with NS-PTEN for 12 hours or 18 hours in the microfluidic device. Scale bars are 250 µm.

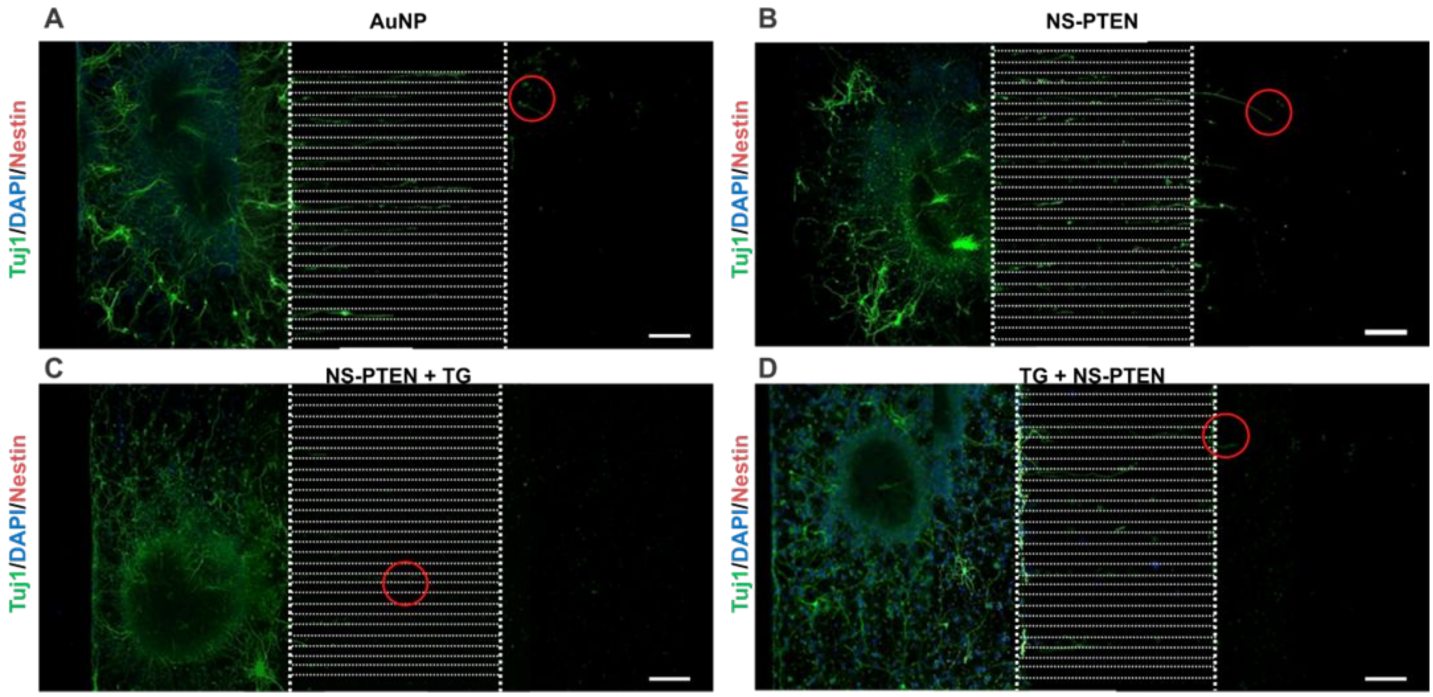


**Fig. S13: Axonal injury and regeneration in the microfluidic device.** Fluorescent images of (A) control, (B) NS-PTEN treatment, (C) thapsigargin treatment after NS-PTEN treatment, and (D) PTEN-NanoScript treatment after thapsigargin treatment in the microfluidic device. The red circle indicates the end of the axon. Scale bars are 200 μm.

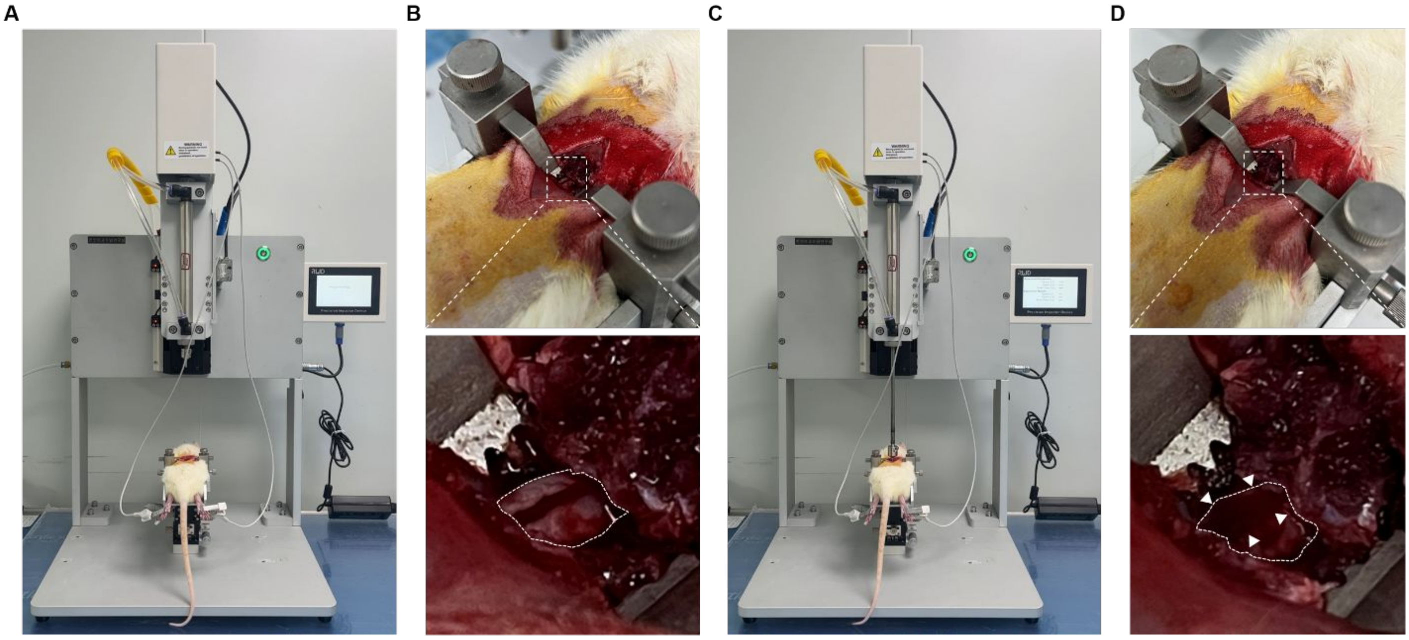

**Fig. S14: Induction of a standardized contusive SCI in SD rats using a precise impactor.** (**A**) After laminectomy (T10) to expose the spinal cord, the anesthetized rat was placed on the spinal cord adaptor of the precise impactor. (**B**) The intact spinal cord, free of dura tears or pre-existing abnormalities, was positioned directly under the impactor tip. (**C**) A controlled contusive SCI was generated according to the predefined parameters, ensuring consistent impact depth, velocity, and dwell time across animals. (**D**) Immediately after injury, a characteristic hemorrhagic lesion appeared at the epicenter (white arrow), confirming successful induction of a contusion model. Dashed outlines indicate the exposed cord surface and the lesion boundaries.

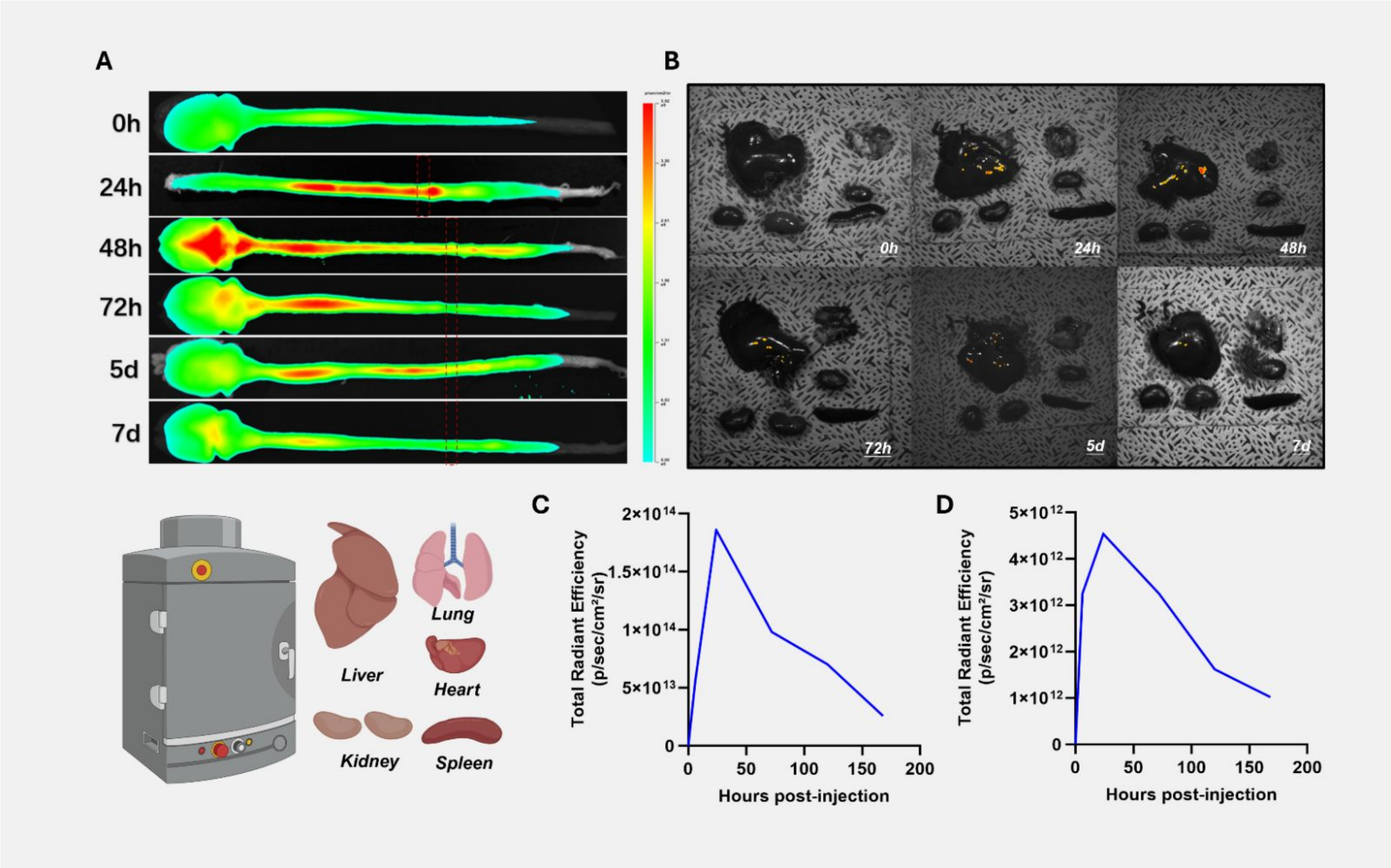


**Fig. S15: Spatiotemporal biodistribution and clearance kinetics of NS-PTEN.** (**A**) Representative ex vivo fluorescence images of the spinal cords harvested at predetermined time points (0–168 h) following NS-PTEN injection. The red dashed rectangles indicate the SCI epicenter. (**B**) Representative ex vivo fluorescence images of major organs (heart, liver, spleen, lung, and kidney) displaying the tissue distribution of NS-PTEN. (**C, D**) Quantitative analysis of the Total Radiant Efficiency (p/sec/cm²/sr) in the spinal cord (**C**) and the organ (**D**) over time.

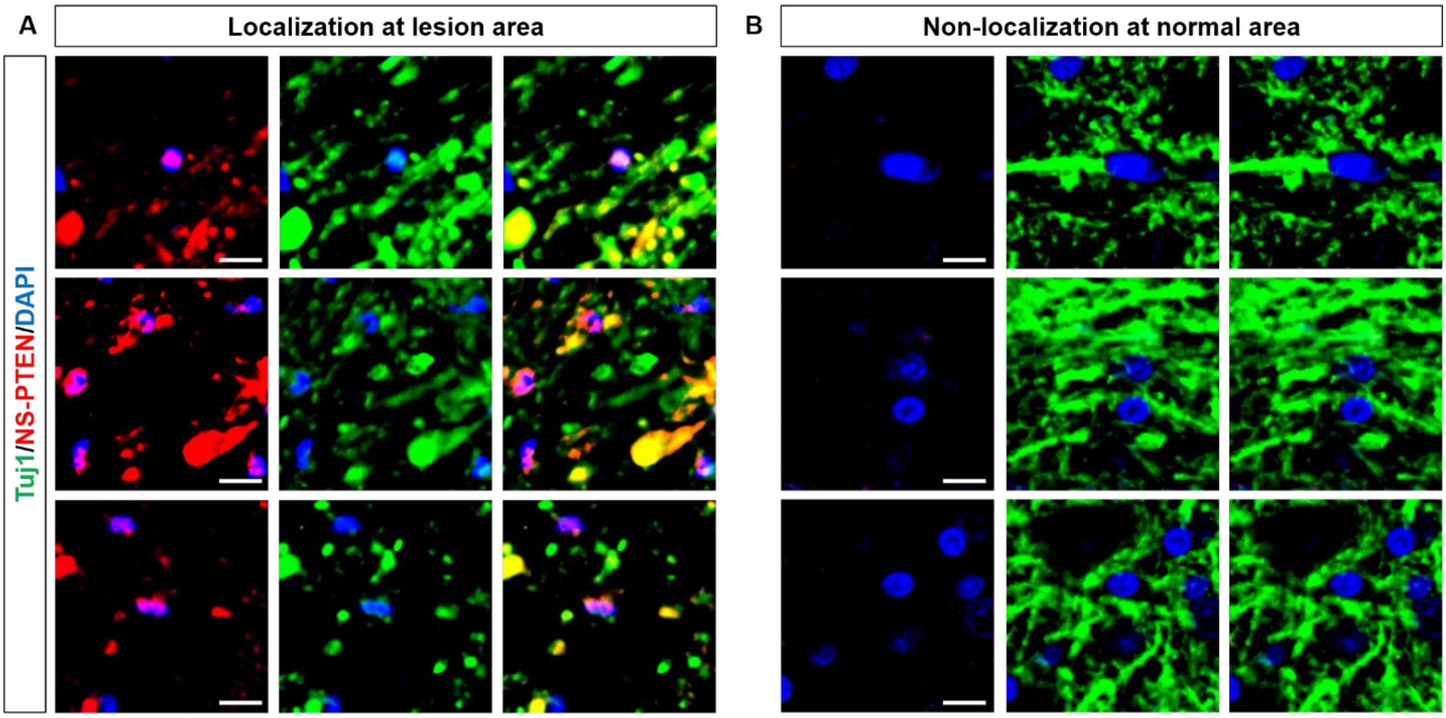


**Fig. S16: Colocalization of NS-PTEN with the nucleus at DPI-1.** (**A**) NS-PTEN overlapped with the nucleus of the injured neuron. (**B**) The nucleus of the normal neuron did not overlap with NS-PTEN at the normal area.

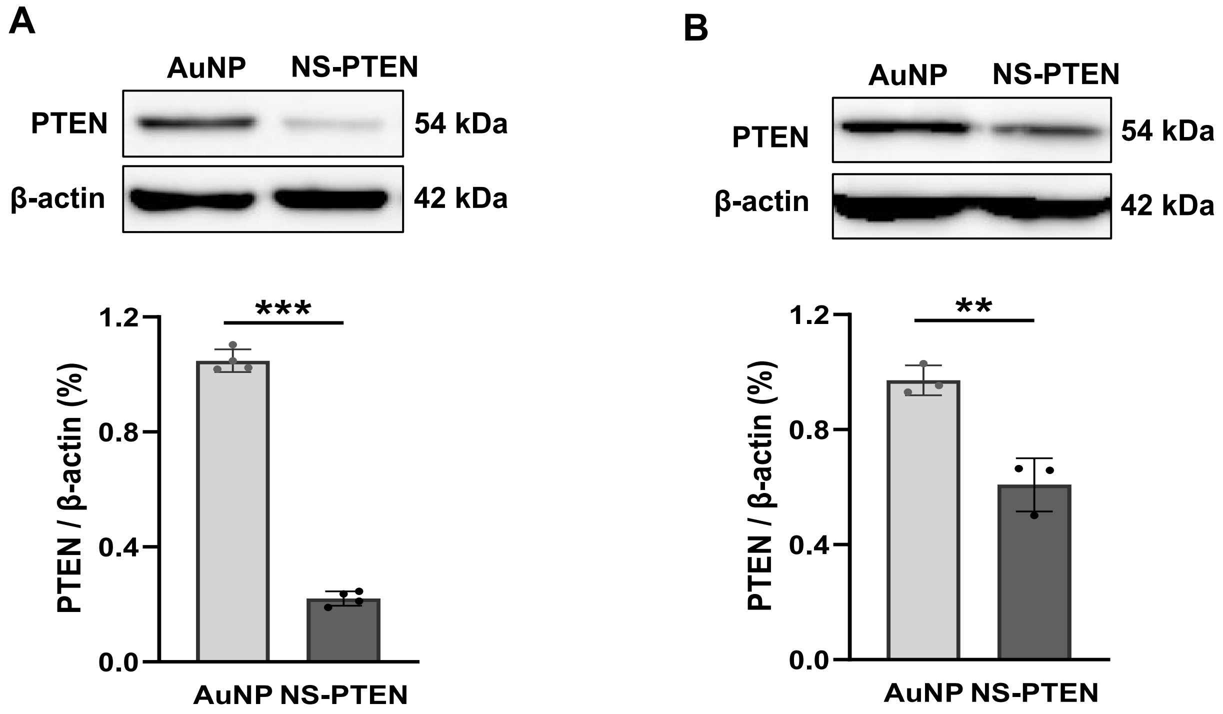


**Fig. S17: Change of PTEN expression with AuNP and NS-PTEN overtime.** (up) Representative image and (down) quantitative data of western blot for PTEN at **(A)** DPI-3 and **(B)** DPI-28. Mean ± SEM, t-test $**P < 0.01$; $***P < 0.001$.

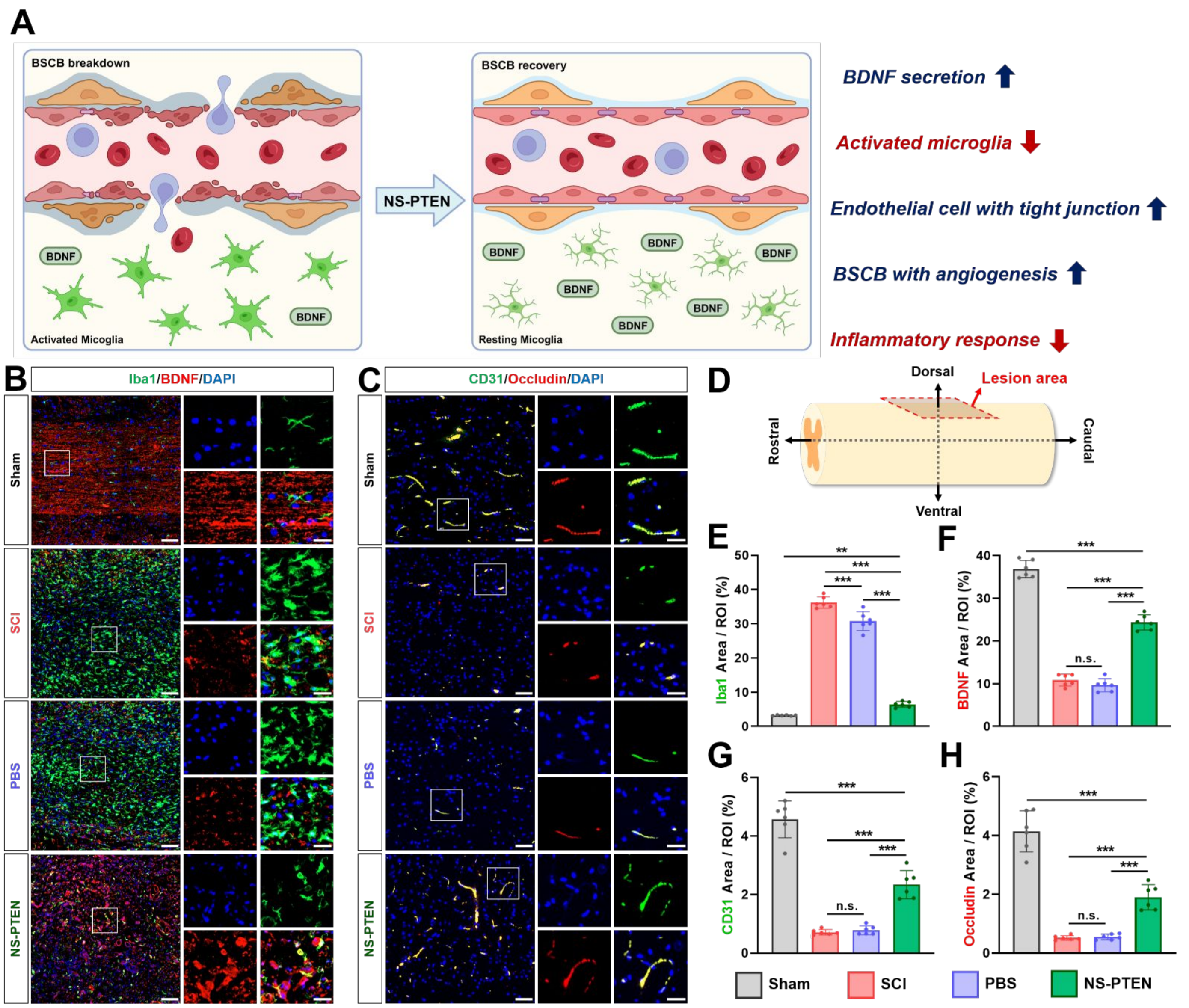


**Figure S18. NS-PTEN attenuates inflammation and promotes blood-spinal cord barrier (BSCB) recovery in the chronic phase of spinal cord injury. (A)** Schematic illustration summarizing the therapeutic mechanism of NS-PTEN, highlighting BSCB restoration, reduced microglial activation, and enhanced BDNF secretion. **(B)** Representative immunofluorescence images of the spinal cord lesion area (indicated in **D**) at 28 days post-injury (DPI-28). Sections were stained for microglia (Iba1, green), neurotrophic factors (BDNF, red), and nuclei (DAPI, blue). Scale bars: 50 µm (main images) and 20 µm (magnified insets). **(C)** Representative immunofluorescence images of the lesion area at DPI-28. Sections were stained for endothelial cells (CD31, green), tight junction proteins (Occludin, red), and nuclei (DAPI, blue). Scale bars: 50 µm (main images) and 20 µm (magnified insets). (**D**) Schematic diagram of the in vivo SCI model. The red dashed box indicates the region of interest (ROI) within the longitudinal spinal cord section used for analysis. **(E–H)** Quantitative analysis of the immunofluorescence area per ROI for **(E)** Iba1, **(F)** BDNF, **(G)** CD31, and **(H)** Occludin. Data are presented as mean ± SEM. Statistical significance was determined by one-way ANOVA followed by Tukey's test. $**P < 0.01$, $***P < 0.001$; n.s., not significant.

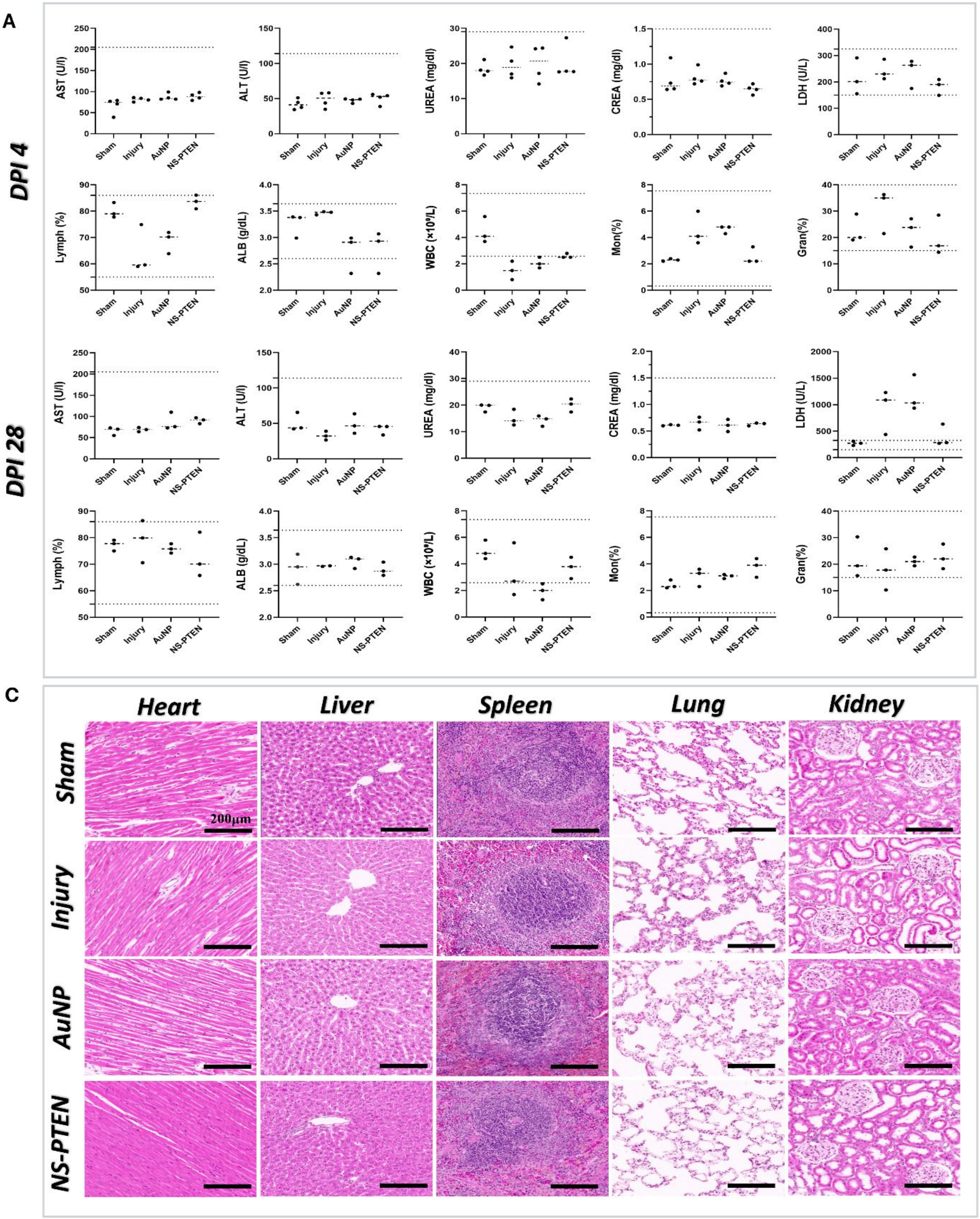


**Fig. S19: Systemic biocompatibility evaluation of NS-PTEN.** (**A**) Representative H&E stained sections of major organs (heart, liver, spleen, lung, and kidney) harvested at dpi 28. Images reveal normal tissue morphology with no evidence of pathological lesions or inflammatory cell infiltration across all groups. (**B, C**) Quantification of serum biochemical markers and hematological parameters at (**B**) acute and (**C**) chronic phases. Indicators of liver function (AST, ALT, LDH), kidney function (Urea, Creatinine), and blood cell counts (WBC, Lymphocytes, Monocytes, Granulocytes) were analyzed. Dashed lines indicate physiological reference ranges.

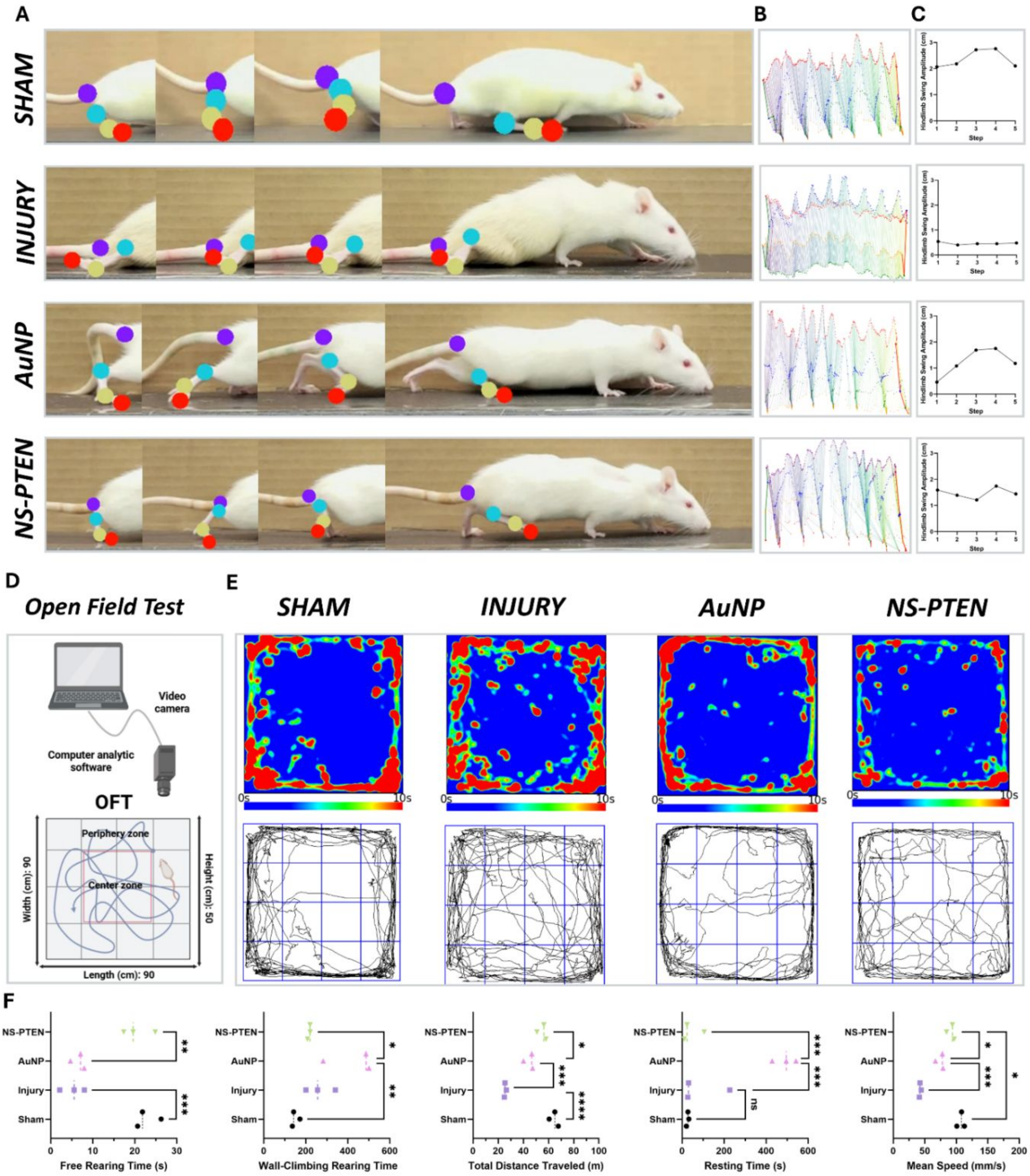


**Figure S20. Assessment of locomotor kinematics and spontaneous exploratory behavior at DPI-28.** (A) Schematic representation of markerless pose estimation using DeepLabCut to track hindlimb vertical excursion and stepping kinematics. (B) Representative vertical hindlimb swing amplitude traces across groups. (C) Quantification of mean hindlimb swing amplitude (cm), indicating the quality of gait recovery. (D–E) Representative occupancy heat maps (D) and corresponding locomotor trajectories (E) during a 10-minute Open Field Test (OFT). In heat maps, red and blue regions denote high- and low-frequency occupancy, respectively. Note the transition from thigmotaxis in the Injury group to expanded central zone exploration in the NS-PTEN group. (F) Quantitative analysis of OFT parameters, including total distance traveled (m), mean speed (mm/s), free rearing time (s), wall-climbing rearing time (s), and resting time (s). Data are presented as mean ± SD. Statistical significance was determined using ANOVA followed by Tukey's post hoc test. *P <

0.05, **P < 0.01, ***P < 0.001, ****P < 0.0001; ns, not significant.

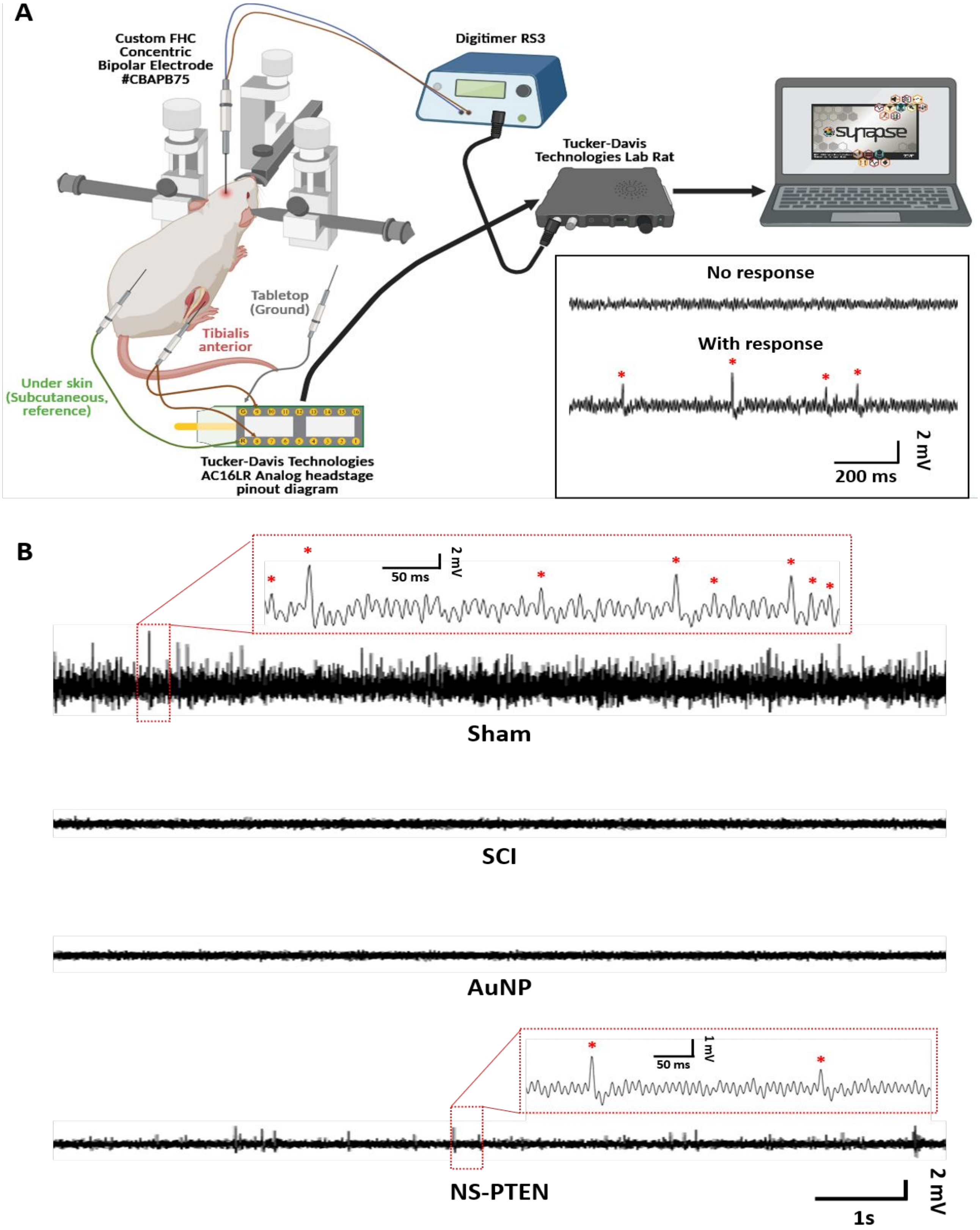


**Figure S21. NS-PTEN promotes recovery of motor-evoked potential after spinal cord injury.** NS-PTEN encourage the recovery of the motor evoked potential (MEP) recorded from the tibialis anterior of rats subjected to SCI. (A) Schematic of the experimental layout and wired connections of the hardware used in the MEP recording in rat. Example of a recording with and without responses is provided as an insert. (B) Recordings from rats subjected to sham (control) or SCI are presented. Animals subjected to SCI are further treated with two different synthetic materials to test their impact on the recovery of the SCI. Traces with

responses NS-PTEN are zoomed in (inserts) for better visualization of the responses observed. Responses are marked with red asterisk.

**Table S1. Western blot antibody information**

| [a] Primary antibody | Catalog number | Manufacturer | Dilution |
|---|---|---|---|
| PTEN | 22034-1-AP | Proteintech | 1:1000 |
| β-actin | Ab6276 | Abcam | 1:5000 |
| P-AKT (Ser473) | CST 9271 | CST | 1:1000 |
| AKT | Proteintech 60203-2-Ig | Proteintech | 1:1000 |
| P-mTOR (Ser2448) | CST 5536 | CST | 1:1000 |
| mTOR | CST 2983 | CST | 1:1000 |
| P-GSK-3β (Ser9) | CST 9336 | CST | 1:1000 |
| GSK-3β | CST 9315 | CST | 1:1000 |
| P-STAT3 (Tyr705) | CST 9145 | CST | 1:1000 |
| STAT3 | CST 12640 | CST | 1:1000 |
| **[b] Secondary antibody** | **Catalog number** | **Manufacturer** | **Dilution** |
| Goat anti-Rabbit IgG-HRP | 31460 | Invitrogen | 1:10000 |
| Goat anti-Mouse IgG-HRP | 31430 | Invitrogen | 1:10000 |

[a] Primary antibody and [b] Secondary antibody for western blot

Abbreviations: PTEN; Phosphatase and tensin homolog

**Table S2. Immunofluorescence antibody information**

| a) Primary antibody | Catalog number | Manufacturer | Dilution |
|---|---|---|---|
| TUJ-1 | Ab78078 | Abcam | 1:200 |
| MBP | 78896S | CST | 1:200 |
| NF | ab8135 | Abcam | 1:800 |
| GFAP | MAB360 | Sigma-Aldrich | 1:400 |
| GFAP | ab7260 | Abcam | 1:1000 |
| Iba-1 | 17198S | CST | 1:500 |
| Iba-1 | ab5076 | Abcam | 1:250 |
| BDNF | AGP-021 | Alomone Labs | 1:300 |
| CD31 | Ab281583 | Abcam | 1:400 |
| Occludin | 33-1500 | Thermo Fisher | 1:50 |
| CD68 | ab31630 | Abcam | 1:200 |
| CD163 | ab182422 | Abcam | 1:300 |
| NeuN | ab104224 | Abcam | 1:1000 |
| 5-HT | 20080 | Immnunostar | 1:1000 |
| GAP43 | 33-5000 | Invitrogen | 1:100 |
| **b) Secondary antibody** | **Catalog number** | **Manufacturer** | **Dilution** |
| Goat anti-Rabbit IgG (Alexa Fluor® 488) | A-11034 | Thermo Fisher | 1:200 |
| Goat anti-Mouse IgG (Alexa Fluor® 488) | A-11029 | Thermo Fisher | 1:200 |
| Goat anti-Rabbit IgG (Alexa Fluor® 568) | A10042 | Thermo Fisher | 1:200 |
| Goat anti-Mouse IgG (Alexa Fluor® 568) | A11004 | Thermo Fisher | 1:200 |
| Goat anti-Guinea pig IgG (Alexa Fluor® 568) | ab175714 | Abcam | 1:200 |
| Goat anti-goat IgG (Alexa Fluor® 568) | A11057 | Thermo Fisher | 1:200 |

a) Primary antibody and b) Secondary antibody for immunofluorescence

Abbreviations: TUJ-1 (Neuron-specific class III β-tubulin), MBP (Myelin Basic Protein), NF (Neurofilament), GFAP (Glial Fibrillary Acidic Protein), Iba-1 (Ionized Calcium-Binding Adapter Molecule 1), BDNF (Brain-Derived Neurotrophic Factor), CD31 (Platelet/Endothelial Cell Adhesion Molecule-1), Occludin (Tight Junction Protein Occludin), CD68

(Cluster of Differentiation 68), CD163 (Cluster of Differentiation 163), NeuN (Neuronal Nuclei), 5-HT (5-Hydroxytryptamine/Serotonin), GAP43 (Growth Associated Protein 43).

**Table S3. Primers for experiments**

| Gene | Forward | Reverse |
|---|---|---|
| PTEN | TTTGAAGACCATAACCCACCAC | ATTACACCAGTTCGTCCCTTT |
| PI3K | GAAACAAGACGACTTTGTGACCT | CTTCACGGTTGCCTACTGGT |
| AKT | TCTGGAGCAGTATTACGACCC | CTGGCTGGAATCTAGCAGTCT |
| GSK-3β | AGACGCTCCCTGTGATTTATGT | CCGATGGCAGATTCCAAAGG |
| GAP43 | GCTACCACTGATAACTCGCC | GGCTTCATCTACAGCTTCTTTCT |
| iNOS | ACCACTCGTACTTGGGATGC | CACCTTGGAGTTCACCCAGT |
| TNF-α | CTGCTGCACTTTGGAGTGAT | AGATGATCTGACTGCCTGGG |
| IL-6 | AAACAACCTGAACCTTCCAAAGA | GCAAGTCTCCTCATTGAATCCA |
| mTOR | TGCTGTCAACAACACAGCCG | CAGGTTGGATGGGTGCCTGT |
| Creb1 | TGACGGAGGAGCTTGTACCAC | TGGCTGGGCTTGAACTGTCAT |
| IL-1β | ATGATGGCTTATTACAGTGGCAA | GTCGGAGATTCGTAGCTGGA |
| GAPDH | CATGTTCCAATATGATTCCACC | GATGGGATTTCCATTGATGAC |

Abbreviations: PTEN (phosphatase and tensin homolog), PI3K (phosphoinositide 3-kinase), AKT (protein kinase B), GSK-3β (glycogen synthase kinase-3 beta), GAP43 (growth-associated protein 43), iNOS (inducible nitric oxide synthase), TNF-α (tumor necrosis factor-alpha), IL-6 (interleukin-6), mTOR (mechanistic target of rapamycin), Creb1 (cAMP-responsive element-binding protein 1), IL-1β (interleukin-1 beta), and GAPDH (glyceraldehyde-3-phosphate dehydrogenase)
